# A p53 transcriptional signature in primary and metastatic cancers derived using machine learning

**DOI:** 10.3389/fgene.2022.987238

**Published:** 2022-08-29

**Authors:** Faeze Keshavarz-Rahaghi, Erin Pleasance, Tyler Kolisnik, Steven J. M. Jones

**Affiliations:** ^1^ Canada’s Michael Smith Genome Sciences Centre, BC Cancer, Vancouver, BC, Canada; ^2^ Department of Bioinformatics, University of British Columbia, Vancouver, BC, Canada; ^3^ School of Natural and Computational Sciences, Massey University, Auckland, New Zealand; ^4^ Department of Medical Genetics, University of British Columbia, Vancouver, BC, Canada; ^5^ Department of Molecular Biology and Biochemistry, Simon Fraser University, Vancouver, BC, Canada

**Keywords:** p53 pathway activation, transcriptome, pan-cancer, machine learining, random forest, ensemble classifier

## Abstract

The tumor suppressor gene, *TP53*, has the highest rate of mutation among all genes in human cancer. This transcription factor plays an essential role in the regulation of many cellular processes. Mutations in *TP53* result in loss of wild-type p53 function in a dominant negative manner. Although *TP53* is a well-studied gene, the transcriptome modifications caused by the mutations in this gene have not yet been explored in a pan-cancer study using both primary and metastatic samples. In this work, we used a random forest model to stratify tumor samples based on *TP53* mutational status and detected a p53 transcriptional signature. We hypothesize that the existence of this transcriptional signature is due to the loss of wild-type p53 function and is universal across primary and metastatic tumors as well as different tumor types. Additionally, we showed that the algorithm successfully detected this signature in samples with apparent silent mutations that affect correct mRNA splicing. Furthermore, we observed that most of the highly ranked genes contributing to the classification extracted from the random forest have known associations with p53 within the literature. We suggest that other genes found in this list including GPSM2, OR4N2, CTSL2, SPERT, and RPE65 protein coding genes have yet undiscovered linkages to p53 function. Our analysis of time on different therapies also revealed that this signature is more effective than the recorded *TP53* status in detecting patients who can benefit from platinum therapies and taxanes. Our findings delineate a p53 transcriptional signature, expand the knowledge of p53 biology and further identify genes important in p53 related pathways.

## Introduction

The most frequently somatically mutated gene in human cancer is *TP53* which encodes the p53 protein, signifying the importance of its wild-type function in tumor suppression (Levine and Oren, 2009; Kandoth et al., 2013; Duffy et al., 2014; Donehower et al., 2019; Thomas et al., 2022). Wild-type p53 functions as a transcription factor activated in response to cellular stresses (Giaccia and Kastan, 1998; Prives and Hall, 1999; Vousden and Lu, 2002; Vousden and Prives, 2009; Duffy et al., 2014; Thomas et al., 2022). The protein function can be compromised via various mechanisms (Shvarts et al., 1996; Kostic et al., 2006; Vassilev, 2007; Duffy et al., 2014), the most common being missense mutations followed by a loss of heterozygosity resulting in the complete loss of wild-type p53 function (Levine and Oren, 2009; Duffy et al., 2014; Donehower et al., 2019; Mantovani et al., 2019). Mutations in *TP53* are associated with a poor prognosis in many cancers and germline mutations in this gene cause the Li-Fraumeni syndrome which increases the susceptibility to diverse cancer types (Malkin et al., 1979; Srivastava et al., 1990; Kandoth et al., 2013; Donehower et al., 2019; Mantovani et al., 2019).

Machine learning (ML) approaches have been used to investigate large and complex data sets, including the classification of cancer types, the determination of informative features in cancer diagnosis, and the analysis of *TP53* mutations and their effects (Danziger et al., 2009; Chitrala et al., 2019; Grewal et al., 2019; Lim et al., 2019; Banerjee and Mitra, 2020; Yuan et al., 2020). Transcript expression data has been used to classify primary tumors and breast cancer subtypes based on *TP53* mutational status (Benor et al., 2020; Saghaleyni et al., 2021; Zhang et al., 2021). Subsets of The Cancer Genome Atlas (TCGA) samples have been successfully stratified based on aberrant p53 pathway activities (Zhang et al., 2021). In all of these studies, filtering and data reduction were applied to both samples and gene sets. To our knowledge, the effects of *TP53* mutations on the transcriptome have not been investigated in a pan-cancer study using both primary and metastatic samples without applying specific filters on the sample types and the genes.

In this work, we show how a transcriptional signature for loss of p53 function can be detected using ML approaches. We trained a random forest (RF) algorithm using primary tumor TCGA expression and mutation data (Kandoth et al., 2013; Weinstein et al., 2013) and metastatic tumor data from the British Columbia (BC) Cancer Agency Personalized OncoGenomics (POG) program (Pleasance et al., 2020). In this pan-cancer analysis, all coding and non-coding genes identified in both TCGA and POG datasets were included. We were able to show that our model could predict the *TP53* mutational status of tumors accurately and precisely from transcriptome expression levels alone. The list of genes contributing most to classification in the model correlated highly with those genes known to be involved in p53 function and biology. Additionally, we showed that the model could correctly categorize the samples with synonymous somatic mutations at splice sites in *TP53* as pathogenic. Our results also showed that combining all tumor types within the training set improved the overall accuracy and specificity of predictions. This indicates that a general transcriptional signature of p53 functional loss exists, is detectable and is conserved across tumor types. This signature can aid in identifying patients who can benefit from different therapies through recognition of the transcriptional patterns that are associated with p53 pathways disruptions. Due to variable response to different drug regimens, side effects, and resistance, there is a need for personalized therapies (Daly, 2010; Wilke and Dolan, 2011; Gerlinger et al., 2012; Dey et al., 2017; Hyman et al., 2017) to increase the success of treatment and improve patient outcomes especially in metastatic disease (Gerlinger et al., 2012; Dey et al., 2017).

## Methods

Expression matrices and mutation data were obtained from the TCGA and POG studies (Supplementary Material). Non-primary tumor samples and the samples lacking mutation data from TCGA were excluded. All genes common to both TCGA and POG expression matrices were included. Principal Component Analysis (PCA) plots were generated (Hunter, 2007; Pedregosa et al., 2011; Waskom, 2021). Samples were divided by mutational status (mutated vs. wild-type), and further by impact of mutation (impactful vs. non-impactful). Samples with a mutation of type “silent”, “intron”, “3-prime UTR”, “5-prime UTR”, “downstream gene”, “upstream gene” or “splice region” were classified as “non-impactful”.

### Random forest performance

For this analysis, only samples with “impactful” mutations or wild-type p53 copies were included to increase the likelihood of only pathogenic *TP53* driver alterations being used for training. The main hyperparameters were calibrated using 90% of samples and the obtained values were validated using the remaining 10%. The 90–10 split was performed randomly while maintaining the proportion of *TP53* mutant and wild-type samples. The RF performance was then evaluated across TCGA, POG, and merged (all TCGA and POG samples with wild-type p53 or impactful *TP53* mutations) datasets using 5-fold cross-validation analyses. Precision, recall, F1-score, area under the precision-recall curve (AUPRC), and area under the receiver operating characteristic curve (AUROC) values were found by applying the scikit-learn library functions (Pedregosa et al., 2011). To compare the performance on each cancer cohort individually versus the pan-cancer set, the accuracy scores were compared to the Out of Bag scores which were found by training the model using only the samples in each cancer type. Additionally, performance metrics were calculated across cancer types by clustering the samples in each cohort and their predictions from the previous step and computing the values using the scikit-learn evaluation functions.

### Significant genes in classification

The genes that played a more important role in classification were extracted based on the Gini importance scores of the RF model. The threshold for the number of important genes was found using a permutation-based method (Supplementary Material). The 67 genes meeting this threshold were extracted and used to perform a Gene Set Enrichment Analysis (GSEA) using the Database for Annotation, Visualization, and Integration Discovery (DAVID) (Huang et al., 2009a; Huang et al., 2009b). Cellular pathways correlated with these 67 genes were obtained with a threshold of 0.05 for *p*-values adjusted using the Benjamini–Hochberg procedure (Huang et al., 2009b).

### Prediction probabilities and outliers

The probabilities associated with the RF predictions were extracted from the model and were grouped by prediction correctness, sample source (TCGA and POG), true *TP53* status (label), and the *TP53* status predicted by the RF. Using these likelihoods, mispredicted samples with high prediction probabilities (>0.95) were identified. Two samples (TCGA-AR-A24T-01 and TCGA-VM-A8CH) that belonged to relatively balanced cancer cohorts were investigated. Whole exome sequencing and RNA-seq files of these samples and RNA-seq files of four other comparator brain and breast cancer samples (two with wild-type and two with mutated *TP53* copies) were visualized using the Broad Institute’s Integrative Genomics Viewer (IGV) (Robinson et al., 2011).

### Samples with non-impactful *TP53* mutations

To determine the status of the samples with non-impactful mutations, the merged set was used to train the algorithm, and the status of the non-impactfully mutated samples were predicted. The samples with silent mutations assigned to the p53 mutant category were further investigated, as the expectation would be to see no change in the p53 protein and therefore similar behavior to the p53 wild-type category. The RNA-seq data of these samples was visualized in IGV (Robinson et al., 2011).

### Treatment efficacy in patients with mutated and wild-type p53

Treatment data from the POG cohort was obtained (Pleasance et al., 2020), and drugs were grouped by their mechanism of action and/or target genes or proteins (Antineoplastic Agents, 2012; Vardanyan et al., 2016; Wishart et al., 2018; NCI, 2022). Combination therapies were separated into individual drugs, and data for patients on a double-blind trial where the received treatment was unknown were filtered out. Total days on therapy was used as a proxy for treatment response as response data was not available. Drug groups with <5 patients or only p53 wild-type tumors were excluded.

## Results

Primary tumors from TCGA with available mutation data and metastatic tumors from POG were collated, and 48,784 overlapping coding and non-coding genes were identified. The PCA indicated that samples clustered by cancer type (Supplementary Figures S1A,B) and primary and metastatic samples also clustered together (Supplementary Figure S1C). Since there was no distinctive boundary between TCGA and POG data sets, the samples were merged for classification.

Out of the 8755 TCGA samples, 3,373 (39%) had a mutation in *TP53* and 5,382 (61%) had only wild-type copies of this gene. 47 (1%) of the samples with a mutated *TP53* were classified as “non-impactful” while the other 3,326 (99%) were placed into the “impactful” category. Out of 570 POG samples, 229 (40%) had a mutation in *TP53* and 341 (60%) contained wild-type copies. Among 229 mutated samples, 23 (10%) were categorized as “non-impactful”, and 206 (90%) as “impactful”.

### Random forest performance

**FIGURE 1:**
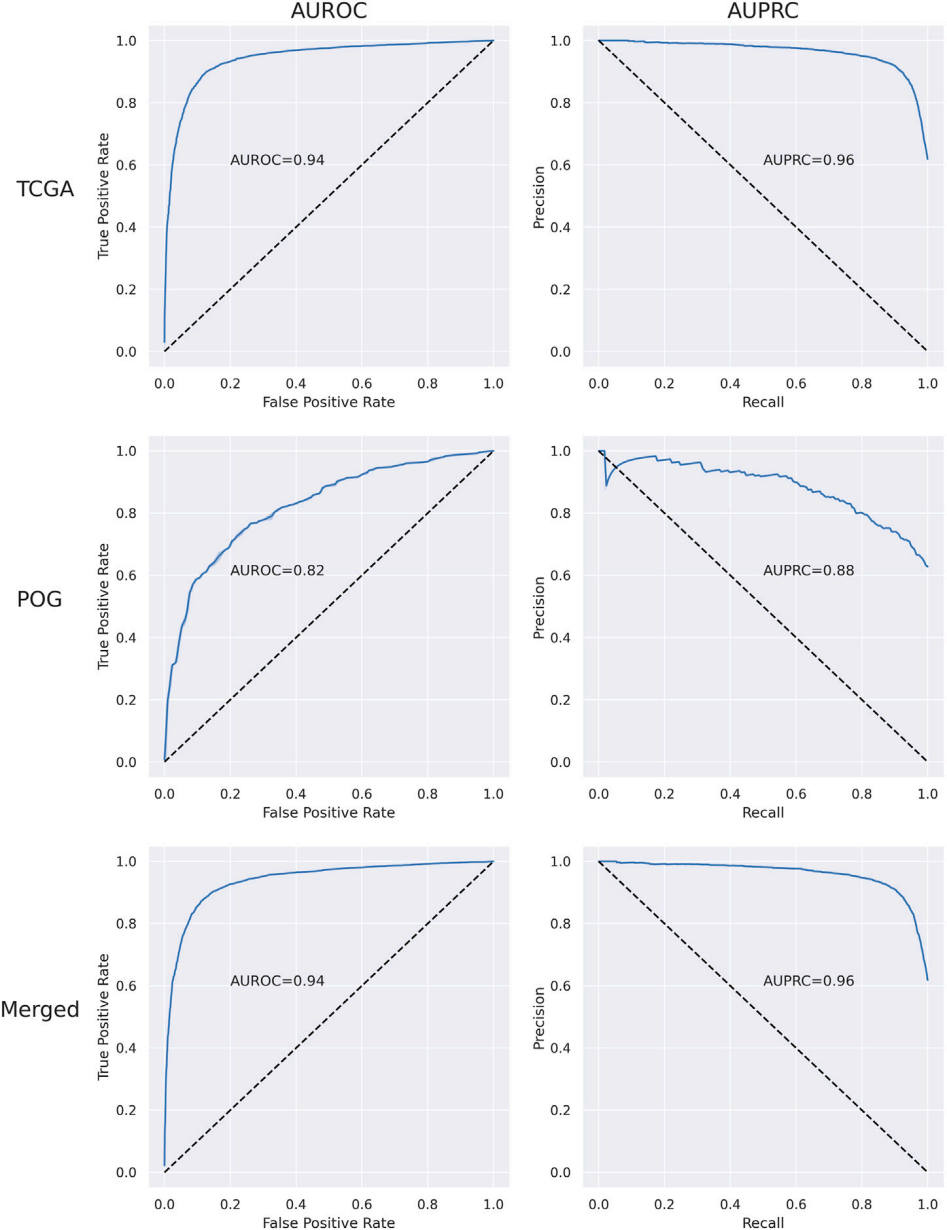
Plots of area under the receiver operating characteristic curve (AUROC) and area under the precision-recall curve (AUPRC) for TCGA, POG, and merged (set of all impactful and wild-type samples) data sets.

The performance of the RF was first optimized by tuning the hyperparameters for the TCGA, POG, and merged data sets and then evaluated using 5-fold cross-validation analyses (Supplementary Table S1 and Supplementary Figures S2–S5). Over 10 independent tests, merging TCGA and POG resulted in a mean of 35 more samples (4 TCGA and 31 POG) successfully classified by the RF, demonstrating benefit in combining the primary and metastatic samples to train the algorithm and detect the transcriptional patterns. Overall, the RF performance was successful with AUROC 0.94 and AUPRC 0.96 in the merged dataset (Supplementary Table S2 and Figure 1). Mean accuracy was 0.88, with precision 0.88, recall 0.87, and f1-score 0.87.

Across the TCGA cancer types, the algorithm classified samples with >0.75 f1-score in most cancer types where the minority class to majority class sample number ratio was greater than 10% (Supplementary Table S3). In highly imbalanced cancer types, sample classification was less successful. The prediction accuracy when all the samples were used for training was better than or very similar to when training was done on individual cancer types for 30 out of 33 tumor categories (Supplementary Figure S6), so it can be concluded that there is a benefit in combining all cancer types to train the RF model.

### Significant genes in classification

**FIGURE 2:**
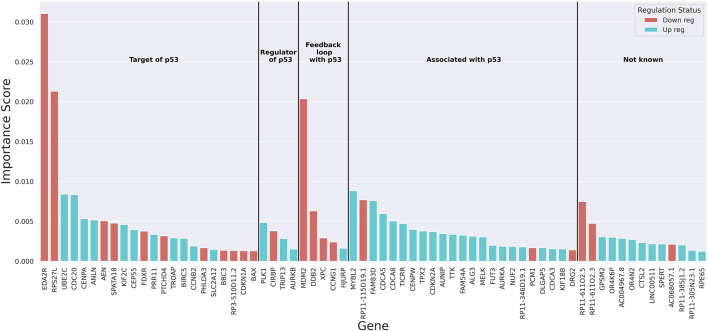
The bar plot of importance (Gini) scores of top 67 genes extracted from the random forest. Red colour indicates downregulation and blue colour shows upregulation of the genes with the loss of wild-type p53 function. Genes are grouped based on their known link to p53.

To understand the underlying decision-making process of the RF algorithm, the genes that played a more important role in classification were extracted using the built-in feature importance scores (Gini importance scores). A threshold of 67 genes was attained using a permutation method (Supplementary Material) as the cut-off for the list of genes contributing to the classification of samples based on *TP53* mutation status (Supplementary Figure S7). The importance scores of these genes as well as the change in their expression with the loss of wild-type p53 function and their known link to p53 based on retrieved literature were obtained (Figure 2 and Supplementary Figure S4). Over- and under-expression of these genes in the absence of wild-type p53 function was observed to be entirely aligned with what is known about the regulation of these genes (Supplementary Figure S4). For example, the three genes with the highest importance scores in the classification have all been shown to be upregulated in the presence of wild-type p53 activity (Piette et al., 1997; Tanikawa et al., 2010; Xiong et al., 2011; Donehower et al., 2019; Moyer et al., 2020). The findings from other studies on p53 targets using experimental approaches further confirmed the relevance of our gene list. Out of the 27 genes known to be regulated by p53 in our list (targets of p53 and genes that are in a feedback loop with p53), 10 were found in the list of 122 p53-regulated genes by Riley et al. (*p*-value = 8.9 × 10^−16^ from a hypergeometric test with N = 18,337 (protein-coding genes in our data), k = 122, n = 27, and x = 10) (Riley et al., 2008). Additionally, 9 out of the 27 genes regulated by p53 were found in the list of 46 genes bound by p53 identified by Nikulenkov et al. (*p*-value = 7.7 × 10^−18^ from a hypergeometric test with N = 18,337, k = 46, n = 27, and x = 9) (Nikulenkov et al., 2012). Expression of our model’s top 10 genes (Supplementary Figure S8) confirmed an overall difference in the transcriptional behavior of these genes between the mutant and wild-type classes. GSEA revealed these 67 genes were most enriched in cell cycle pathways (P_adj_ < 8.9 × 10^−22^), which are expected to be affected by *TP53* mutations (Huang et al., 2009a; Huang et al., 2009b) (Supplementary Table S5). Lastly, to recognize the potential role of the *TP53* transcript itself in the classification, the rank of *TP53* in the model’s important genes list was obtained in 100 independent training iterations. This rank on average was 109 (±6 standard deviation) with a median of 109 (IQR: 105–114), indicating that the transcriptional level of the *TP53* gene itself did not significantly contribute to the classification.

### Prediction probabilities and outliers

Correctly predicted samples had a higher median prediction probability, indicating higher confidence compared to mis-predicted samples (P_adj_ < 1 × 10^−4^) (Supplementary Figure S9). Prediction probabilities for TCGA were higher than for POG, likely due in part to the much larger number of samples included in training the algorithm. Furthermore, it was noted that the wild-type samples for p53 had higher probabilities compared to samples with mutated *TP53* genes. This could be due to the larger number of wild-type p53 samples in the training set or could indicate that there is a more dominant signature of wild-type p53 function across different cancer types.

Out of 1,402 samples with a prediction probability of more than 0.95, 14 (1%) were found to be incorrectly classified (Supplementary Table S6). The majority of these samples belonged to highly imbalanced cancer cohorts. However, two samples belonged to relatively balanced cohorts (TCGA-VM-A8CH-01, brain lower grade glioma, and TCGA-AR-A24T-01, breast invasive carcinoma). Inspection of the RNA-seq alignments revealed that the TCGA-VM-A8CH-01 contained a missense mutation at the 277th amino acid of p53 that changes cysteine to phenylalanine (p.C277F) (Supplementary Figure S10A). Whole exome sequencing data showed that both tumor and normal samples of this patient contained this single nucleotide variation and thus the mutation was germline (the sample was initially mislabeled as wild-type due to lack of a somatic mutation). Based on the *TP53* Database (R20 July 2019: https://tp53.isb-cgc.org) (Bouaoun et al., 2016), it is believed that p. C277F is a pathogenic, non-functional mutation (i.e., a loss-of-function mutation). Our findings confirm the loss of p53 activity and suggest that this mutation might play a role in cancer predisposition. The label of this lower grade glioma sample was then changed to p53 mutant, and the algorithm performance was inspected again. No change was observed in the performance metrics which shows that the RF model is already robust to noise.

The whole exome sequencing and RNA-seq data for TCGA-AR-A24T-01 breast invasive carcinoma confirmed the existence of a p. R273H mutation in *TP53* (Supplementary Figure S10B) even though it was classified as wild-type tumor by the RF. The misprediction does not seem to be related to the specific mutation because out of the 101 TCGA and POG samples with the p. R273H mutation in our data, 89 (88%) were correctly assigned to p53 mutant category. The mean of the prediction probability associated with the 11 mispredicted samples with p. R273H mutation (excluding TCGA-AR-A24T-01 sample) was 0.67 (±0.12 sd) which is considerably lower than the prediction probability of TCGA-AR-A24T (0.96). To determine if this is related to clonality or tumor content, we looked at the variant allele frequency (VAF) of all the samples containing a p. R273H mutation. The average VAF of the 89 correctly classified samples was 0.56 (±0.20 sd) with a median of 0.55 (IQR: 0.39–0.71) while the average VAF of the 11 misclassified samples was 0.31 (±0.16 sd) with a median of 0.26 (IQR: 0.19–0.44). The VAF of TCGA-AR-A24T-01 was approximately 0.30 which is closer to the mean and median of the mispredicted samples. Considering the low VAF, it is possible that low tumor content in this sample might account for the incorrect prediction.

### Samples with non-impactful *TP53* mutations

The samples with non-impactful mutations were excluded from all the previous analyses due to ambiguity in their pathogenicity. To discern the effect of non-impactful mutations, the algorithm was trained on the merged set, and the mutational status of the samples with non-impactful *TP53* mutations was determined by the fully trained RF (Supplementary Table S7). In most mutation groups, many of the samples were assigned to the wild-type category as expected, whereas 30 out of 38 (80%) of the silent mutations were categorized as p53 mutant. All these silent mutations have been previously reported in patients with cancer, Li-Fraumeni syndrome, or other conditions related to cancer based on the NCBI ClinVar database (Table 1) (Landrum et al., 2018). The c.375G>T, c.375G>A, and c.375G>C (p.T125T) mutations occur at the last nucleotide of exon 4 and were shown to disrupt the *TP53* mRNA splicing either through molecular studies or splice site predictive tools (NM_000546, 2379; NM_000546, 1778; NM_000546a). The c.672G>A (p.E224E) mutation occurs at the last nucleotide of exon 5 and was shown to lead to aberrant mRNA splicing (NM_000546b). The c.993G>A (p.Q331Q) mutation is located at the last nucleotide of exon 8 and is predicted to affect the normal mRNA splicing (NM_000546, 2886). Supek et al. have also demonstrated that p. T125T, p. E224E, and p. Q331Q mutations are enriched in *TP53* and suggested that they have a functional role in cancer (Supek et al., 2014).

**TABLE 1:** Silent mutations classified as p53 mutant, the number of samples containing these mutations, and the reported consequences and interpretation of them based on ClinVar database (nucleotide variations with * are not present in general population based on ClinVar evidence).

Nucleotide variation	Amino acid variation	Number of samples	Exon location	Disease	ClinVar pathogenicity	ClinVar record
c.375G>T*	p.T125T	20	Last nucleotide of exon 4	Li-Fraumeni syndrome	Likely pathogenic	VCV000237948.3 (NM_000546, 2379)
c.375G>A*	p.T125T	3	Last nucleotide of exon 4	Li-Fraumeni syndrome	Pathogenic	VCV000177825.18 (NM_000546, 1778)
				Li-Fraumeni-like/Chompret criteria		
				Rhabdomyosarcoma		
				Breast and/or ovarian cancer		
				Malignant tumour of prostate		
c.375G>C	p.T125T	1	Last nucleotide of exon 4	Ependymoma	Likely pathogenic	VCV000480746.3 (NM_000546a)
				Early-onset breast cancer		
c.672G>A*	p.E224E	3	Last nucleotide of exon 5	Li-Fraumeni syndrome	Pathogenic/Likely pathogenic	VCV000080709.6 (NM_000546b)
				Chompret criteria		
c.993G>A*	p.Q331Q	2	Last nucleotide of exon 8	Adrenocortical carcinoma	Likely pathogenic	VCV000428868.7 (NM_000546, 2886)
				Suspected Li-Fraumeni syndrome		
c.207T>C	p.A69A	1	Exon 4	Li-Fraumeni syndrome	Likely benign	VCV000219841.7 (NM_000546, 2198)
				Hereditary cancer-predisposing syndromes		

**FIGURE 3:**
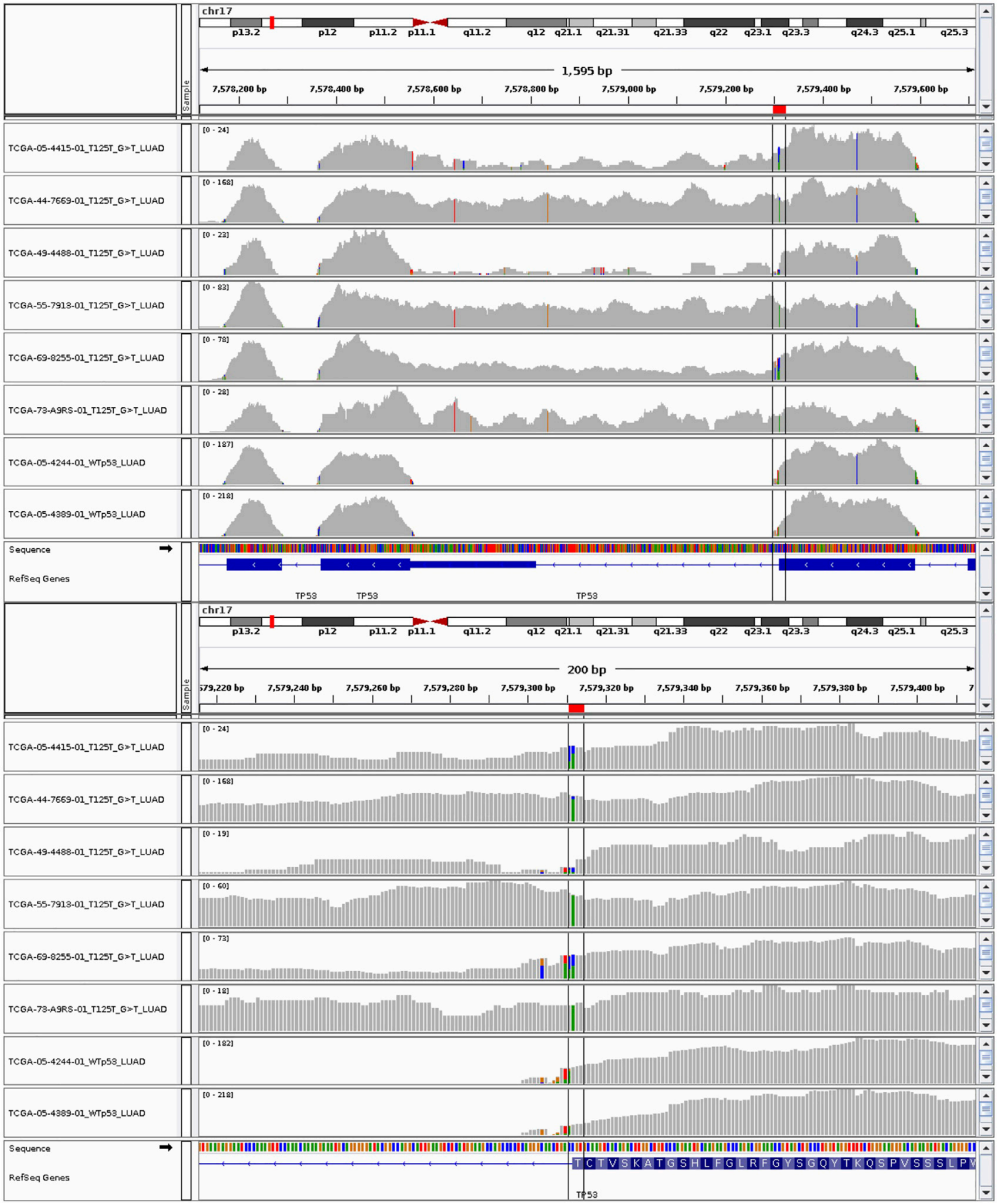
RNA-seq data of lung adenocarcinoma (LUAD) samples with p53 silent mutations at threonine 125 with specific nucleotide modification of G>T. The last two tracks are from LUAD samples with wild-type p53 copies.

The RNA-seq data confirms that the silent mutations which occur at the end of exons (p.T125T, p. E224E, and p. Q331Q) affect the mRNA splicing in these samples (Figure 3 and Supplementary Figure S11). Introns 4, 5, and 8 were not successfully spliced out in samples bearing p. T125T, p. E224E, and p. Q331Q mutations respectively. The only exception to the association between these silent mutations and intron retention was for TCGA-CR-7401-01 where the p. E224E mutation did not appear in the RNA-seq data and splicing appeared normal (Supplementary Figure S11C). With a VAF of 0.14, it may likely be subclonal and the expression not detected. The RNA-seq data of the sample with p. A69A appeared indistinguishable from the other lung squamous cell carcinoma sample with wild-type p53 (Supplementary Figure S11E). This is consistent with its classification in ClinVar as likely benign (NM_000546, 2198). Further investigation is needed to understand why this sample was assigned to the p53 mutant category by the algorithm.

### Treatment efficacy in patients with mutated and wild-type p53

**FIGURE 4:**
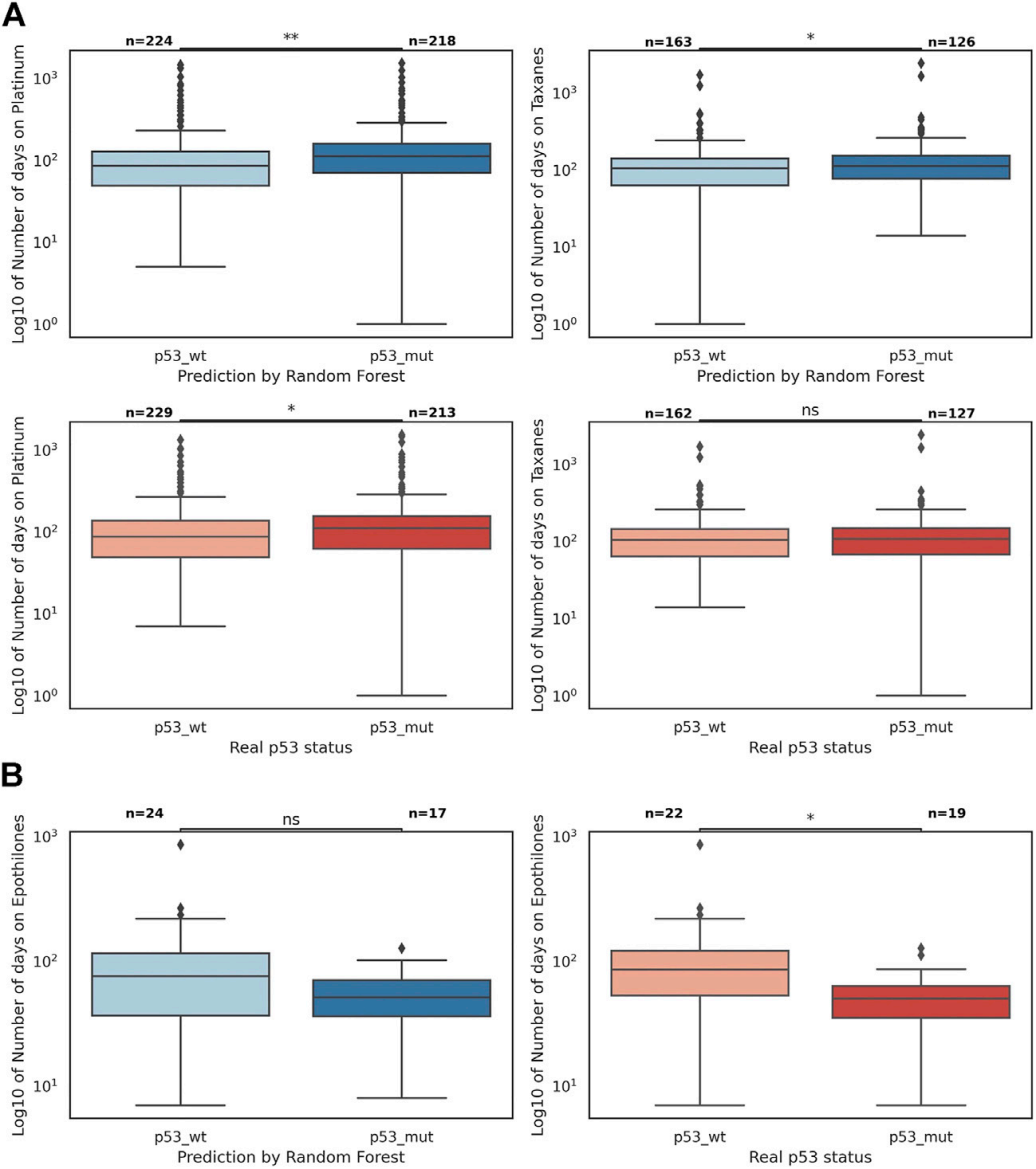
The number of days on platinum therapies, taxanes, and epothilones divided by *TP53* mutation status and the predicted status by the random forest (the *p*-values are found in a Mann-Whitney-Wilcoxon two-sided test with Bonferroni correction; *p*-value annotation guide: ns: 5.00e-02 < p ≤ 1.00, *: 1.00e-02 < p ≤ 5.00e-2, **: 1.00e-03 < p ≤ 1.00e-02). **(A)** The boxplots of log10 of the number of days on platinum and taxanes; the difference between p53 wild-type and mutant sets is statistically more significant when data points are divided by the random forest predictions (blue) than when they are divided by the true p53 status (red). **(B)** The boxplots of log10 of the number of days on epothilones (represented only by the drug eribulin); the difference between p53 wild-type and mutant sets is statistically more significant when data points are divided by the true p53 status (red) than when they are divided by the random forest predictions (blue).

We sought to explore whether *TP53* mutation status was predictive of therapy response for patients within the POG cohort for 29 drug groups (Supplementary Table S8). A longer time on therapy was interpreted to be indicative of ongoing clinical benefit for the patients (Pleasance et al., 2020). Time on therapy was more strongly associated with *TP53* status predicted by the RF than with recorded *TP53* mutation status for platinum therapies (Bonferroni-adjusted pvalue 0.001 vs. 0.027) and taxanes (0.041 vs. 0.288), with longer duration in predicted *TP53*-mutant cases (Figure 4). The majority of these therapies (94% of platinums and 77% of taxanes) were received in combination with other drugs. The reverse association is true for the drug group Epothilones (represented only by the drug eribulin), where the recorded *TP53* mutation status was more strongly associated with time on therapy (Bonferroni-adjusted *p*-value 0.138 vs. 0.014), with longer treatment duration in predicted *TP53*-wild-type cases (Figure 4). For the remaining drug categories, the classification of data points by the recorded *TP53* status and the RF predictions were statistically similar (Supplementary Figure S12).

## Discussion

We developed a RF model derived from tumor transcriptomes which can successfully classify primary and metastatic tumor samples based on *TP53* aberrations. We hypothesize that a specific transcriptional signature is associated with the loss of functional p53 within mutated tumors regardless of their type or tissue of origin since the algorithm stratified 88% of the merged set samples correctly, and the combination of different cancer types as well as the inclusion of both primary and metastatic sets during training enhanced the performance of the model. The TCGA primary and POG metastatic sets were combined for training to discover potential differences in p53 biology and the cellular pathways associated with this transcription factor between the two sets, however, the results from the merged set showed that the p53 inactivation signature is universal across all tumors. We used this p53 transcriptional signature to identify the genes with core roles in p53 processes, determine the functional relevance of silent mutations, and better predict response to treatment.

The choice of the RF algorithm for this work is due to its ability to find complicated patterns in data and improve classification with less overfitting compared to other models (Breiman, 2001; Khoshgoftaar et al., 2013; Maurya et al., 2021). RFs have also been shown to be robust to noise and perform better on imbalanced data sets (Breiman, 2001; Khoshgoftaar et al., 2007; Khoshgoftaar et al., 2013). Even when including highly imbalanced cohorts, the RF model had better performance metrics than the published XGBoost model used to classify p53 pathway activity (Zhang et al., 2021). The RF could additionally identify samples with germline *TP53* mutations and samples with silent mutations that affect the mRNA splicing. These findings confirm the existence of a p53 transcriptional signature and the power of the RF algorithm to detect this signature. The RF, combined with drug treatment data, revealed that the presence of the mutant *TP53* signature was associated with a longer time on therapy for platinum and taxane therapies. It is important to highlight that most patients in the POG cohort received combination therapies, and treatment effectiveness has been shown to be affected by the mode of therapy (Murray and Mirzayans, 2020; Shu et al., 2022). Moreover, our treatment data set was relatively small, so further work will be needed to confirm the treatment efficacy results presented here.

The majority of significant genes in the classification found in this work have known links with p53 function. Based on this strong association, we speculate that many of the other significant transcripts possess an unappreciated role in p53 biology; these include protein coding genes GPSM2, OR4N2, CTSL2, SPERT, and RPE65; pseudogenes RP11-611O2.3, OR4K6P and AC004967.8; long non-coding RNAs (lncRNAs) RP11-611O2.5, LINC00511, AC068057.1, RP11-385J1.2, and RP11-305N23.1. There is existing evidence for roles in tumor biology for many of these genes. GPSM2 has a role in breast cancer cell division and promotes tumor proliferation and metastasis in hepatic cellular cancer (Blumer et al., 2006; Fukukawa et al., 2010; He et al., 2017). Additionally, it has been shown that GPSM2 plays an important role in mitosis (Fukukawa et al., 2010) and our GSEA showed that mitosis and cell division pathways are among the top pathways found to be significant in classification. OR4N2 was shown to be mutated on at least two sites in epithelial ovarian cancers (Zhang et al., 2019). OR4N2 encodes a G protein-coupled receptor and GPSM2 participates in activation of G proteins (Blumer et al., 2006; Maßberg and Hatt, 2018). The p53 signaling pathway, which is the eighth important pathway in our classification, contains several G protein-coupled interactions which highlights the importance of these genes in p53 function (Solyakov et al., 2009). CTSL2 was demonstrated to be highly expressed in various human cancers and was speculated to be associated with metastasis (Santamaría et al., 1998; Liu et al., 2004; Sun et al., 2016). Knockdown of SPERT was shown to lead to tumor growth suppression and apoptosis (Zheng and Chen, 2018). RPE65 was demonstrated to be highly downregulated in melanoma and squamous cell carcinoma of skin (Hinterhuber et al., 2005; Hassel et al., 2013). We also observed that RP11-611O2.3 and RP11-611O2.5 are located at the 3’ end of the MDM2 gene and their low expression in the p53 mutant tumors is consistent with the observed low MDM2 expression in such tumors.

*TP53* functions as a homo-tetramer and the inclusion of mutant protein products provides the mechanism by which p53 mutants can function in a dominant negative manner (Ko and Prives, 1996; de Vries et al., 2002; Donehower et al., 2019; Thomas et al., 2022). For tumor types where the class size was sufficient to allow robust training, over 90% of tumors exhibiting a strong p53 transcriptional signature with a likelihood of >0.75 were found to have a corresponding *TP53* mutation (95, 96, 97, and 91% of tumors respectively for breast invasive carcinoma, colon adenocarcinoma, brain lower grade glioma and lung adenocarcinoma). This indicates that there are no other gene mutations that can generate this signature at a high frequency even though mutations in other genes within the same pathway as *TP53* might have been expected to generate the same DNA repair defect phenotype and transcriptional signature. This further confirms the unique role of p53 as a key contributor to human cancer.

The use of ML in this work led to the discovery of complicated patterns in the transcriptome that otherwise could not be possible to detect. ML approaches were shown to have the ability to detect complex relationships in a fast and accurate manner in many different areas of omics sciences (Wu and Wang, 2018; Smith et al., 2020; Reel et al., 2021). These algorithms are constantly being improved and can be easily automated. Furthermore, RF models have the capability to distinguish different roles that genes might play in different cells by utilizing them at different depths of decision trees. It has been demonstrated that some genes might play different roles based on the cell type or the biological context. For example, it has been postulated that the function of *MELK* might be context dependent and can positively or negatively regulate p53 in different cell types (Seong and Ha, 2012; Gu et al., 2013; Ganguly et al., 2015). RF models can capture such context-dependent relationships since they can use the same gene at different depths of decision trees with different thresholds to split the samples.

In conclusion, we have successfully showed that a RF model can classify tumor samples based on *TP53* status regardless of their type or tissue of origin using expression data alone. The genes contributing to the signature provide insight to p53 biology, and the use of this signature for classification has the potential to aid in treatment management and identification of the patients who can benefit from therapies related to *TP53* status.

## Data Availability

Publicly available datasets were analyzed in this study. This data can be found here: tcga_RSEM_gene_tpm was downloaded from https://xenabrowser.net/on 21 June 2021 and the MC3 Public MAF mutation data (mc3. v0.2.8. PUBLIC.maf.gz) was downloaded from: https://gdc.cancer.gov/about-data/publications/mc3-2017 on 30 September 2020.
