# Supplementary material for: A p53 transcriptional signature in primary and metastatic cancers derived using machine learning
Source: Front Genet. 2022 Aug 29;13:987238. doi: 10.3389/fgene.2022.987238 (PMC9483853; doi:10.3389/fgene.2022.987238)
Supplement: Supplementary file 1 [file DataSheet1.docx]

Supplementary Material

# Supplementary Methods

Expression matrices containing TPM (transcript per million) values and mutation datasets were obtained from the TCGA (tcga_RSEM_gene_tpm was downloaded from [https://xenabrowser.net/](https://xenabrowser.net/3) on June 21, 2021 and the MC3 Public MAF mutation data (mc3.v0.2.8.PUBLIC.maf.gz) was downloaded from: <https://gdc.cancer.gov/about-data/publications/mc3-2017> on September 30, 2020) and POG studies. After excluding the TCGA non-primary tumor samples and the samples lacking mutation data, TCGA and POG expression matrices were consolidated based on a list of comparable genes that overlap between both data sets. In total 48,784 transcribed genes, including *TP53*, were used in this study, of which 18,337 (38%) are annotated as protein coding. The Principal Component Analysis (PCA) plots were made for TCGA and POG expression data sets, both separately and jointly, after log2 (TPM+0.001) values were obtained (Supplementary figure 1). The principal components were found using scikit-learn (version 0.23.2) decomposition PCA function (1) and the plots were created using the seaborn and matplotlib libraries (versions 0.11.0 and 3.3.2) (2,3). Next, samples were divided into two groups based on their mutational status (i.e., mutated vs wildtype). The samples in the mutated group were further divided into “impactful” and “non-impactful” categories based on the expected consequence of the mutations. The samples containing a mutation of type “silent”, “intron”, “3-prime UTR”, “5-prime UTR”, “downstream gene”, “upstream gene”, and “splice region” were put into “non-impactful” group since no modification is expected in the resulting p53 protein, and the rest of the samples containing a p53 mutation were categorized as “impactful”.

All data analyses performed in this study was carried out using Python 3.8.5 and the available tools in the scikit-learn package 0.23.2 (1).

## Random Forest Performance

For this analysis, only samples with “impactful” mutations or wildtype p53 copies were included and the RF classifier performance was evaluated on the TCGA, POG and merged (set of all impactful and wildtype TCGA and POG samples) data sets. This was to increase the likelihood of only pathogenic *TP53* driver alterations being used for training. To optimize the performance of the algorithm, first the GridSearchCV function of the scikit-learn package was used to find the best values for the main hyperparameters (Supplementary table 1). For each data set, 90% of samples were used to calibrate the hyperparameters in a 5-fold cross-validation (5-fold CV) analysis and the other 10% were used to validate the obtained values. The best accuracy score found in grid search (using 90% of samples) was used to pick the most suitable hyperparameters which also improved the accuracy, precision, recall, and f1-score metrics of the validation set (10% of samples) when compared with the performance using the default hyperparameters.

The tested values were selected by making a graph of mean prediction accuracy across 5-folds versus various values of each hyperparameter using 90% of the merged set samples and picking the values around the point where the mean accuracy plateaus (Supplementary figure 2). In these graphs, the rest of the hyperparameters were kept fixed by using the scikit-learn RF classifier default values. Except for the number of trees, these values were used for the rest of the analyses. The number of trees selected by the GridSearchCV function was at the very end of the tested range and larger number of trees were not tested in this grid search due to extremely long runtimes (Supplementary figure 3). Thus, a further optimization step was to increase the number of trees while fixing the rest of the obtained hyperparameter values. By increasing the number of trees, a higher percentage of genes are found in independent training iterations, however, the algorithm performance is negatively affected when the number of trees is increased to above 3000 (Supplementary figures 4-5). Thus, in all the subsequent analyses, the number of decision trees was set to 3000 and the rest of the hyperparameters were set to the values in supplementary table 1. After identifying suitable hyperparameters, the RF performance was evaluated across TCGA, POG, and merged data sets in a 5-fold CV analysis using all impactful and wildtype samples. The precision, recall, F1-score, area under the precision-recall curve (AUPRC), and area under the receiver operating characteristic curve (AUROC) values are reported in supplementary table 2 and the plots of AUROC and AUPRC can be found in figure 1.

To examine the algorithm performance across 33 TCGA cancer types, precision, recall and f1-score metrics were calculated for each cancer type using the algorithm predictions from the previous analysis (Supplementary table 3). Additionally, to compare the performance on each cancer type individually versus the pan-cancer set, the accuracy scores were compared to the Out of Bag (OOB) scores which were found by training the model using only the samples in each cancer type (Supplementary figure 6). Since OOB scores show the proportion of correct predictions for out of bag samples, they are the closest metrics to the accuracy scores. The benefit of OOB score is that it can be used to measure the performance using all the samples when dealing with small data sets such as those containing only the samples from each cancer type.

## Significant Genes in Classification

The genes that played a more important role in classification were extracted using the built-in feature importance scores of scikit-learn RF model. The threshold for the number of important genes (features) was found based on a permutation method. In this method, first the top 1000 genes with the highest importance scores were extracted after training the model using the samples in the merged set. Then, in 100 independent permutations, a RF algorithm was trained using an expression data set containing the same set of samples and the top 1000 genes as well as a list of randomly shuffled labels. After each iteration, the importance scores of the gene ranks were recorded, and at the end of 100 permutations, the mean and standard deviation of the importance scores per rank were obtained. The RF was also trained using the same filtered expression data set and the list of true labels and a graph of the true importance scores and the mean of importance scores with random labels were made. The threshold was defined as the point where the line of true importance scores crossed the line of the mean of the 100 random permutations scores (Supplementary figure 7). Using this threshold, the top genes and their importance scores were extracted from the RF (Figure 2). The mutation rates of these genes in the primary and metastatic sets as well as the modification in their expression with respect to loss of wildtype p53 function are summarized in supplementary table 4. Furthermore, the association of these genes with p53 was investigated through a thorough literature review (Supplementary table 4).

The boxplots of expression values of the top 10 genes in p53-mutant and wildtype samples were also made to check the coherence of our findings (Supplementary figure 8) and the list of top 67 genes were used to perform a Gene Set Enrichment Analysis (GSEA) using the Database for Annotation, Visualization, and Integration Discovery (DAVID) (Supplementary table 5) (4,5). Finally, to evaluate the importance of *TP53* in classification, the RF was trained 100 times using all the samples and genes, and the rank of p53 was extracted after each iteration. The summary statistics of the p53 ranks were then calculated.

## Prediction Probabilities and Outliers

The probabilities associated with the RF predictions were extracted from the model and (Supplementary figure 9) mis-predicted samples with high prediction probabilities (>0.95) were identified (Supplementary table 6). Two of these samples (TCGA-AR-A24T-01 and TCGA-VM-A8CH) that belonged to relatively balanced cancer cohorts were further investigated. The whole exome sequencing (WXS) and RNA-seq files of these samples and a few other brain and breast cancer samples were visualized using the Broad Institute’s Integrative Genomics Viewer (IGV) to explore potential variations (Supplementary figure 10) (6).

## Samples with Non-Impactful p53 Mutations

The samples with non-impactful mutations, i.e., mutations of type “silent”, “intron”, “3-prime UTR”, “5-prim UTR”, “downstream gene”, “upstream gene”, and “splice region”, were excluded from all the previous analyses due to ambiguity in their pathogenicity. To determine the status of these samples, the merged set was used to train the algorithm, and the status of non-impactful samples were predicted. These predictions were grouped by mutation type and predicted p53 status (Supplementary table 7). The samples with silent mutations that were assigned to p53 mutant category were further investigated since it was expected to see no change in the produced p53 protein and a similar behavior to p53 wildtype category for the samples with these mutations. The specific amino acid and nucleotide modifications of these samples were also acquired and used to group them (Table 1). In addition, the samples RNA-seq data was obtained and visualized in IGV (Figure 3 and supplementary figure 11).

## Treatment Efficacy in Patients with Mutated and Wildtype p53

To inspect the capability of the RF to detect the patients who can benefit from different therapies, the available treatment data for patients in the POG cohort was obtained. The drugs used for these patients were grouped by their mechanism of action and/or target genes or proteins (Supplementary table 8) using different sources including the Synthesis of Best-Seller Drugs book, the LiverTox online book, the DRUGBANK database, and the National Cancer Institute Drug Dictionary (7–10). The number of days in the course of treatment for all patients and drugs was calculated to compare the treatment efficacy in patients with tumors containing mutated or wildtype p53 copies. Time on therapy was used as a proxy for treatment response as response data was not available for these treatments; a long duration of treatment generally indicates ongoing clinical benefit for the patient. In those cases where patients were treated using combination therapies, drugs were separated and the number of days on the combination therapy was used for each of them individually. Additionally, data for patients on a double-blind trial (drug/placebo) where the received treatment was unknown was filtered out. Some drug groups were also removed before the analysis since either there were very few patients receiving them (<5 data points) or all the patients had p53 wildtype tumors. For the rest of the drug categories, the patients were divided by their *TP53* gene status (wildtype vs mutated) and the predicted p53 status by the RF and the boxplots of the log10 of the number of days for these groups were made (Figure 4 and supplementary figure 12).

# Supplementary Figures and Tables

## Supplementary Tables

**Supplementary Table 1.** The hyperparameters tested to optimize the random forest performance, the sets of tested values, and the value with the best mean accuracy score for POG, TCGA, and merged datasets. The values for maximum number of samples per DT and maximum number of features per split are the proportion of the samples and features used in the training set (DT: Decision Tree).

| **Hyperparameter** | **Tested Values** | **Best Value (POG)** | **Best Value (TCGA)** | **Best Value (Merged Set)** |
| --- | --- | --- | --- | --- |
| Number of DTs | [25, 50, 100, 200] | 50 | 200 | 200 |
| Max. Number of Samples per DT | [0.2, 0.4, 0.6, 0.8, 0.99] | 0.99 | 0.99 | 0.99 |
| Max. Number of Features per Split | ['sqrt', 0.01, 0.03, 0.05] | 0.05 | 0.05 | 0.05 |
| Max. Depth of DTs | [10, 50, 100, 200] | 100 | 50 | 50 |
| Min. Number of Samples to Split | [2, 10, 25, 50] | 50 | 25 | 2 |
| Min. Number of Samples in Leaves | [1, 2, 10, 50] | 1 | 2 | 2 |

**Supplementary Table 2.** Overall performance of the random forest across data sets (AUPRC=Area Under the Precision-Recall Curve, AUROC=Area Under the Receiver Operating Characteristic).

|  | **Precision** | **Recall** | **F1-score** | **AUPRC** | **AUROC** |
| --- | --- | --- | --- | --- | --- |
| **TCGA** | 0.88 | 0.88 | 0.88 | 0.96 | 0.94 |
| **POG** | 0.73 | 0.68 | 0.69 | 0.88 | 0.82 |
| **Merged Data** | 0.88 | 0.87 | 0.87 | 0.96 | 0.94 |

**Supplementary Table 3.** Performance of the random forest and the number of samples in each p53 category (impactful mutation vs wild type) across 33 TCGA cancer types (ACC=Adrenocortical carcinoma, BLCA=Bladder Urothelial Carcinoma, BRCA=Breast invasive carcinoma, CESC=Cervical squamous cell carcinoma and endocervical adenocarcinoma, CHOL=Cholangiocarcinoma, COAD=Colon adenocarcinoma, DLBC=Lymphoid Neoplasm Diffuse Large B-cell Lymphoma, ESCA=Esophageal carcinoma, GBM=Glioblastoma multiforme, HNSC=Head and Neck squamous cell carcinoma, KICH=Kidney Chromophobe, KIRC=Kidney renal clear cell carcinoma, KIRP=Kidney renal papillary cell carcinoma, LAML=Acute Myeloid Leukemia, LGG=Brain Lower Grade Glioma, LIHC=Liver hepatocellular carcinoma, LUAD=Lung adenocarcinoma, LUSC=Lung squamous cell carcinoma, MESO=Mesothelioma, OV=Ovarian serous cystadenocarcinoma, PAAD=Pancreatic adenocarcinoma, PCPG=Pheochromocytoma and Paraganglioma, PRAD=Prostate adenocarcinoma, READ=Rectum adenocarcinoma, SARC=Sarcoma, SKCM=Skin Cutaneous Melanoma, STAD=Stomach adenocarcinoma, TGCT=Testicular Germ Cell Tumors, THCA=Thyroid carcinoma, THYM=Thymoma, UCEC=Uterine Corpus Endometrial Carcinoma, UCS=Uterine Carcinosarcoma, UVM=Uveal Melanoma).

| **Cancer Type** | **Num Impactful p53** | **Num Wild type p53** | **Precision** | **Recall** | **F1-score** |
| --- | --- | --- | --- | --- | --- |
| ACC | 15 | 61 | 0.792 | 0.592 | 0.609 |
| BLCA | 197 | 206 | 0.824 | 0.824 | 0.824 |
| BRCA | 345 | 667 | 0.876 | 0.830 | 0.846 |
| CESC | 22 | 263 | 0.649 | 0.691 | 0.666 |
| CHOL | 4 | 32 | 0.985 | 0.875 | 0.921 |
| COAD | 166 | 109 | 0.835 | 0.828 | 0.831 |
| DLBC | 5 | 32 | 0.432 | 0.500 | 0.464 |
| ESCA | 154 | 26 | 0.794 | 0.625 | 0.660 |
| GBM | 51 | 97 | 0.849 | 0.773 | 0.794 |
| HNSC | 351 | 142 | 0.864 | 0.779 | 0.806 |
| KICH | 21 | 44 | 0.855 | 0.571 | 0.540 |
| KIRC | 11 | 355 | 0.989 | 0.636 | 0.709 |
| KIRP | 7 | 273 | 0.487 | 0.498 | 0.493 |
| LAML | 8 | 116 | 0.468 | 0.500 | 0.483 |
| LGG | 242 | 262 | 0.950 | 0.947 | 0.948 |
| LIHC | 108 | 244 | 0.855 | 0.733 | 0.761 |
| LUAD | 258 | 243 | 0.840 | 0.840 | 0.840 |
| LUSC | 400 | 65 | 0.884 | 0.650 | 0.699 |
| MESO | 14 | 68 | 0.930 | 0.607 | 0.639 |
| OV | 265 | 18 | 0.893 | 0.637 | 0.695 |
| PAAD | 103 | 67 | 0.788 | 0.802 | 0.786 |
| PCPG | 1 | 179 | 0.497 | 0.500 | 0.499 |
| PRAD | 58 | 436 | 0.643 | 0.514 | 0.500 |
| READ | 76 | 13 | 0.865 | 0.865 | 0.865 |
| SARC | 84 | 147 | 0.659 | 0.645 | 0.649 |
| SKCM | 9 | 93 | 0.874 | 0.717 | 0.770 |
| STAD | 200 | 208 | 0.808 | 0.805 | 0.804 |
| TGCT | 1 | 149 | 0.497 | 0.500 | 0.498 |
| THCA | 3 | 486 | 0.497 | 0.500 | 0.498 |
| THYM | 3 | 115 | 0.487 | 0.500 | 0.494 |
| UCEC | 92 | 82 | 0.845 | 0.846 | 0.845 |
| UCS | 52 | 5 | 0.981 | 0.800 | 0.866 |
| UVM | 0 | 79 | 1.000 | 1.000 | 1.000 |

**Supplementary Table 4.** List of top genes, their importance scores, mutation rates across primary (TCGA) and metastatic (POG) samples, modification in expression in presence of p53 mutations, and link to p53

| **Rank** | **Gene** | **Importance Score** | **Mutation Rate in TCGA** | **Mutation Rate in POG** | **Up- or Down- Regulated with Mutated p53** | **Link to p53** | **Ref** |
| --- | --- | --- | --- | --- | --- | --- | --- |
| 1 | EDA2R | 0.03104 | 0.97% | 16.84% | Down | Is a direct target of p53 that mediates p53-induced anoikis pathway | (11,12) |
| 2 | RPS27L | 0.02130 | 0.36% | 3.86% | Down | Is a direct p53 target that binds to MDM2 and inhibits p53 degradation (regulates p53 through MDM2) | (13) |
| 3 | MDM2 | 0.02035 | 1.00% | 7.89% | Down | Is a negative regulator of p53 and its direct target (is activated by p53) | (14,15) |
| 4 | MYBL2 | 0.00884 | 1.76% | 12.46% | Up | p53-p21-DREAM pathway can repress MYBL2 expression and MYBL2 increases STRAP-mediated stimulation of p53 | (16,17) |
| 5 | UBE2C | 0.00841 | 0.71% | 1.40% | Up | Is repressed by wildtype p53 while GOF mutant p53 can activate it | (18) |
| 6 | CDC20 | 0.00832 | 0.96% | 2.28% | Up | Is negatively regulated by p53 (directly or indirectly) | (19,20) |
| 7 | RP11-115D19.1 | 0.00769 | _ | 34.21% | Down | A lncRNA; its expression is negatively associated with TP53 mutations | (21) |
| 8 | FAM83D | 0.00761 | 1.34% | 9.47% | Up | Its expression is positively associated with TP53 mutations | (22) |
| 9 | RP11-611O2.5 (ENSG00000257181) | 0.00748 | _ | 1.40% | Down | LncRNA (is located opposite of the 3’ end of MDM2 on minus strand of chr12 q15) |  |
| 10 | DDB2 | 0.00632 | 0.92% | 5.26% | Down | Directly regulates p53 levels and is directly induced by p53 | (23) |
| 11 | CDCA5 | 0.00598 | 0.60% | 5.61% | Up | No direct link, but CDCA5 is significantly correlated with key cell cycle regulating proteins in p53 signalling pathway including CDK1, CDK2, CCNB1, etc. | (24,25) |
| 12 | CENPA | 0.00533 | 0.31% | 5.79% | Up | Is negatively regulated by p53 through p21; its overexpression changes the cell fate depending on p53 status | (26,27) |
| 13 | ANLN | 0.00517 | 2.18% | 13.86% | Up | Is a direct target of p53 and is repressed by wildtype p53 | (28,29) |
| 14 | AEN | 0.00506 | 0.77% | 4.04% | Down | Is a direct target of p53 and its expression is dependent on p53 phosphorylation status | (30) |
| 15 | CDCA8 | 0.00504 | 0.68% | 5.44% | Up | Its overexpression is highly associated with p53 mutations and is repressed in presence of p53 | (31,32) |
| 16 | PLK1 | 0.00487 | 1.57% | 3.33% | Up | Physically binds to p53 and inhibits p53 function | (33) |
| 17 | SPATA18 | 0.00477 | 1.89% | 14.39% | Down | Is a direct target of p53 and is activated by p53 in various cell types | (34) |
| 18 | RP11-611O2.3 (ENSG00000256664) | 0.00473 | _ | 0.70% | Down | pseudogene (is located at the 3’ end of MDM2 on plus strand of chr12 q15) |  |
| 19 | TICRR | 0.00472 | 2.74% | 8.25% | Up | In TICRR depleted cells, p53 signalling pathway function and p53 expression is elevated | (35) |
| 20 | KIF2C | 0.00458 | 1.57% | 6.67% | Up | Is negatively regulated by p53 and is a p53–DREAM pathway target | (36,37) |
| 21 | CENPW | 0.00399 | 0.27% | 2.46% | Up | No known direct link to p53, but it activates STAT1 and STAT1 is a coactivator of p53 and negative regulator of MDM2 | (38,39) |
| 22 | CEP55 | 0.00394 | 0.89% | 6.84% | Up | Is negatively regulated by p53 through PLK1 | (40) |
| 23 | CIRBP | 0.00381 | 1.09% | 0.88% | Down | It negatively regulates p53 levels | (41) |
| 24 | TPX2 | 0.00378 | 1.46% | 13.68% | Up | Its silencing increases p53 expression through either direct interaction with p53 or MDM2; it might also be a target of p53 | (42,43) |
| 25 | FDXR | 0.00376 | 1.22% | 4.74% | Down | Is a direct target of p53 and is induced by wildtype p53 | (44) |
| 26 | CDKN2A | 0.00370 | 4.30% | 5.79% | Up | p19^ARF^ transcript of CDKN2A promotes MDM2 degradation and thus, p53 stabilization | (45,46) |
| 27 | AUNIP (AIBP) | 0.00346 | 0.41% | 5.09% | Up | No direct link to p53, but it interacts with AURKA and partially controls the localization, phosphorylation, and activation of both AURKA and PLK1 (both genes exist in this table); in addition, it plays a role in HRR pathway | (47–49) |
| 28 | TTK | 0.00336 | 2.38% | 10.35% | Up | Phosphorylates p53 at Thr18 that leads to p53 stabilization | (50) |
| 29 | PRR11 | 0.00336 | 0.77% | 9.65% | Up | Its expression is negatively regulated by wild type p53 | (51,52) |
| 30 | FAM54A | 0.00327 | _ | 5.96% | Up | Its expression level is correlated with p53 mutation status | (53) |
| 31 | PTCHD4 | 0.00317 | 2.90% | 44.91% | Down | Is a direct target of p53 and is extremely lowly expressed in p53 mutated samples across diverse tumour types | (54) |
| 32 | ALG3 | 0.00314 | 1.11% | 2.98% | Up | Its expression increases considerably with p53 mutations | (55) |
| 33 | GPSM2 | 0.00306 | 1.26% | 8.77% | Up | Not known |  |
| 34 | MELK | 0.00305 | 1.19% | 21.40% | Up | Directly interacts with p53 and induces its expression in breast and colon cancer cell lines, while in glioma stem cells, lack of MELK induces p53 expression; in addition, wildtype p53 was shown to negatively regulate MELK expression | (56–59) |
| 35 | OR4K6P | 0.00300 | - | 6.67% | Up | Not known |  |
| 36 | XPC | 0.00292 | 1.26% | 4.91% | Down | Is positively regulated by p53; it has also been shown that XPC negatively regulates p53 | (60–63) |
| 37 | TROAP | 0.00291 | 1.73% | 3.51% | Up | Is regulated by p53–DREAM pathway | (64) |
| 38 | BIRC5 | 0.00288 | 0.52% | 5.26% | Up | Is negatively regulated by wild type 53 at both mRNA and protein levels | (65) |
| 39 | AC004967.8 (ENSG00000183444) | 0.00286 | _ | 1.05% | Up | Not known |  |
| 40 | TRIP13 | 0.00285 | 0.99% | 8.60% | Up | Negatively regulates p53 levels | (66,67) |
| 41 | OR4N2 | 0.00273 | 2.71% | 25.79% | Up | Not known |  |
| 42 | CCNG1 | 0.00240 | 0.52% | 2.63% | Down | Is a direct target of p53 while it negatively regulates p53 via MDM2 activation | (68,69) |
| 43 | CTSL2 | 0.00233 | - | 4.91% | Up | Not known |  |
| 44 | LINC00511 | 0.00217 | - | 40.00% | Up | Not known |  |
| 45 | SPERT | 0.00216 | 1.52% | 6.84% | Up | Not known |  |
| 46 | AC068057.1 | 0.00212 | - | 8.42% | Down | Not known |  |
| 47 | RP11-385J1.2 | 0.00204 | _ | 36.32% | Up | Not known |  |
| 48 | FUT3 | 0.00198 | 1.32% | 4.39% | Up | It is upregulated in samples with mutated p53 and downregulated in samples with wildtype p53 | (70) |
| 49 | CCNB2 | 0.00190 | 0.59% | 3.68% | Up | Is negatively and indirectly regulated by p53 | (71) |
| 50 | AURKA | 0.00187 | 0.70% | 5.44% | Up | It can lead to inactivation of p53 or its degradation by MDM2; in addition, loss of p53 leads to overexpression of this gene via FBXW7 down-regulation | (72–75) |
| 51 | NUF2 | 0.00185 | 1.25% | 9.65% | Up | Is transcriptionally activated by hnRNP K which is negatively regulated by MDM2 and is a transcriptional coactivator of p53 (although does not affect p53 stabilization) | (76,77) |
| 52 | RP11-346D19.1 | 0.00180 | _ | 7.19% | Up | LncRNA; Is found at higher levels in TP53 mutant tumours | (78) |
| 53 | PCM1 | 0.00169 | 2.38% | 15.09% | Down | Lack of PCM1 leads to activation of p53-dependent checkpoint and cell cycle exit | (79) |
| 54 | DLGAP5 | 0.00169 | 1.46% | 8.95% | Up | Lack of DLGAP5 leads to increased p53 protein levels and p53 pathway activation while overexpression of this gene promotes p53 degradation | (80–82) |
| 55 | PHLDA3 | 0.00169 | 0.50% | 2.28% | Down | Is a direct target of p53 and is induced by wildtype p53 | (83) |
| 56 | HJURP | 0.00163 | 1.53% | 5.79% | Up | Is negatively regulated by p53 while it positively regulates MDM2 expression | (26,84) |
| 57 | CDCA3 | 0.00155 | 0.68% | 1.05% | Up | Overexpression of these two genes lead to downregulation of cell cycle checkpoints such as CDK2 and CDKN1A; also, overexpression of CDCA3 leads to p53 downregulation | (85) |
| 58 | KIF18B | 0.00154 | 1.69% | 5.26% | Up |  |  |
| 59 | AURKB | 0.00153 | 0.73% | 3.51% | Up | Phosphorylates p53 and accelerates its degradation and thus, inhibition of this gene leads to p53 accumulation | (86–88) |
| 60 | SLC2A12 | 0.00146 | 1.43% | 12.28% | Up | Is a direct target of p53 and is negatively regulated by it | (89) |
| 61 | DRG2 | 0.00140 | 0.78% | 6.84% | Down | Negatively regulates SIRT1 which deacetylates p53 (downregulation of SIRT1 leads to p53 activation) | (90,91) |
| 62 | RP11-305N23.1 | 0.00136 | _ | 22.28% | Up | Not known |  |
| 63 | BBC3 | 0.00136 | 0.44% | 5.09% | Down | Is a direct target of p53 and is induced by it | (92–94) |
| 64 | RP3-510D11.2 | 0.00133 | _ | 0.70% | Down | LncRNA; Is a direct target of p53 and is induced by it | (95,96) |
| 65 | CDKN1A (p21/WAF1) | 0.00131 | 1.19% | 2.63% | Down | Is a direct target of p53 and is the first gene known to be upregulated by p53; it has also been shown that p53/47 isoform suppresses p21 under activation of unfolded protein response | (97,98) |
| 66 | BAX | 0.00129 | 0.75% | 3.51% | Down | Is a direct target of p53 and is induced by it | (99) |
| 67 | RPE65 | 0.00124 | 1.71% | 9.47% | Up | Not known |  |

**Supplementary Table 5.** Cellular pathways related to the list of important genes for classification found using DAVID (Adjusted p-values are found via Benjamini-Hochberg procedure)

| **Category** | **Pathway** | **Gene count** | **P-value** | **Adjusted p-value** |
| --- | --- | --- | --- | --- |
| UP_KEYWORDS | Cell cycle | 26 | 7.4e-24 | 8.9e-22 |
| UP_KEYWORDS | Mitosis | 20 | 4.0e-23 | 2.4e-21 |
| UP_KEYWORDS | Cell division | 20 | 7.1e-20 | 2.8e-18 |
| GOTERM_BP_DIRECT | Mitotic nuclear division | 16 | 4.4e-16 | 2.1e-13 |
| GOTERM_BP_DIRECT | Cell division | 15 | 1.5e-12 | 3.5e-10 |
| UP_KEYWORDS | Ubl conjugation | 23 | 5.2e-11 | 1.6e-9 |
| GOTERM_CC_DIRECT | Chromosome, centromeric region | 8 | 2.8e-10 | 3.0e-8 |
| KEGG_PATHWAY | P53 signaling pathway | 8 | 6.3e-10 | 3.0e-8 |
| UP_KEYWORDS | Centromere | 9 | 2.2e-9 | 5.2e-8 |
| GOTERM_BP_DIRECT | Sister chromatid cohesion | 9 | 7.8e-10 | 1.2e-7 |
| UP_KEYWORDS | Cytoskeleton | 16 | 1.1e-7 | 2.2e-6 |
| GOTERM_CC_DIRECT | Nucleoplasm | 26 | 4.1e-8 | 2.2e-6 |
| UP_KEYWORDS | Phosphoprotein | 41 | 1.7e-7 | 2.9e-6 |
| UP_KEYWORDS | Cytoplasm | 31 | 2.0e-7 | 3.0e-6 |
| GOTERM_CC_DIRECT | Chromosome passenger complex | 4 | 2.3e-7 | 8.3e-6 |
| UP_KEYWORDS | Chromosome | 10 | 7.5e-7 | 1.0e-5 |
| GOTERM_BP_DIRECT | G2/M transition of mitotic cell cycle | 8 | 1.8e-7 | 2.1e-5 |
| GOTERM_MF_DIRECT | Protein binding | 45 | 2.6e-7 | 3.2e-5 |
| KEGG_PATHWAY | Cell cycle | 7 | 1.3e-6 | 3.2e-5 |
| GOTERM_CC_DIRECT | Spindle | 7 | 1.3e-6 | 3.6e-5 |
| UP_KEYWORDS | Kinetochore | 6 | 5.6e-6 | 6.7e-5 |
| GOTERM_CC_DIRECT | Spindle midzone | 4 | 2.2e-5 | 4.7e-4 |
| GOTERM_CC_DIRECT | Midbody | 6 | 3.6e-5 | 6.5e-4 |
| UP_SEQ_FEATURE | Mutagenesis site | 19 | 6.8e-6 | 1.2e-3 |
| GOTERM_BP_DIRECT | Spindle organization | 4 | 1.4e-5 | 1.4e-3 |
| GOTERM_CC_DIRECT | Kinetochore | 5 | 9.0e-5 | 1.4e-3 |
| GOTERM_CC_DIRECT | Condensed chromosome kinetochore | 5 | 1.2e-4 | 1.6e-3 |
| GOTERM_BP_DIRECT | Protein sumoylation | 6 | 2.7e-5 | 2.2e-3 |
| GOTERM_CC_DIRECT | Spindle microtubule | 4 | 2.8e-4 | 3.1e-3 |
| GOTERM_CC_DIRECT | Spindle pole | 5 | 2.8e-4 | 3.1e-3 |
| GOTERM_CC_DIRECT | Spindle pole centrosome | 3 | 3.7e-4 | 3.3e-3 |
| GOTERM_CC_DIRECT | Condensed nuclear chromosome, centromeric region | 3 | 3.7e-4 | 3.3e-3 |
| UP_KEYWORDS | Apoptosis | 8 | 4.3e-4 | 4.7e-3 |
| GOTERM_BP_DIRECT | Mitotic cytokinesis | 4 | 9.1e-5 | 5.3e-3 |
| GOTERM_BP_DIRECT | Anaphase-promoting complex-dependent catabolic process | 5 | 9.6e-5 | 5.3e-3 |
| GOTERM_BP_DIRECT | Protein ubiquitination involved in ubiquitin-dependent protein catabolic process | 6 | 9.9e-5 | 5.3e-3 |
| GOTERM_BP_DIRECT | Intrinsic apoptotic signaling pathway in response to DNA damage by p53 class mediator | 4 | 1.1e-4 | 5.4e-3 |
| GOTERM_CC_DIRECT | Microtubule cytoskeleton | 5 | 6.7e-4 | 5.6e-3 |
| GOTERM_CC_DIRECT | Nucleolus | 10 | 7.4e-4 | 5.7e-3 |
| GOTERM_BP_DIRECT | Positive regulation of exit from mitosis | 3 | 1.3e-4 | 5.9e-3 |
| GOTERM_CC_DIRECT | Nucleus | 28 | 9.4e-4 | 6.8e-3 |
| GOTERM_BP_DIRECT | Mitotic metaphase plate congression | 4 | 1.9e-4 | 7.6e-3 |
| GOTERM_CC_DIRECT | Protein complex | 7 | 1.2e-3 | 8.1e-3 |
| GOTERM_CC_DIRECT | Centrosome | 7 | 1.4e-3 | 9.1e-3 |
| GOTERM_CC_DIRECT | Cytosol | 20 | 1.7e-3 | 1.0e-2 |
| GOTERM_BP_DIRECT | Positive regulation of ubiquitin protein ligase activity | 3 | 3.2e-4 | 1.2e-2 |
| UP_KEYWORDS | Nucleus | 25 | 1.4e-3 | 1.4e-2 |
| GOTERM_BP_DIRECT | Regulation of cell cycle | 5 | 5.4e-4 | 1.9e-2 |
| GOTERM_CC_DIRECT | Centriole | 4 | 4.3e-3 | 2.5e-2 |
| GOTERM_BP_DIRECT | Cell proliferation | 7 | 8.1e-4 | 2.6e-2 |
| GOTERM_BP_DIRECT | DNA damage response, signal transduction by p53 class mediator resulting in cell cycle arrest | 4 | 8.8e-4 | 2.6e-2 |
| GOTERM_CC_DIRECT | Intercellular bridge | 3 | 7.3e-3 | 3.9e-2 |
| BIOCARTA | P53 signaling pathway | 3 | 2.8e-3 | 4.3e-2 |
| BIOCARTA | Cell cycle | 3 | 6.0e-3 | 4.3e-2 |
| BIOCARTA | Hypoxia and p53 in the cardiovascular system | 3 | 6.5e-3 | 4.3e-2 |
| UP_KEYWORDS | Cyclin | 3 | 4.8e-3 | 4.4e-2 |
| KEGG_PATHWAY | Viral carcinogenesis | 5 | 3.1e-3 | 4.9e-2 |
| UP_KEYWORDS | Microtubule | 5 | 5.8e-3 | 5.0e-2 |

**Supplementary Table 6.** Mispredicted samples with high prediction probabilities (>0.95)

| **Sample Id** | **p53 Status** | **Prediction** | **Prediction Probability** | **Cancer Type** |
| --- | --- | --- | --- | --- |
| TCGA-CZ-4865-01 | p53_mut | p53_wt | 0.995 | Kidney renal clear cell carcinoma |
| TCGA-EL-A3D6-01 | p53_mut | p53_wt | 0.985 | Thyroid carcinoma |
| TCGA-EM-A1YB-01 | p53_mut | p53_wt | 0.976 | Thyroid carcinoma |
| TCGA-G9-7510-01 | p53_mut | p53_wt | 0.976 | Prostate adenocarcinoma |
| TCGA-CZ-5463-01 | p53_mut | p53_wt | 0.975 | Kidney renal clear cell carcinoma |
| TCGA-BQ-7049-01 | p53_mut | p53_wt | 0.971 | Kidney renal papillary cell carcinoma |
| TCGA-G9-6499-01 | p53_mut | p53_wt | 0.968 | Prostate adenocarcinoma |
| TCGA-YL-A9WX-01 | p53_mut | p53_wt | 0.965 | Prostate adenocarcinoma |
| TCGA-HC-8264-01 | p53_mut | p53_wt | 0.964 | Prostate adenocarcinoma |
| TCGA-AR-A24T-01 | p53_mut | p53_wt | 0.962 | Breast invasive carcinoma |
| TCGA-B1-A655-01 | p53_mut | p53_wt | 0.962 | Kidney renal papillary cell carcinoma |
| TCGA-V1-A8MJ-01 | p53_mut | p53_wt | 0.957 | Prostate adenocarcinoma |
| TCGA-VM-A8CH-01 | p53_wt | p53_mut | 0.953 | Brain lower grade glioma |
| TCGA-BQ-5881-01 | p53_mut | p53_wt | 0.950 | Kidney renal papillary cell carcinoma |

**Supplementary Table 7.** Non-impactful mutations and the number of samples containing them assigned to wildtype and mutant p53 categories (the p-value for each mutation type was obtained via a binomial test and it represents the probability of having the specific number of samples assigned to the p53 mutant group assuming there is a 50% chance that samples will be assigned to this group by random assignment).

| **Mutation Type** | **Number of Samples Classified as WT p53** | **Number of Samples Classified as MUT p53** | **P-value** |
| --- | --- | --- | --- |
| Synonymous (silent) variant | 8 | 30 | 0.0005 |
| Intron variant | 10 | 4 | 0.180 |
| Downstream gene variant | 4 | 1 | 0.375 |
| Upstream gene variant | 3 | 0 | 0.25 |
| 3-prime UTR variant | 2 | 1 | 1.00 |
| 5-prime UTR variant | 1 | 1 | 1.00 |
| Splice region variant | 0 | 1 | 1.00 |
| Splice region variant + intron variant | 2 | 1 | 1.00 |
| Downstream gene variant + 5-prime UTR variant | 0 | 1 | 1.00 |

**Supplementary Table 8.** List of the drug groups used in the treatment efficacy analysis and drugs they contain (PKI=Protein Kinase Inhibitor, MA=Monoclonal Antibodies)

| **Drug Group** | **Drugs** |
| --- | --- |
| ALK Inhibitors (PKIs) | ALECTINIB, BRIGATINIB, CERITINIB, CRIZOTINIB, LORLATINIB |
| Alkylating Agents | BENDAMUSTINE, BUSULFAN, CARMUSTINE, CYCLOPHOSPHAMIDE, DACARBAZINE, IFOSFAMIDE, LOMUSTINE, MELPHALAN, PROCARBAZINE, TEMOZOLOMIDE, TRABECTEDIN |
| Anti-CTLA-4 (MAs) | IPILIMUMAB, TREMELIMUMAB |
| Anti-EGFR (PKIs or MAs) | AFATINIB, CETUXIMAB, ERLOTINIB, GEFITINIB, OSIMERTINIB, PANITUMUMAB, ROCILETINIB |
| Anti-HER2 (PKIs or MAs) | LAPATINIB, PERTUZUMAB, TRASTUZUMAB |
| Anti-PD-1/PD-L1 (MAs) | ATEZOLIZUMAB, AVELUMAB, DURVALUMAB, NIVOLUMAB, PEMBROLIZUMAB |
| Anti-VEGF (PKIs or MAs) | BEVACIZUMAB, CEDIRANIB, RAMUCIRUMAB |
| Antiandrogens | ABIRATERONE, BICALUTAMIDE, ENZALUTAMIDE, FLUTAMIDE |
| Antiestrogens | ANASTROZOLE, EXEMESTANE, FULVESTRANT, LETROZOLE, TAMOXIFEN |
| Antihyperglycemic Agents | METFORMIN |
| BRAF Inhibitors (PKIs) | DABRAFENIB, VEMURAFENIB |
| Bisphosphonate | CLODRONATE, PAMIDRONATE, ZOLEDRONIC ACID |
| CDK4/6 Inhibitors (PKIs) | PALBOCICLIB, RIBOCICLIB |
| Corticosteroids | CORTISONE, DEXAMETHASONE, FLUDROCORTISONE, HYDROCORTISONE, PREDNISONE |
| Cytotoxic Antibiotics | BLEOMYCIN, DACTINOMYCIN, DOXORUBICIN, EPIRUBICIN, MITOMYCIN |
| Epothilones | ERIBULIN |
| Folate Antagonists | LEUCOVORIN, METHOTREXATE, PEMETREXED, RALTITREXED |
| GnRH Analogues | BUSERELIN, DEGARELIX, GOSERELIN, LEUPROLIDE |
| IDO1 inhibitor | BMS-986205 |
| mTOR Inhibitors (PKIs) | EVEROLIMUS, TEMSIROLIMUS |
| Multikinase Inhibitors | AXITINIB, BAY 73-4506, CABOZANTINIB, DOVITINIB, IMATINIB, LENVATINIB, NERATINIB, PAZOPANIB, REGORAFENIB, SORAFENIB, SUNITINIB, VANDETANIB |
| Other Monoclonal Antibodies | AGS67E, ALEMTUZUMAB, CEMIPLIMAB, HERCEPTIN, MONALIZUMAB, MOXR0916, OLARATUMAB, RITUXIMAB |
| PARP inhibitors (PKIs) | NIRAPARIB, OLAPARIB, RUCAPARIB, TALAZOPARIB, VELIPARIB |
| Platinum | CARBOPLATIN, CISPLATIN, OXALIPLATIN |
| Progestins | MEDROXYPROGESTERONE, MEGESTROL |
| Pyrimidine Analogues | CAPECITABINE, CYTARABINE, FLUOROURACIL, GEMCITABINE, LONSURF |
| Taxanes | DOCETAXEL, GENETAXYL, PACLITAXEL |
| Topoisomerase Inhibitors | ETIRINOTECAN PEGOL, ETOPOSIDE, IRINOTECAN, TOPOTECAN, |
| Vinca Alkaloids | VINCRISTINE, VINORELBINE |

## Supplementary Figures


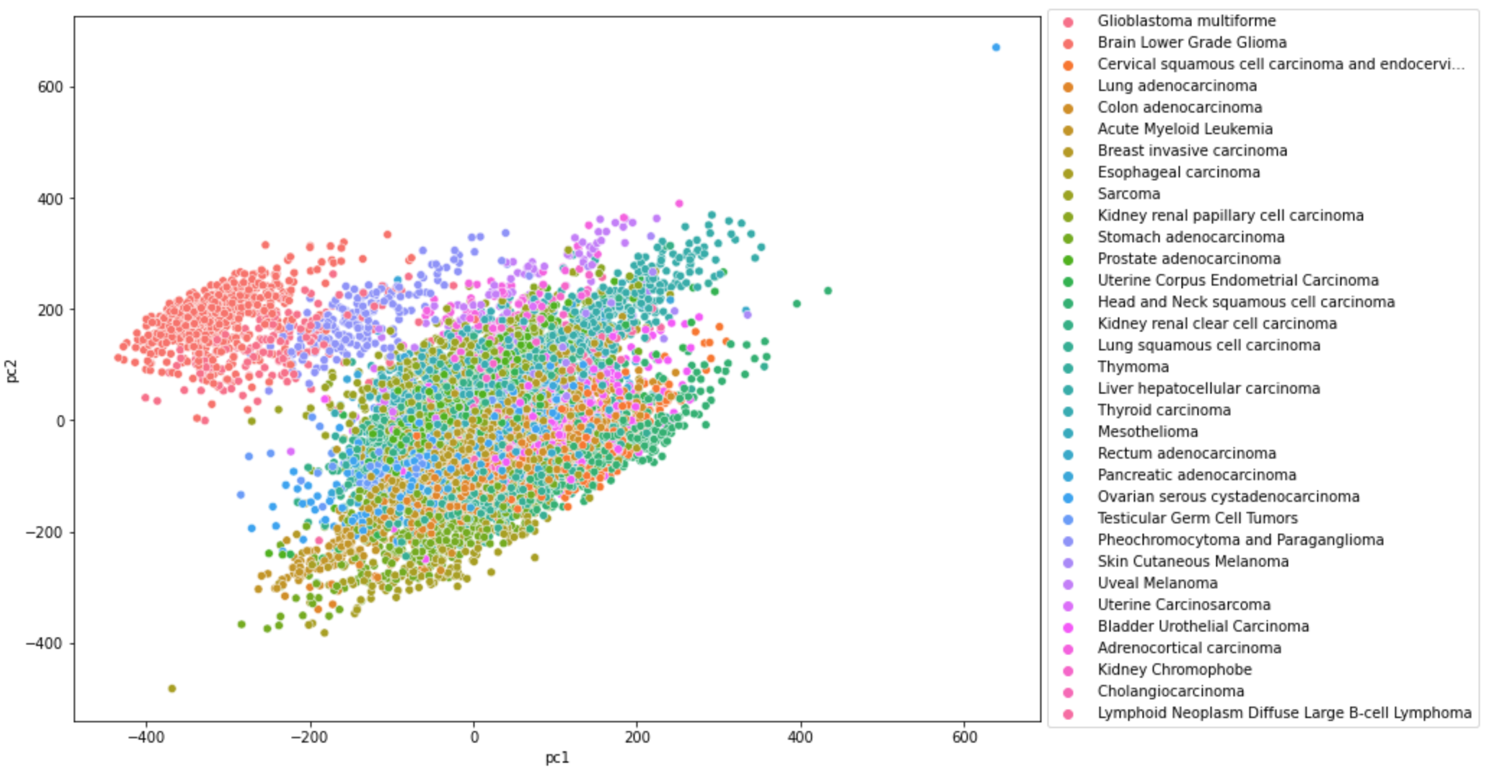


**Supplementary Figure 1A.** The PCA plot of all primary TCGA tumors. Colors represent different cancer types.


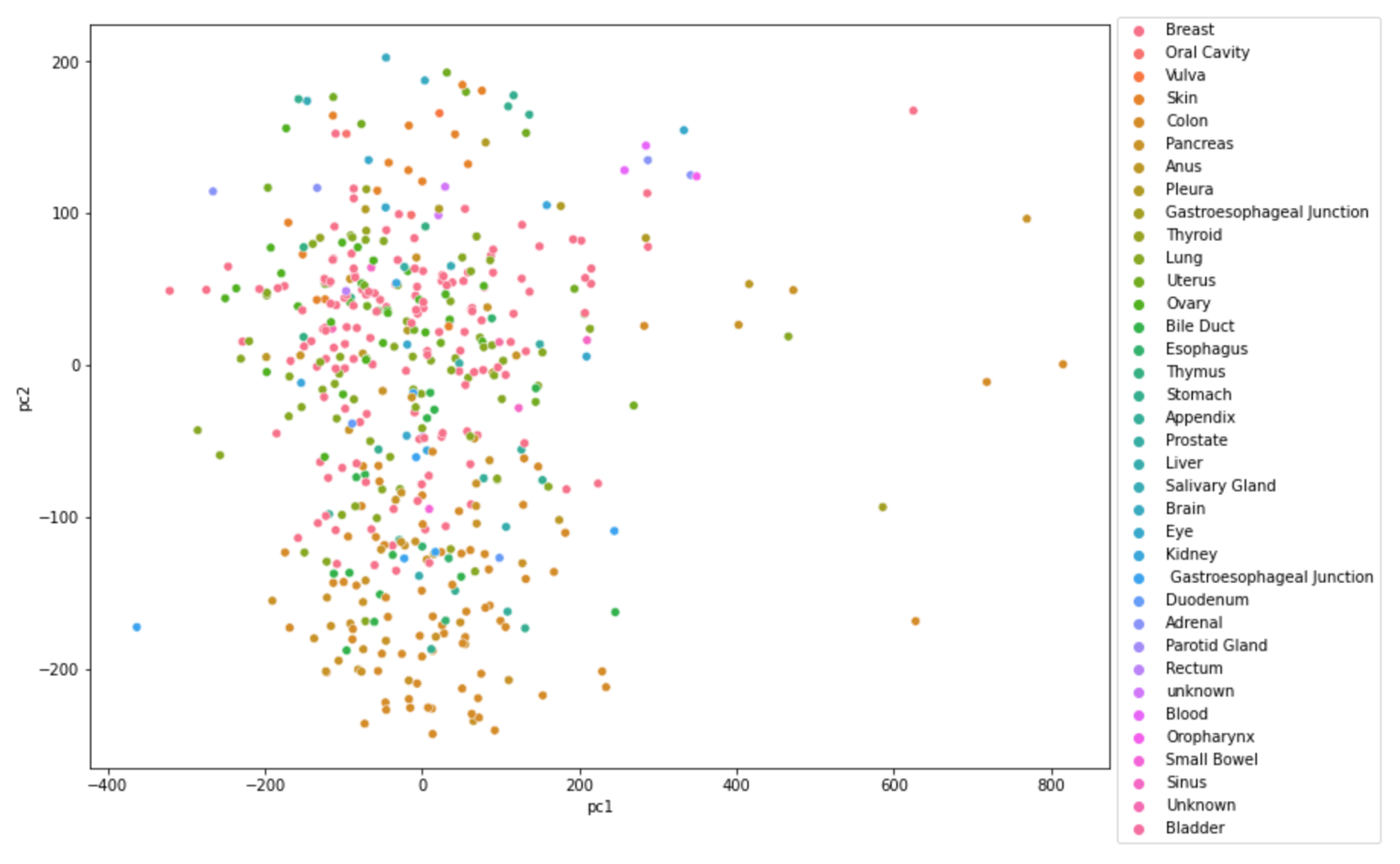


**Supplementary Figure 1B.** The PCA plot of all metastatic tumors. Colors represent tumor primary sites.


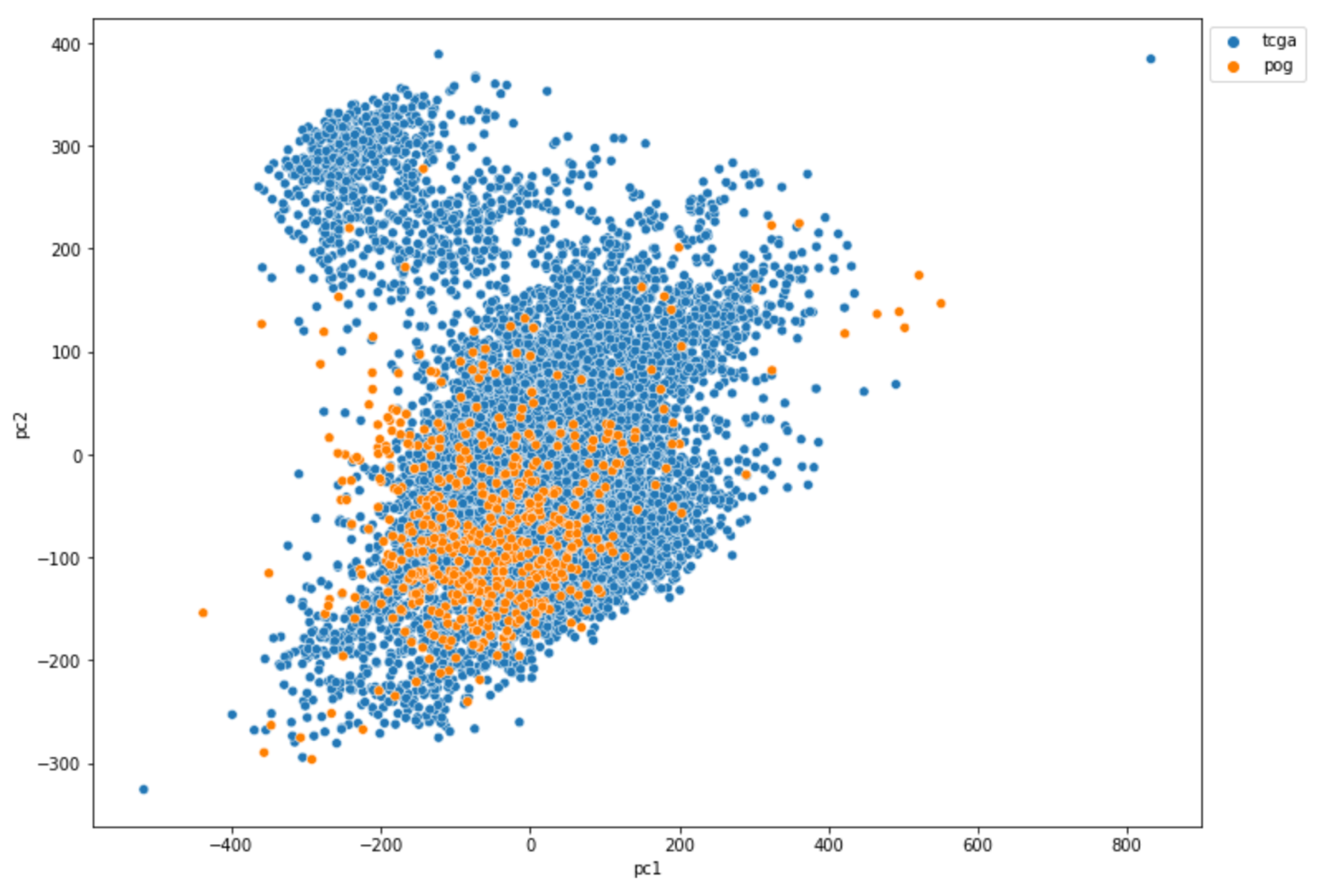


**Supplementary Figure 1C.** The PCA plot of all primary (TCGA) and metastatic (POG) tumors. Colors represent the studies.


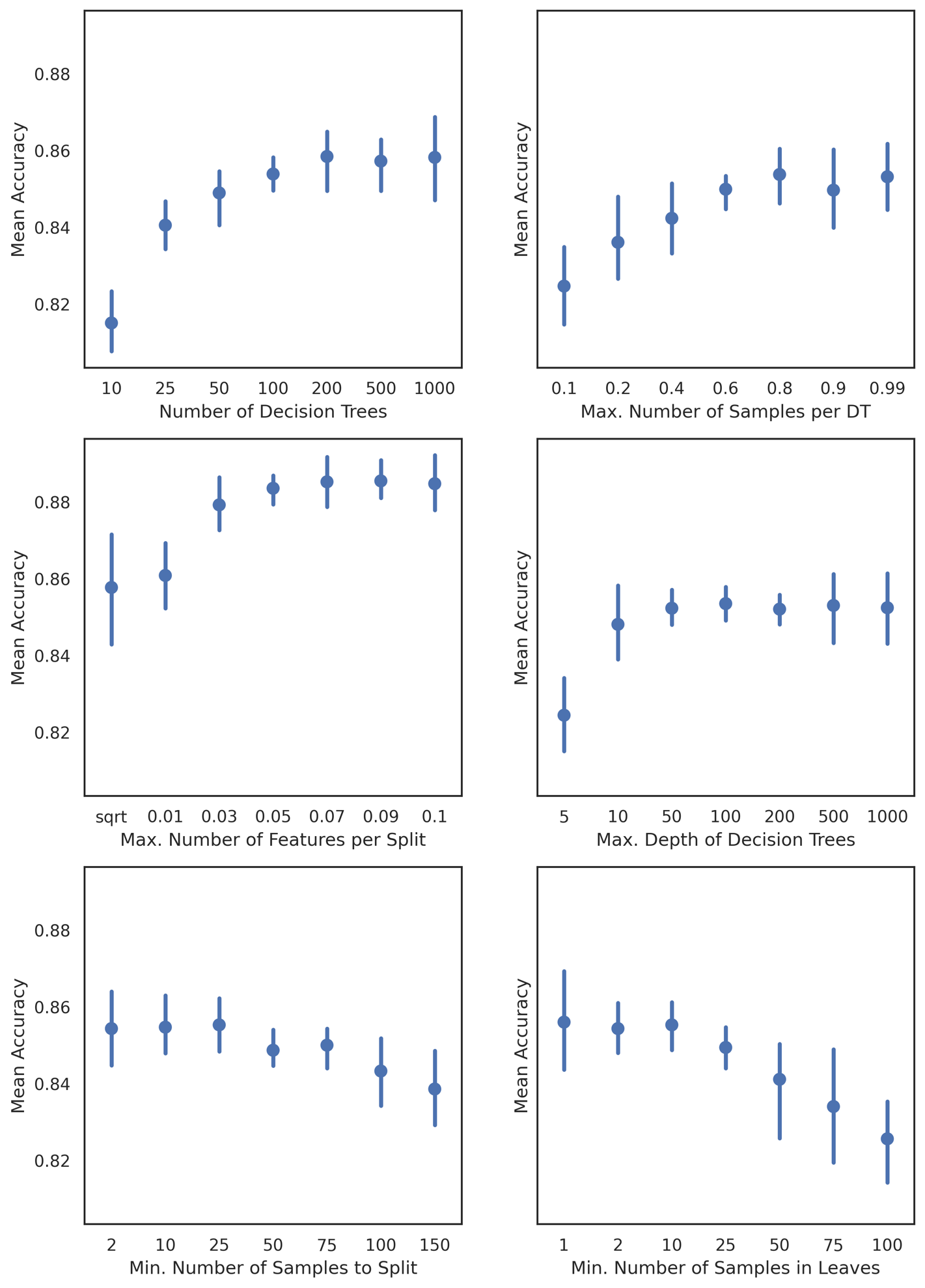


**Supplementary Figure 2.** Mean prediction accuracy across 5-folds of 90% of the merged set samples versus various values of the random forest hyperparameters. Except for the hyperparameter being evaluated, the rest of the hyperparameters are fixed using the random forest classifier default values.


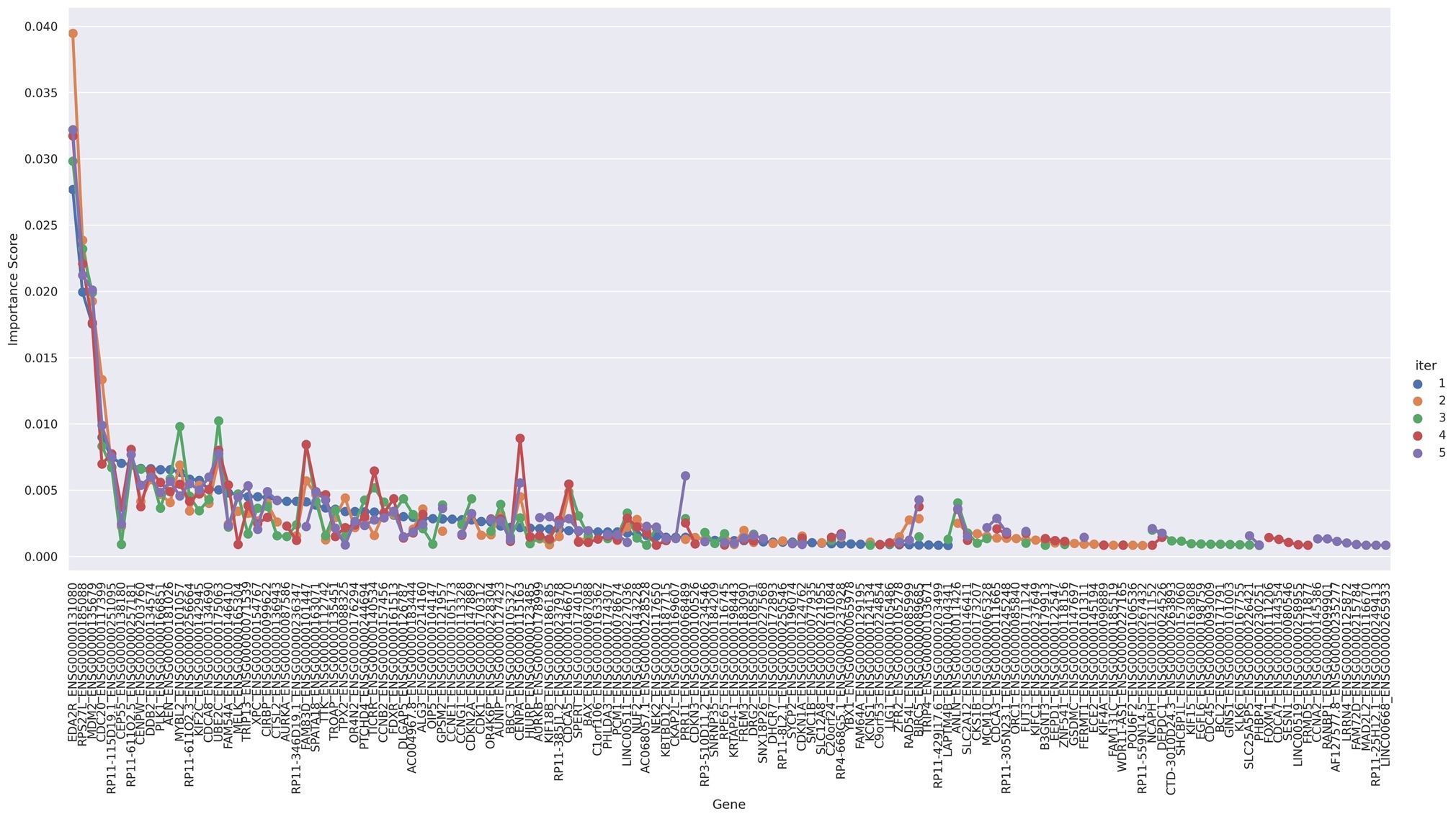


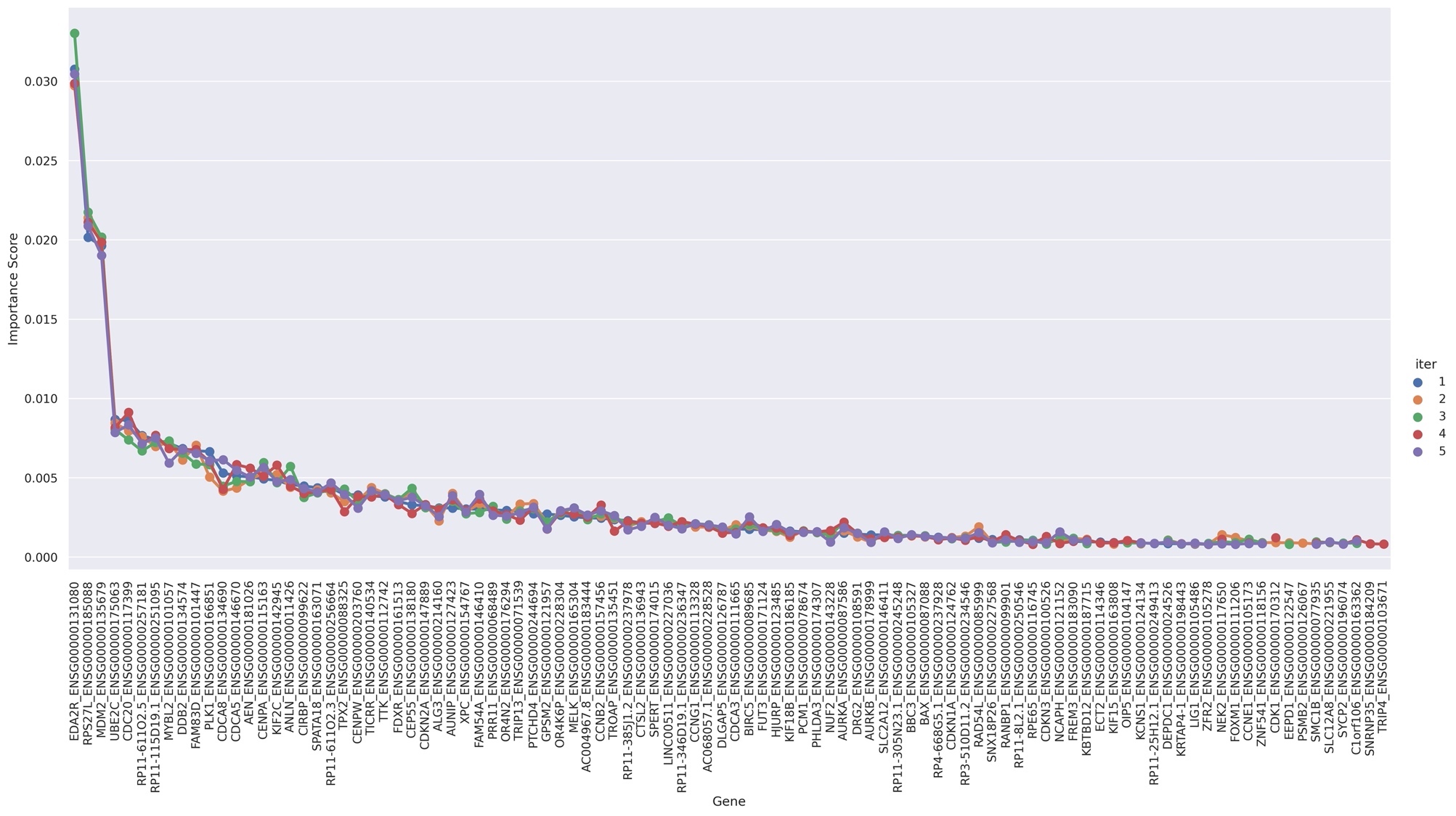


**Supplementary Figure 3.** Top features with importance scores > 0.00085 (top) and > 0.0008 (bottom) extracted from the random forest model after classifying samples into impactful p53 mutant and wildtype p53 categories. Each color is an independent iteration of training the model using 200 (top) and 3000 trees (bottom).


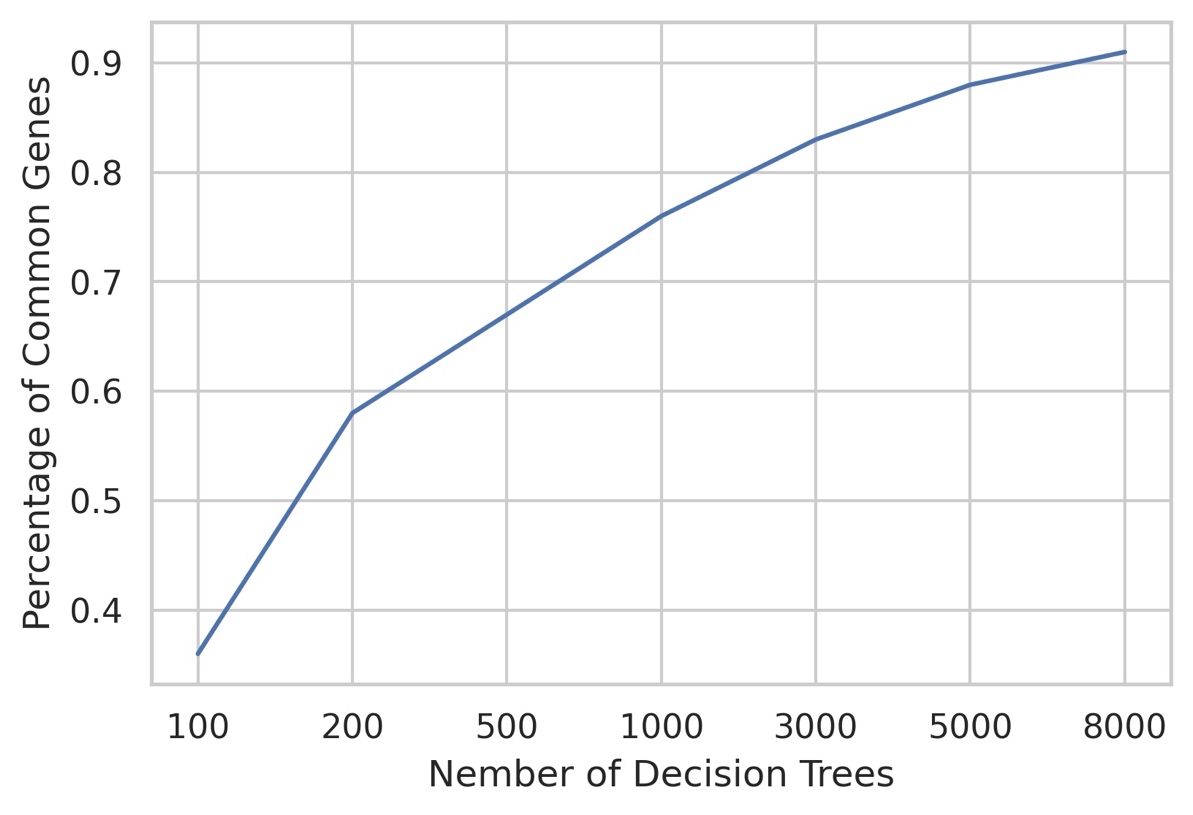


**Supplementary Figure 4.** Percentage of the common genes found in the list of top 100 genes with the highest importance scores across 10 independent iterations of training the random forest using the merged set.


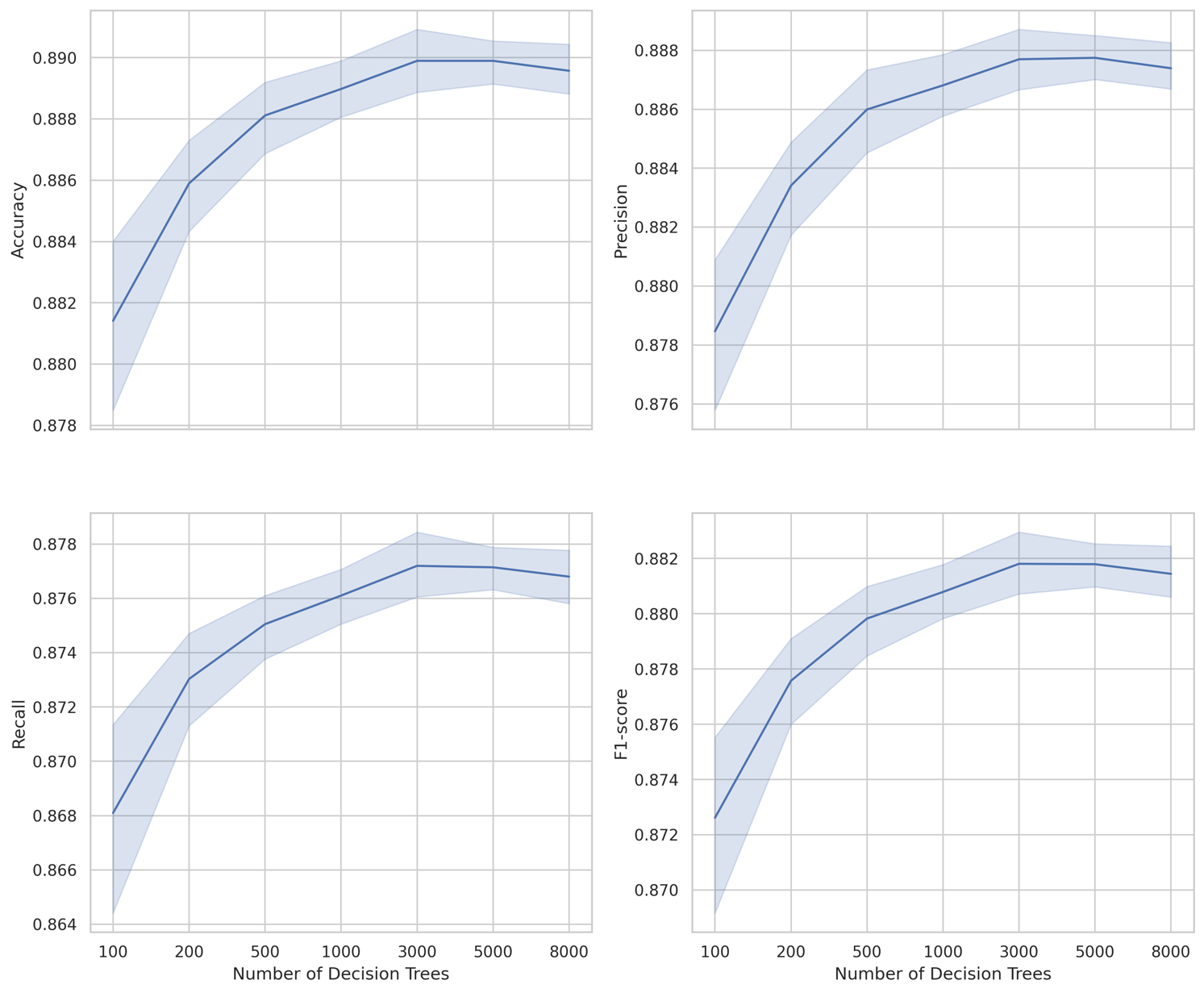


**Supplementary Figure 5.** Performance of the random forest on the 20% held out set after training the algorithm using 80% of the merged set samples and different number of decision trees.


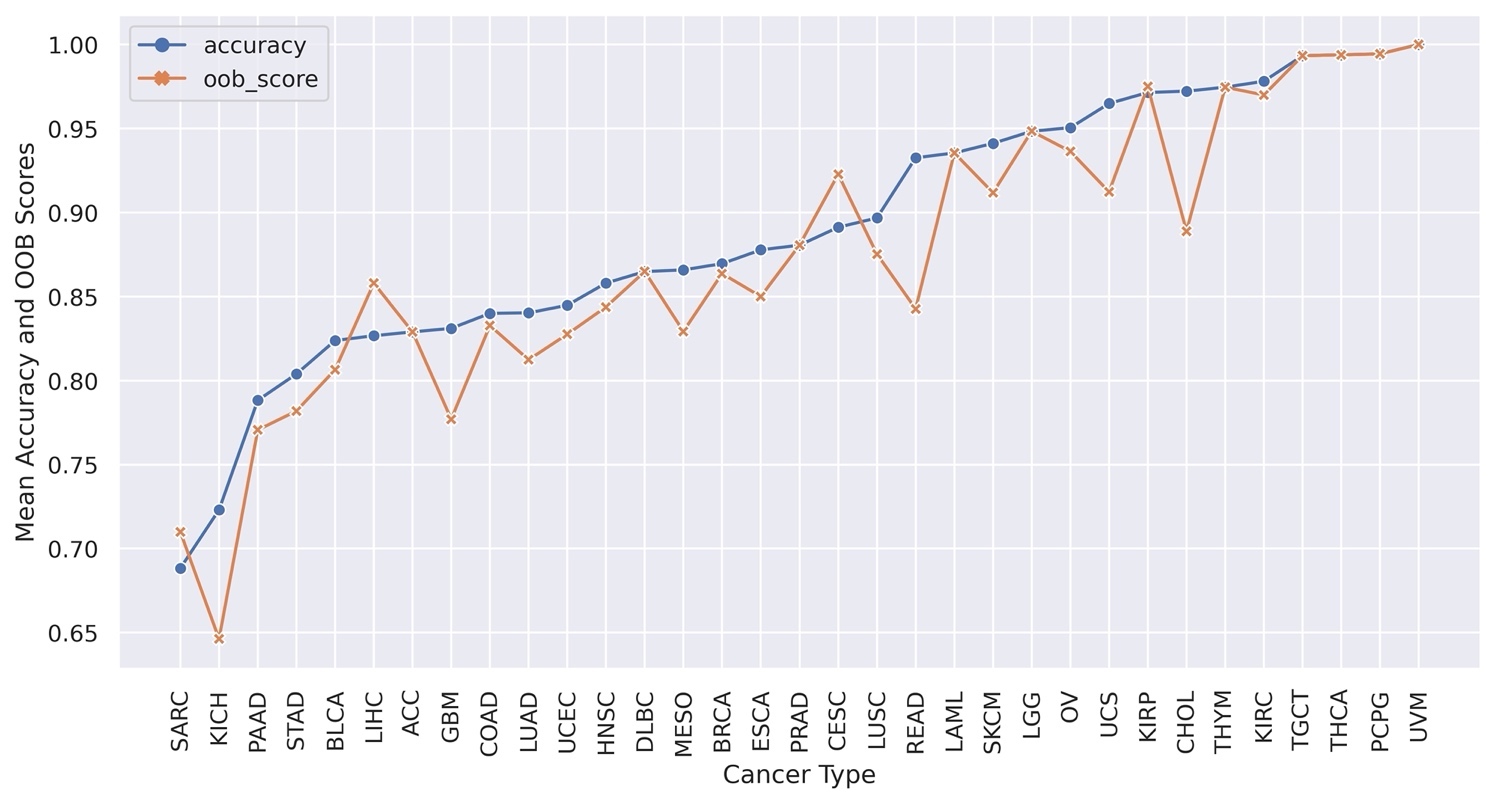


**Supplementary Figure 6.** Graph of mean accuracies and OOB scores versus cancer types (ACC=Adrenocortical carcinoma, BLCA=Bladder Urothelial Carcinoma, BRCA=Breast invasive carcinoma, CESC=Cervical squamous cell carcinoma and endocervical adenocarcinoma, CHOL=Cholangiocarcinoma, COAD=Colon adenocarcinoma, DLBC=Lymphoid Neoplasm Diffuse Large B-cell Lymphoma, ESCA=Esophageal carcinoma, GBM=Glioblastoma multiforme, HNSC=Head and Neck squamous cell carcinoma, KICH=Kidney Chromophobe, KIRC=Kidney renal clear cell carcinoma, KIRP=Kidney renal papillary cell carcinoma, LAML=Acute Myeloid Leukemia, LGG=Brain Lower Grade Glioma, LIHC=Liver hepatocellular carcinoma, LUAD=Lung adenocarcinoma, LUSC=Lung squamous cell carcinoma, MESO=Mesothelioma, OV=Ovarian serous cystadenocarcinoma, PAAD=Pancreatic adenocarcinoma, PCPG=Pheochromocytoma and Paraganglioma, PRAD=Prostate adenocarcinoma, READ=Rectum adenocarcinoma, SARC=Sarcoma, SKCM=Skin Cutaneous Melanoma, STAD=Stomach adenocarcinoma, TGCT=Testicular Germ Cell Tumors, THCA=Thyroid carcinoma, THYM=Thymoma, UCEC=Uterine Corpus Endometrial Carcinoma, UCS=Uterine Carcinosarcoma, UVM=Uveal Melanoma).


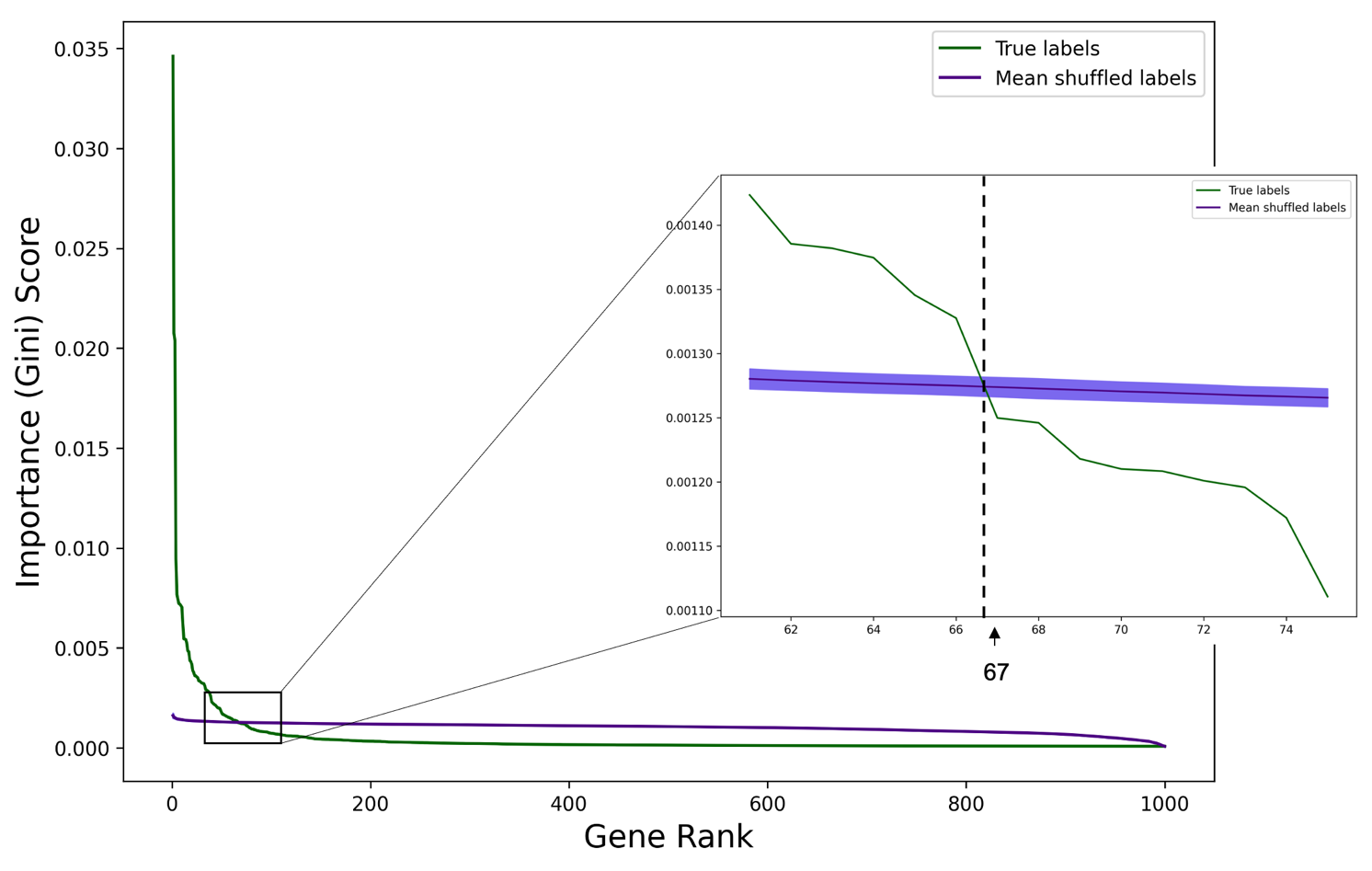


**Supplementary Figure 7.** The importance scores versus gene ranks extracted from the model trained using true labels (green) and the mean and standard deviation of importance scores of the gene ranks over 100 permutations of training the random forest with randomly shuffled labels (purple).


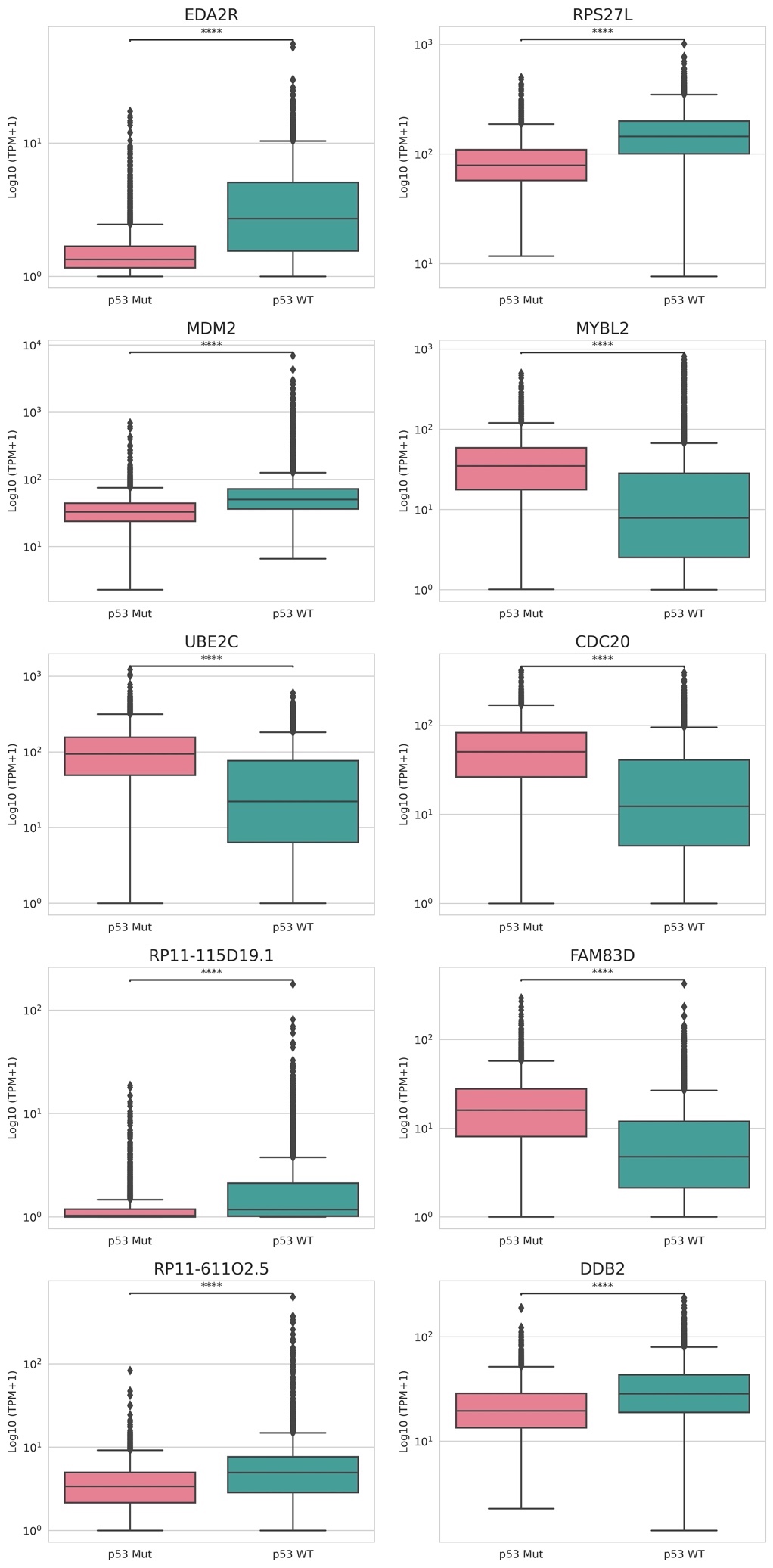


**Supplementary Figure 8.** Boxplots of expression values of the top 10 genes with the highest importance scores in classification in samples with mutant *TP53* versus wildtype (the p-values are found in a Mann-Whitney-Wilcoxon two-sided test with Bonferroni correction; p-value annotation guide: ns: 5.00e-02 < p <= 1.00, *: 1.00e-02 < p <= 5.00e-2, **: 1.00e-03 < p <= 1.00e-02, ***: 1.00e-04 < p <= 1.00e-03, ****: p <= 1.00e-04).


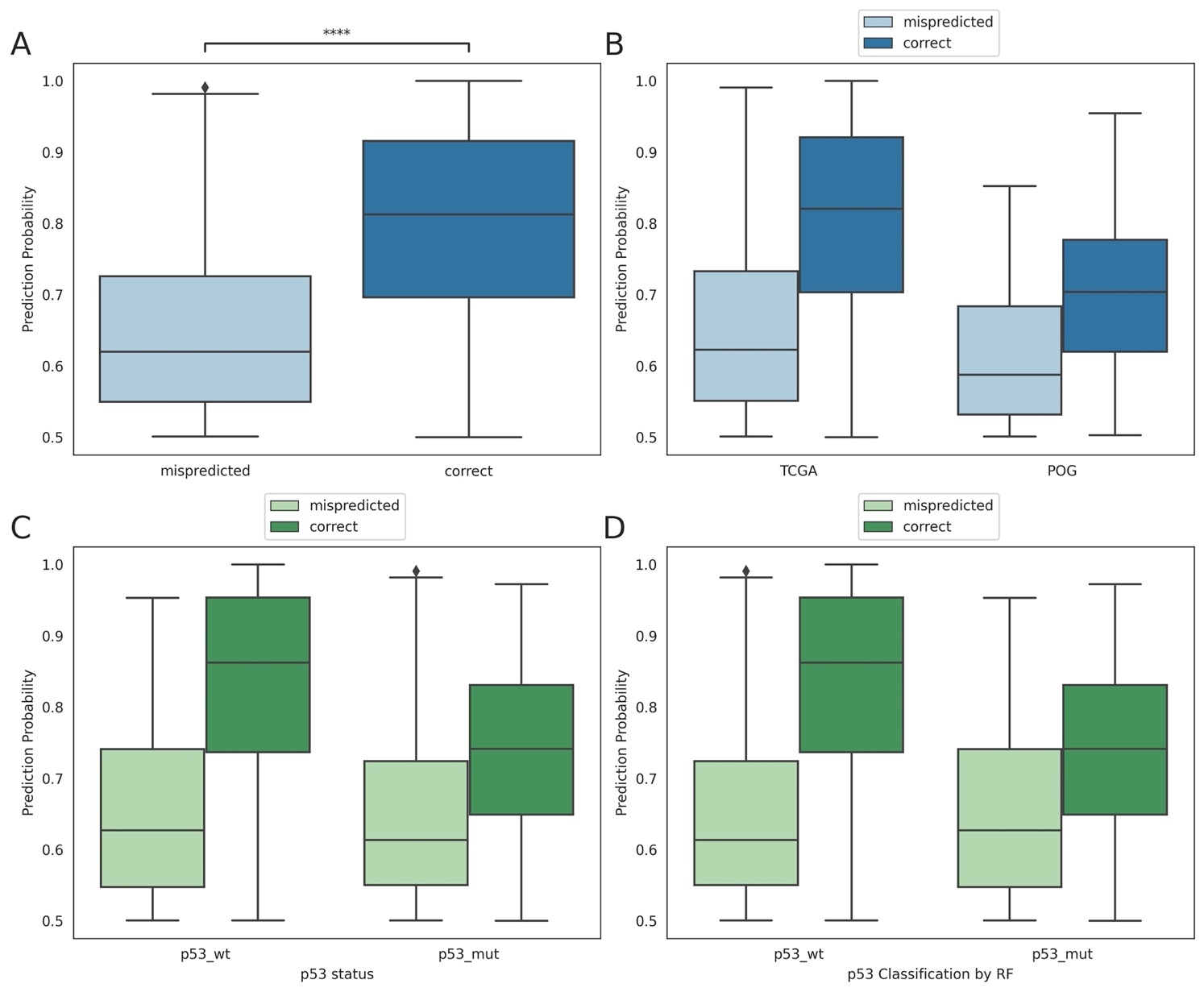


**Supplementary Figure 9.** Prediction probabilities grouped by A) prediction correctness and, B) samples sources (TCGA vs POG), C) p53 status, and D) predicted p53 status (the p-value in A is found in a Mann-Whitney-Wilcoxon two-sided test with Bonferroni correction; p-value annotation guide: ns: 5.00e-02 < p <= 1.00, *: 1.00e-02 < p <= 5.00e-2, **: 1.00e-03 < p <= 1.00e-02, ***: 1.00e-04 < p <= 1.00e-03, ****: p <= 1.00e-04).


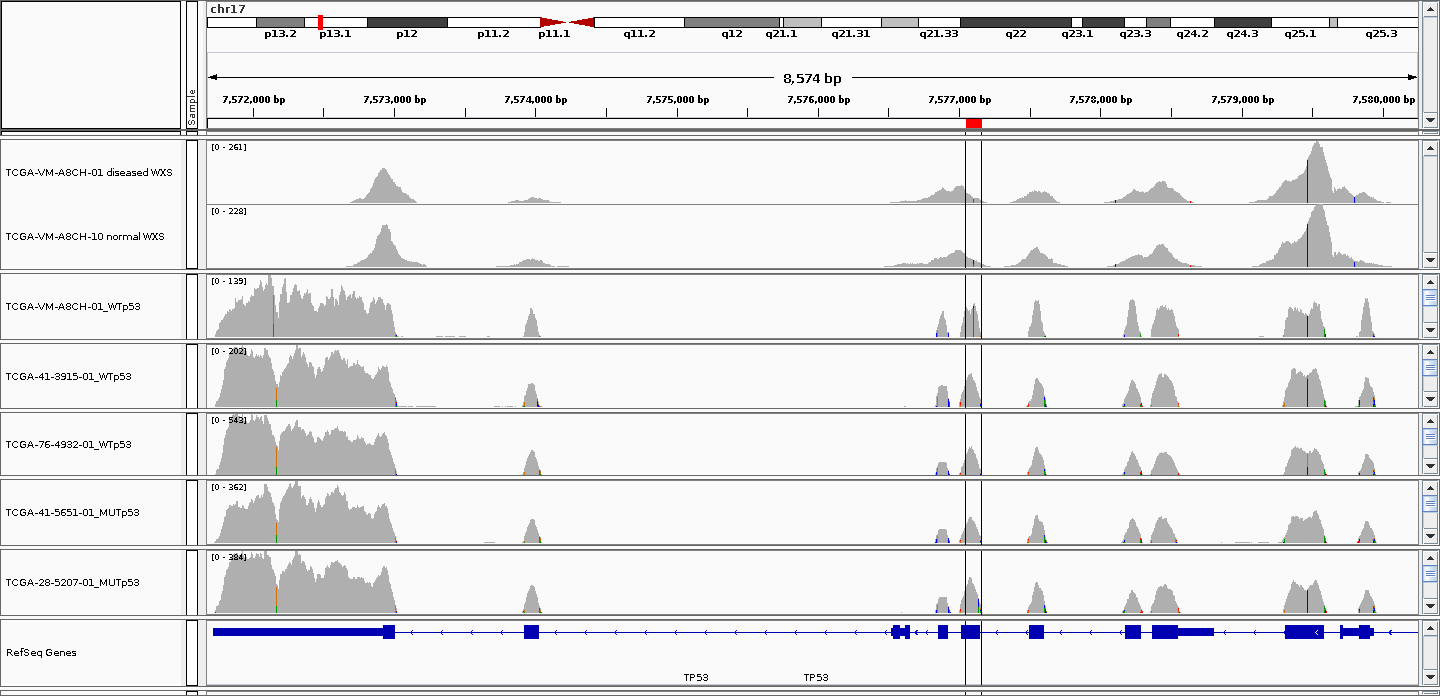


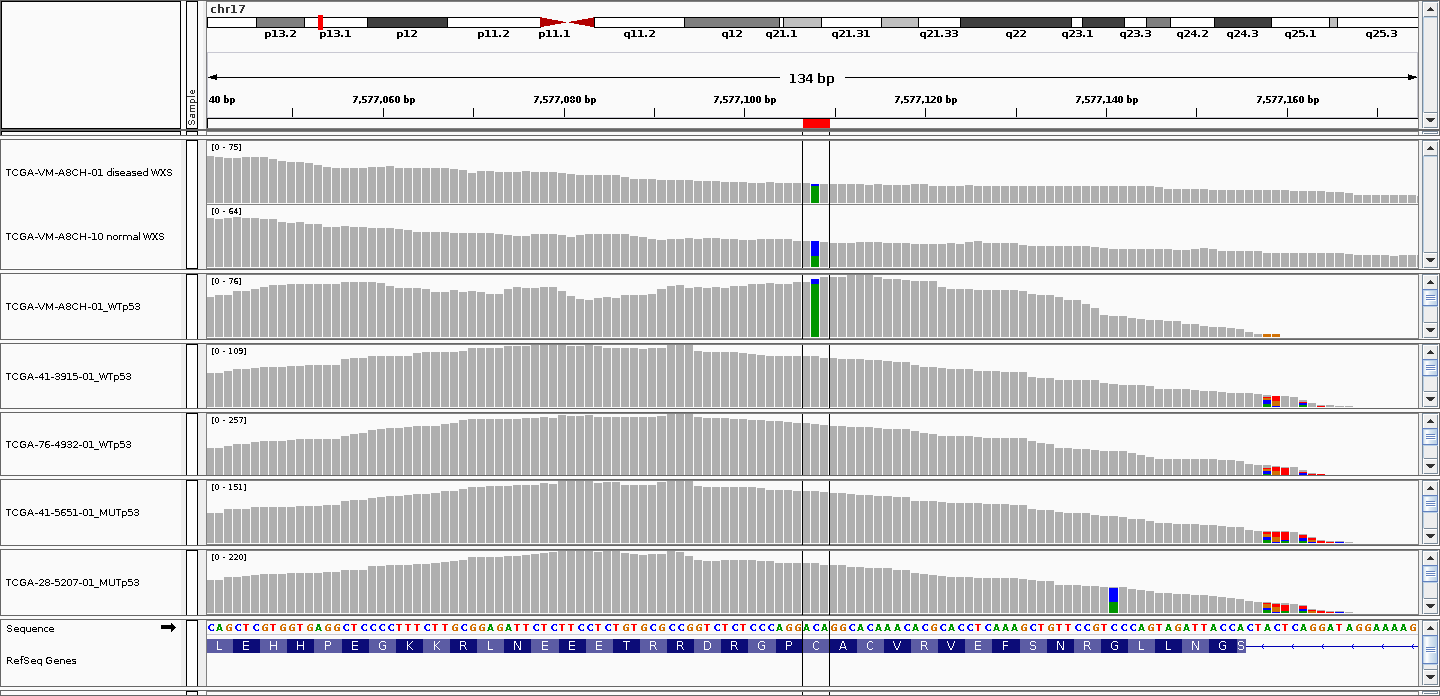


**Supplementary Figure 10A.** RNA-seq and WXS tracks of the brain lower grade glioma mispredicted sample with high prediction probability (TCGA-VM-A8CH-01) in addition to RNA-seq tracks of other brain tumor samples (glioblastoma multiforme) with and without p53 mutations.


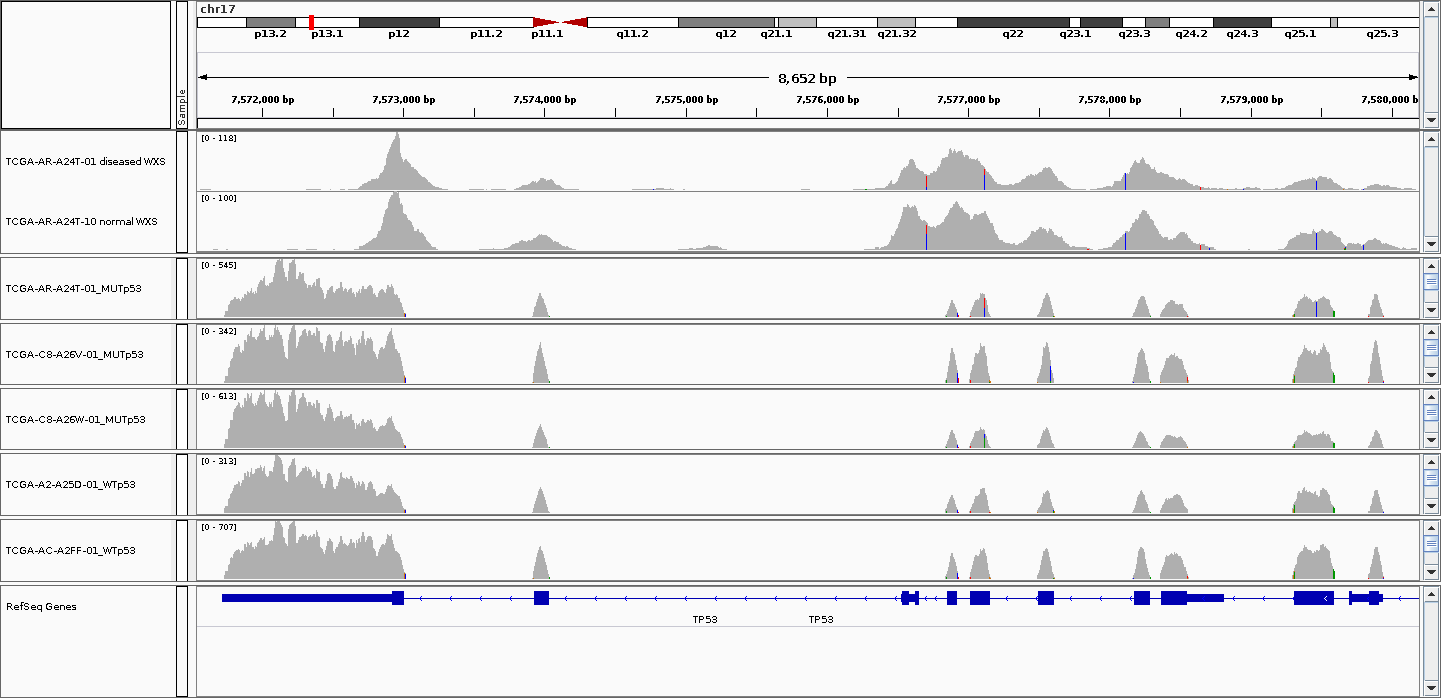


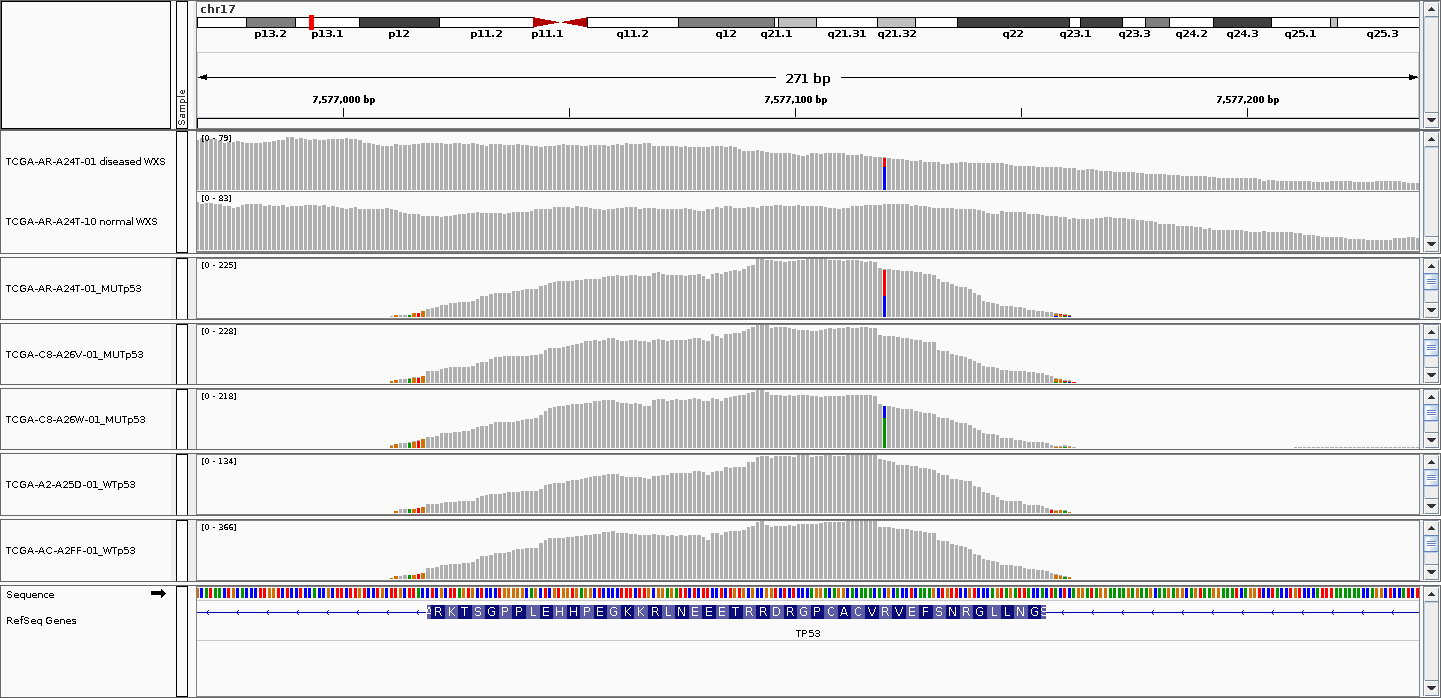


**Supplementary Figure 10B.** RNA-seq and WXS tracks of the breast invasive carcinoma mispredicted sample with high prediction probability (TCGA-AR-A24T-01) in addition to RNA-seq tracks of other breast invasive carcinoma samples with and without p53 mutations.


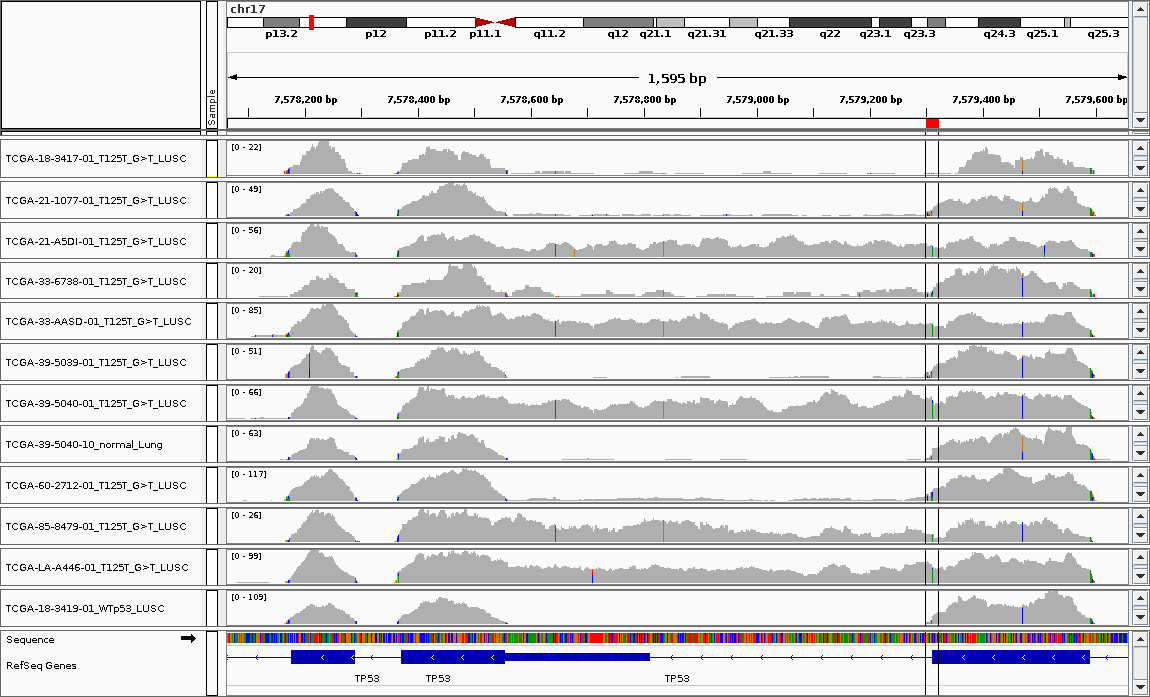


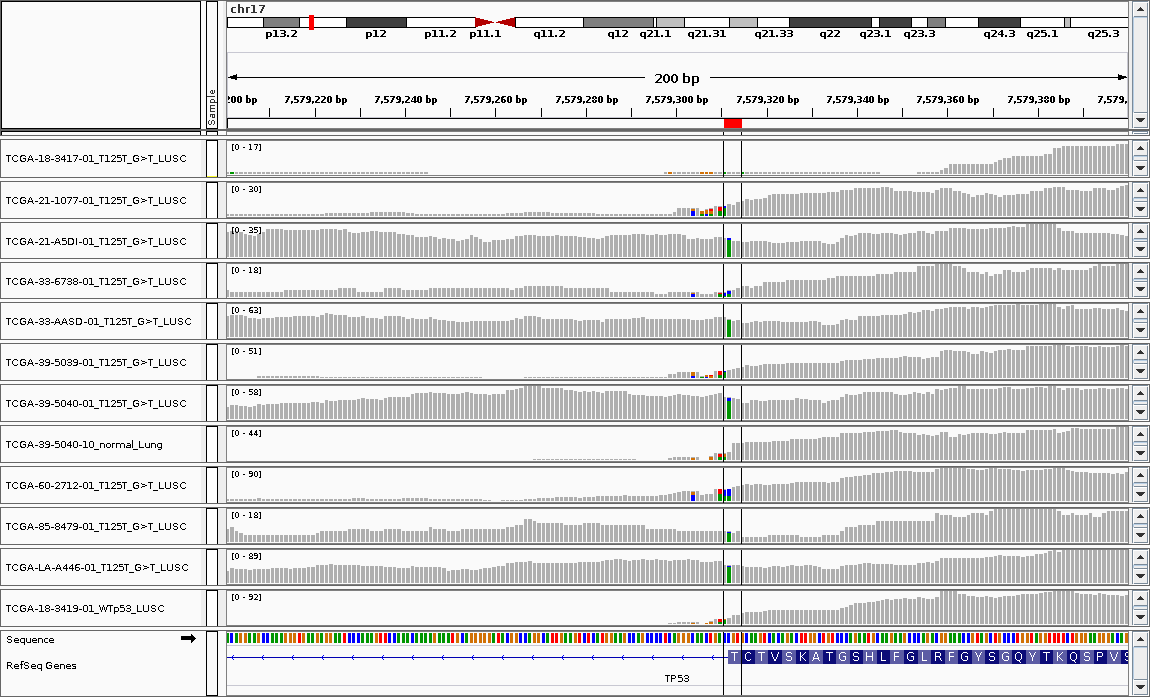


**Supplementary Figure 11A.** RNA-seq tracks of lung squamous cell carcinoma (LUSC) samples with silent mutations at Thr125 of p53 protein with specific nucleotide modification of G>T (the last track is from a LUSC sample with wildtype p53 copies, and the eighth track is from the normal lung tissue adjacent to the LUSC sample in the seventh track).


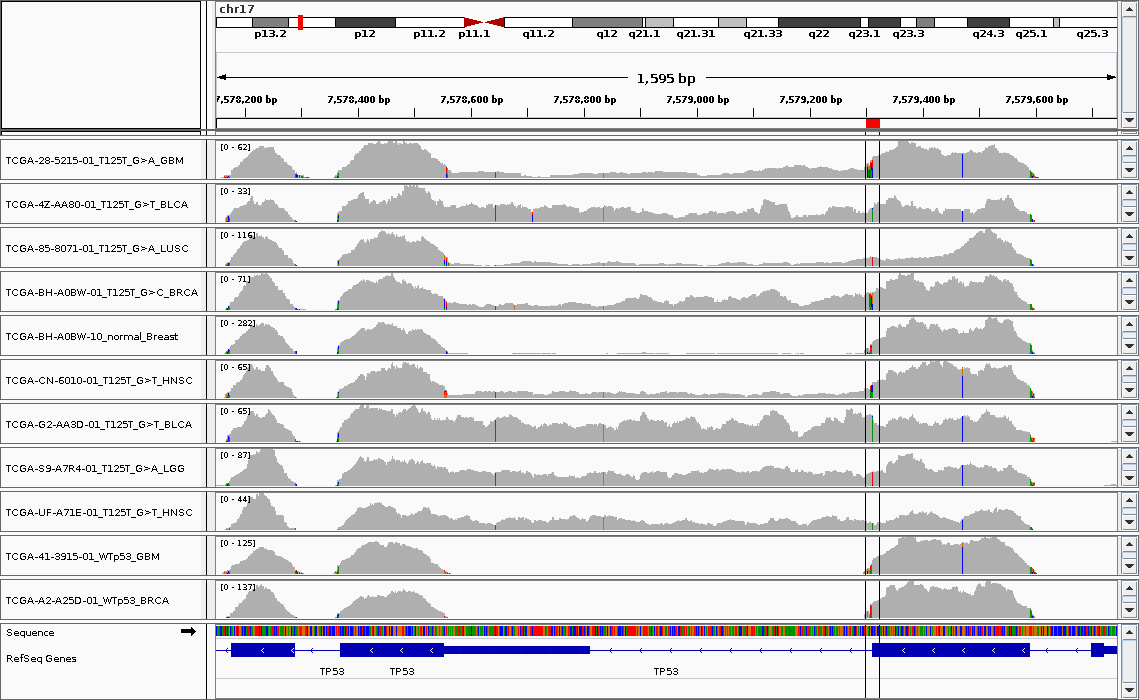


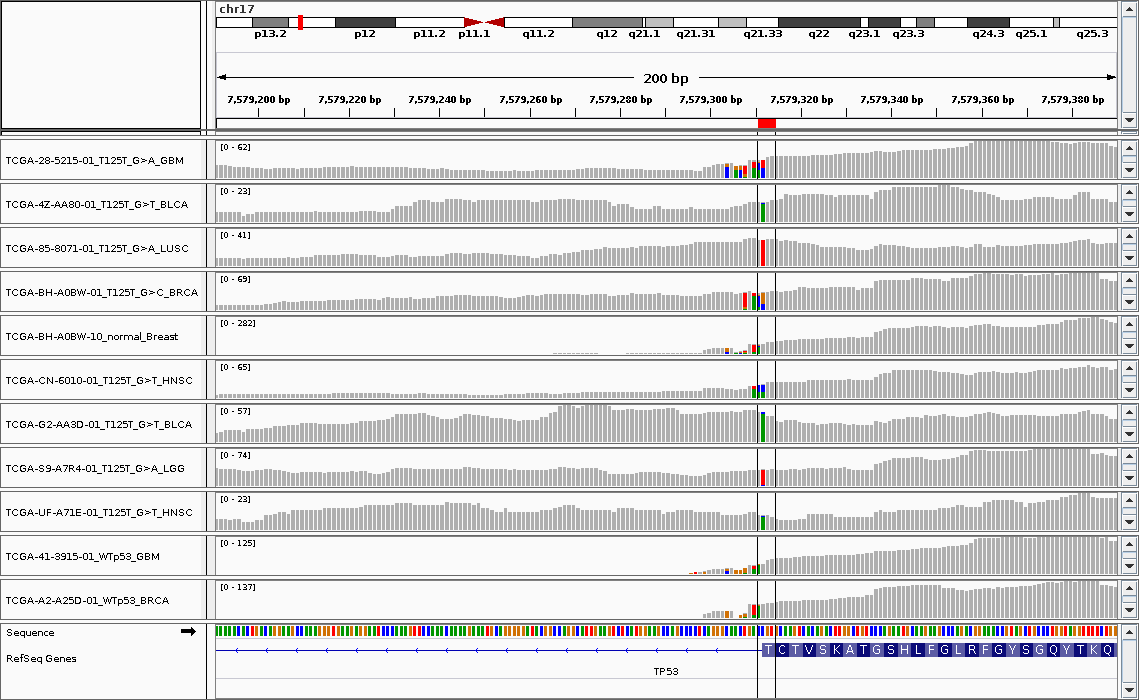


**Supplementary Figure 11B.** RNA-seq tracks of more tumor samples with silent mutations at Thr125 of p53 protein (the last two tracks are from a glioblastoma multiforme sample and a breast invasive carcinoma sample with wildtype p53 copies).


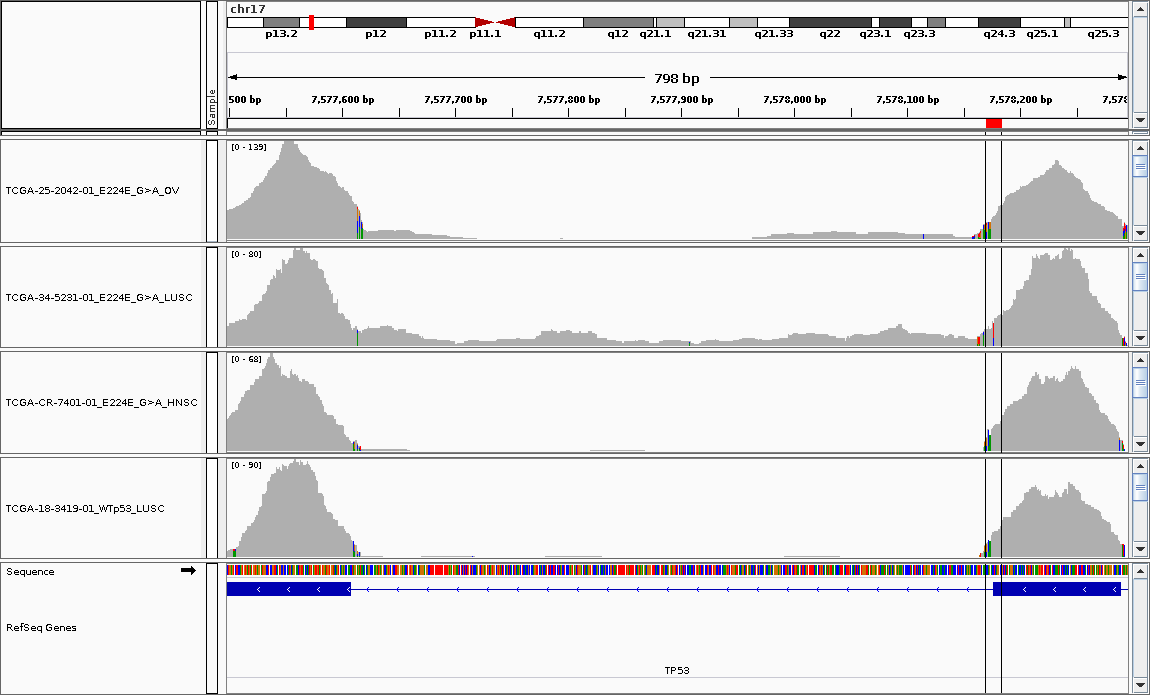


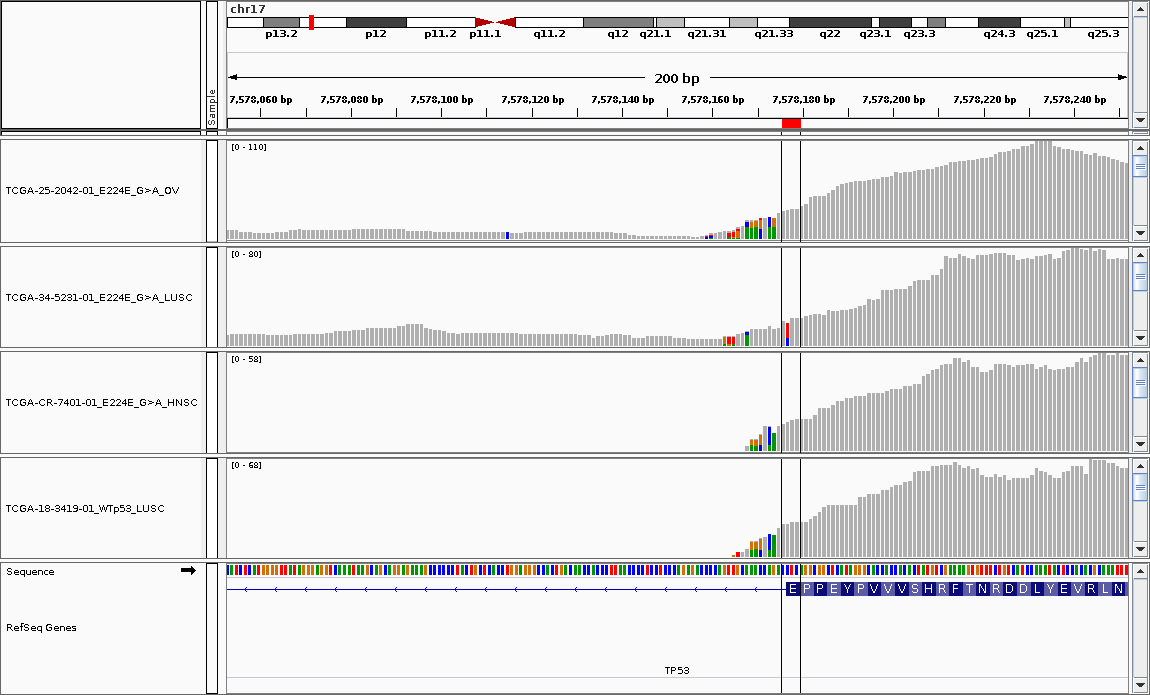


**Supplementary Figure 11C.** RNA-seq tracks of tumor samples with silent mutations at Glu224 of p53 protein with specific nucleotide modification of G>A (the last track is from a lung squamous cell carcinoma sample with wildtype p53 copies).


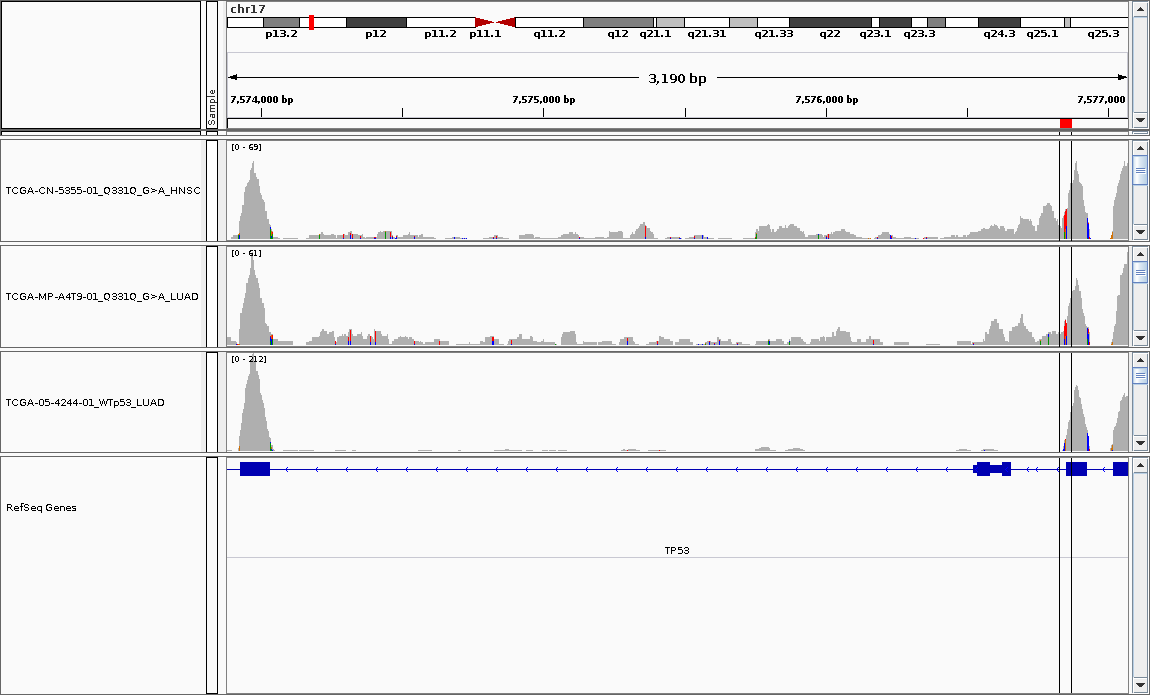


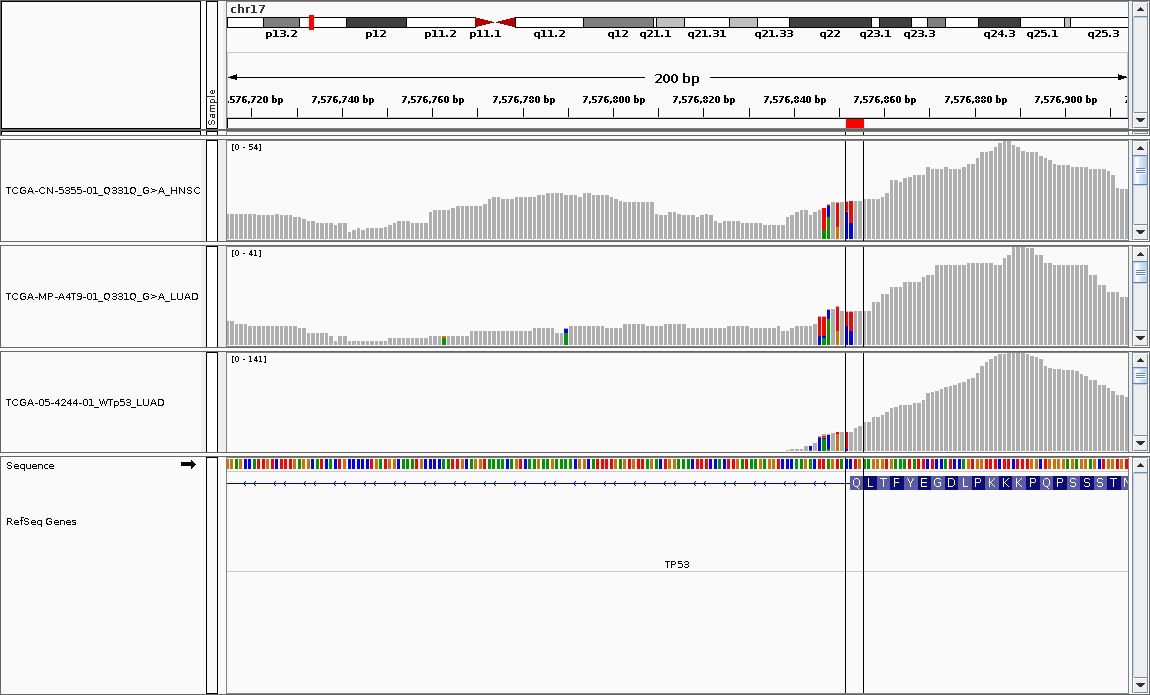


**Supplementary Figure 11D.** RNA-seq tracks of tumor samples with silent mutation at Gln331 of p53 protein with specific nucleotide modification of G>A (the last track is from a lung adenocarcinoma sample with wildtype p53 copies).


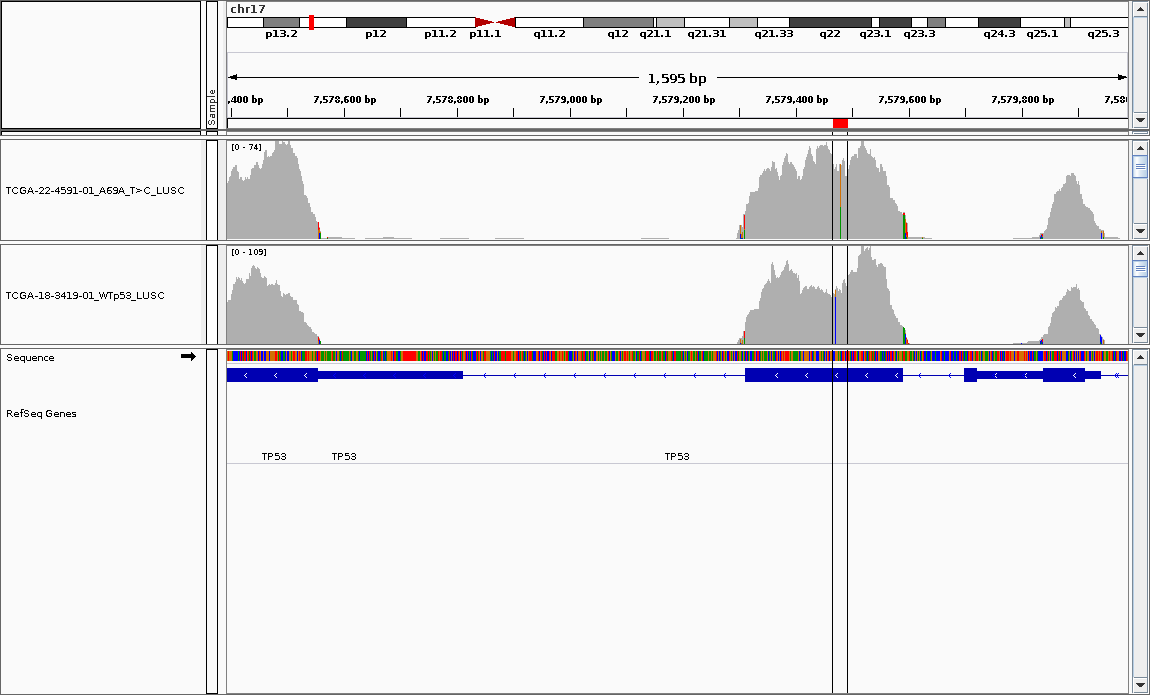


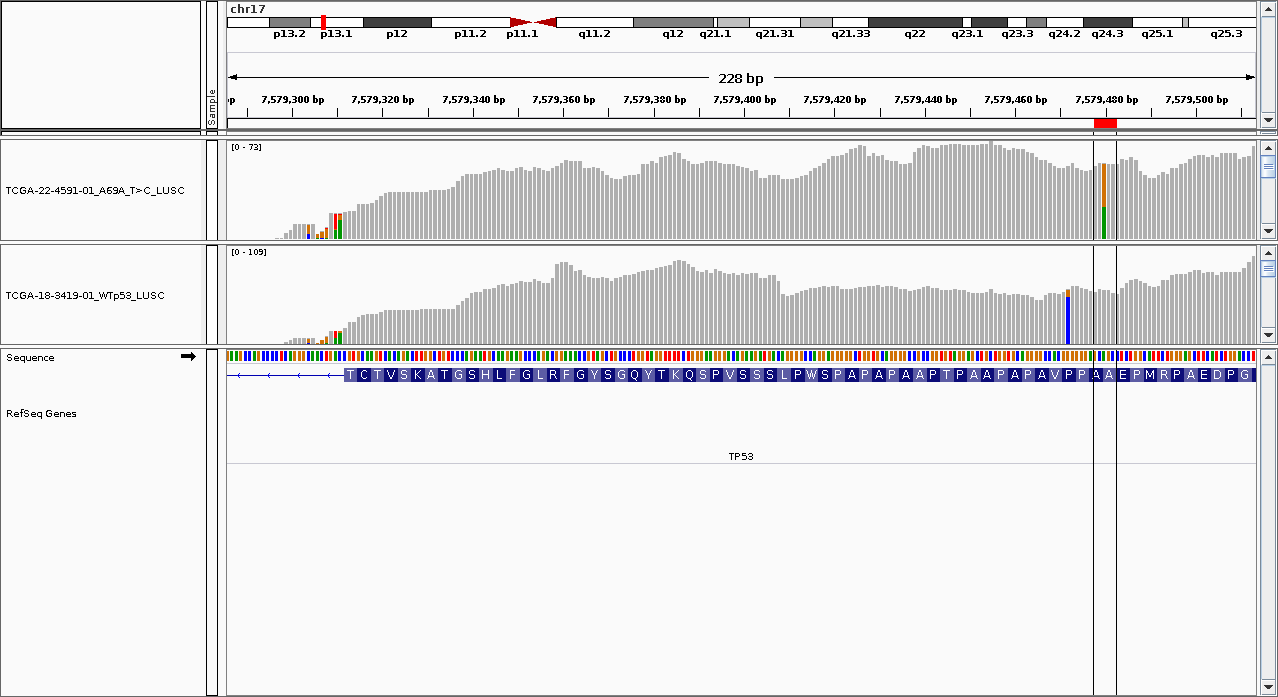


**Supplementary Figure 11E.** RNA-seq track of the lung squamous cell carcinoma (LUSC) sample with a silent mutation at Ala69 of p53 protein with specific nucleotide modification of C>T (top track) and RNA-seq track of a LUSC sample with wildtype p53 copies (bottom track).


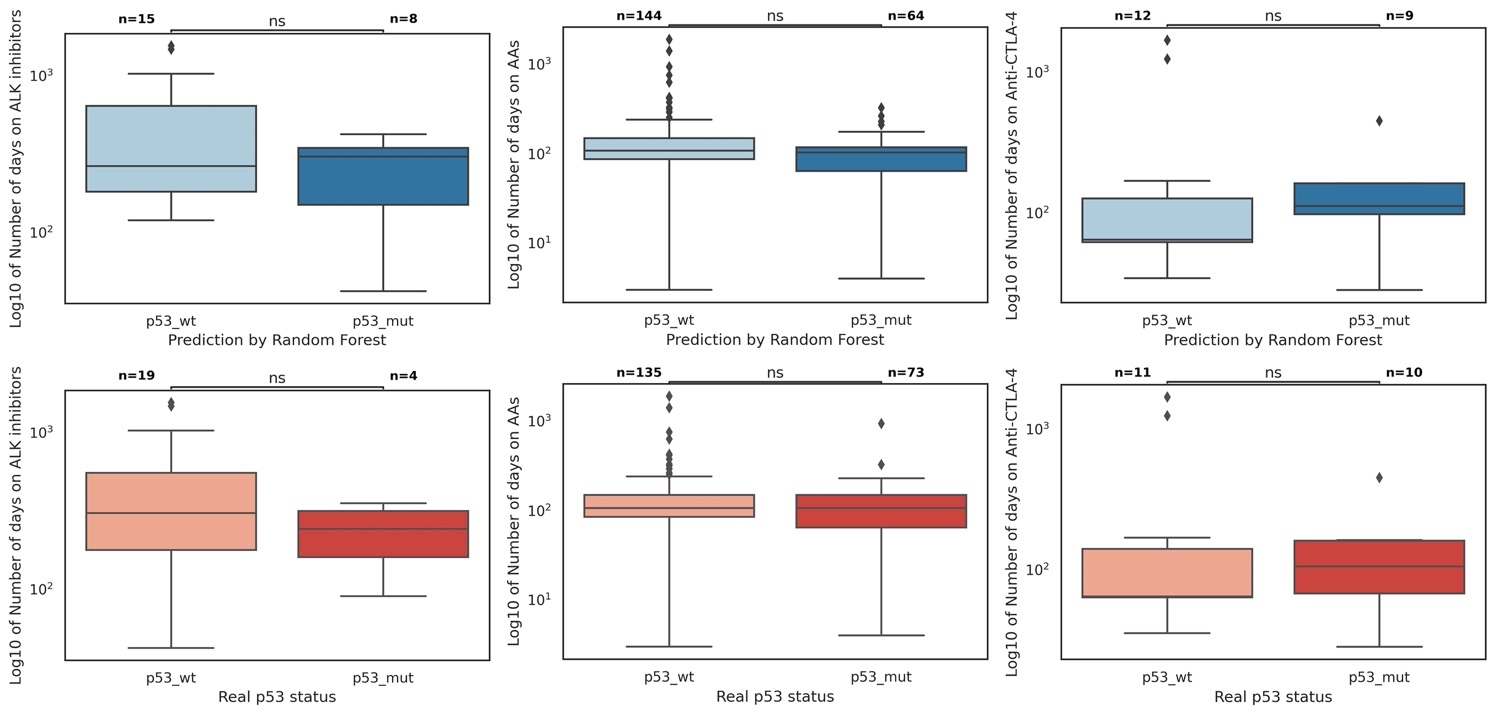


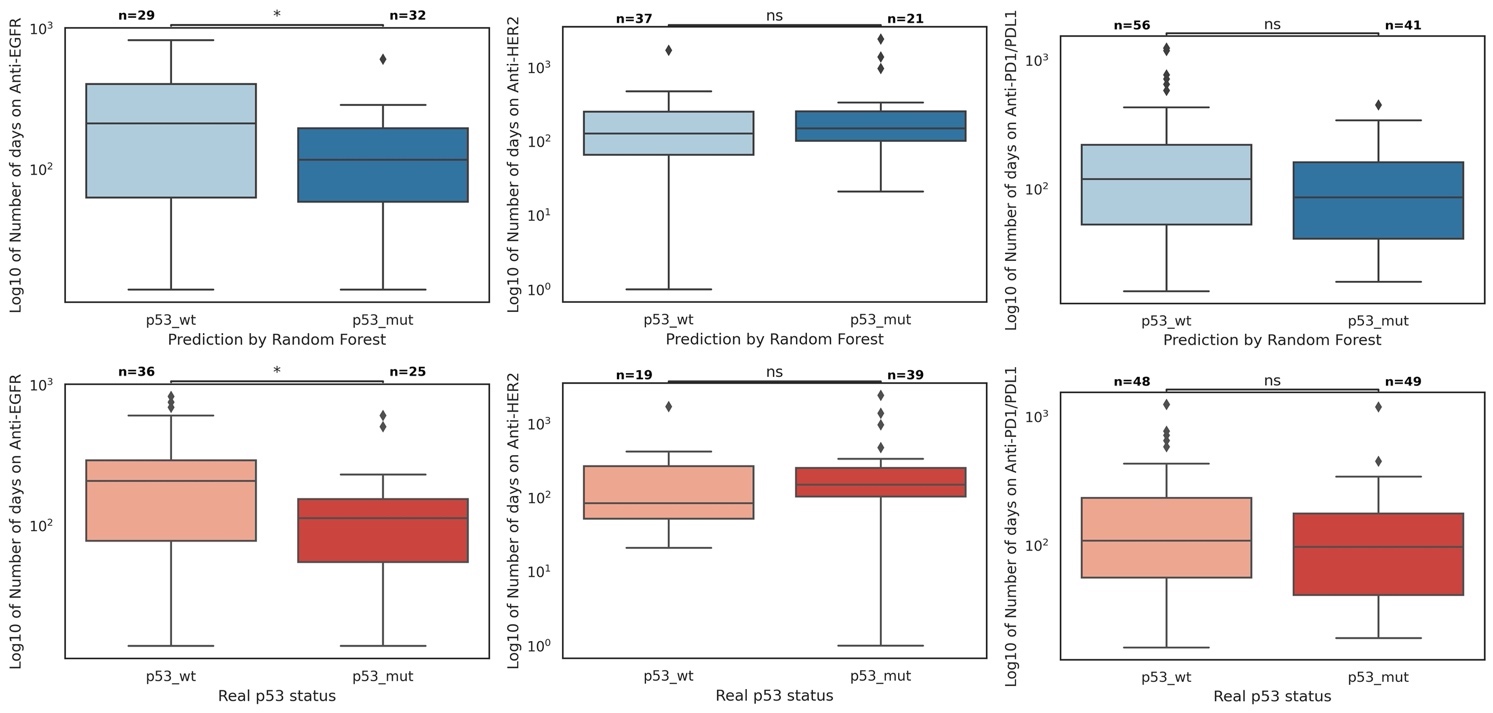


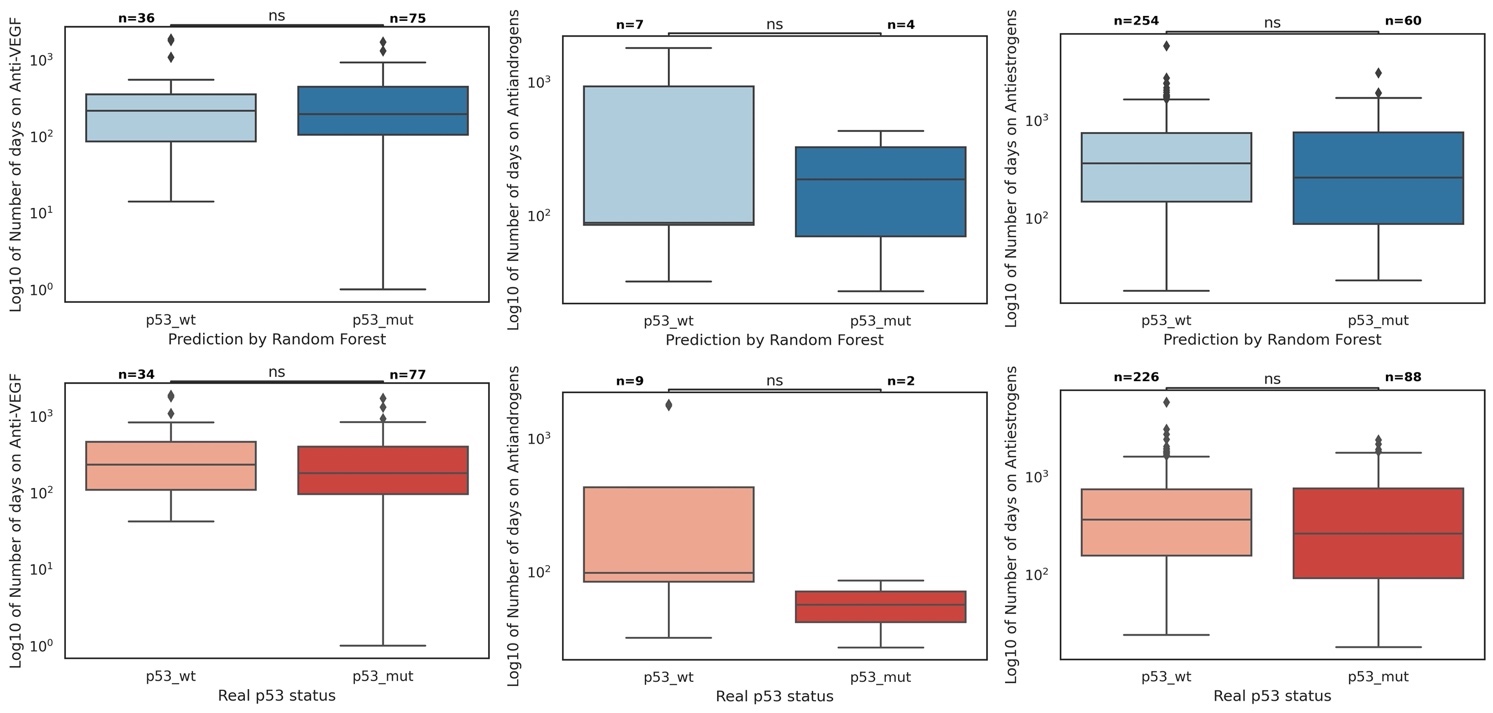


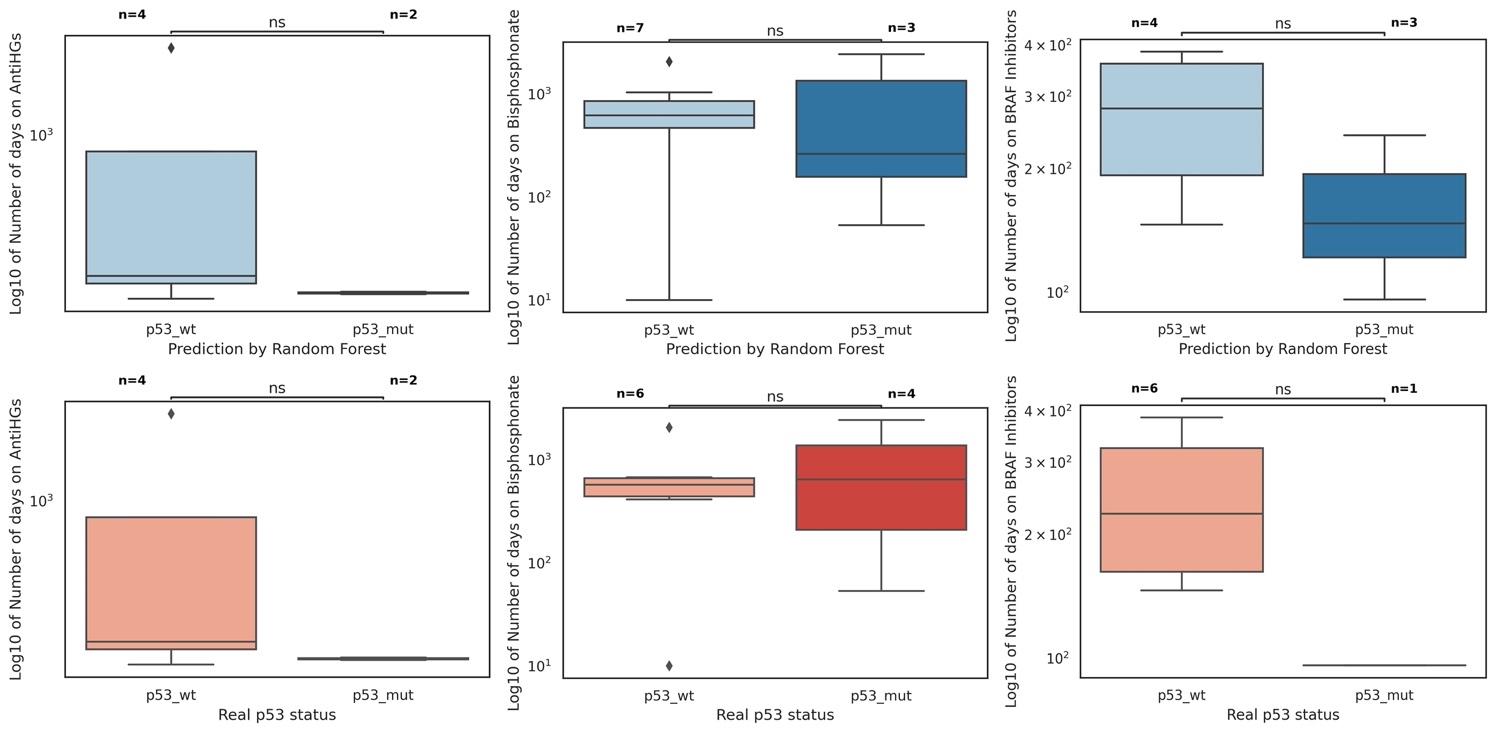


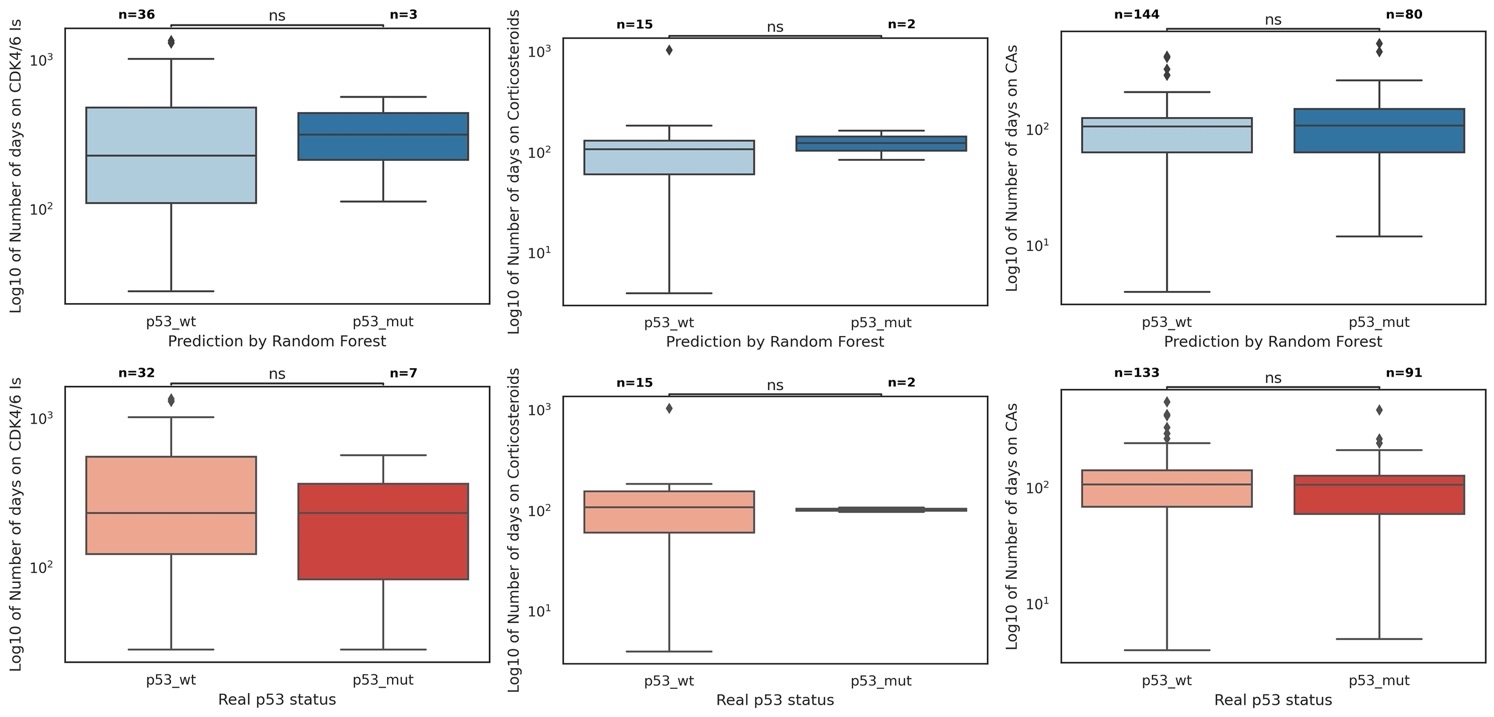


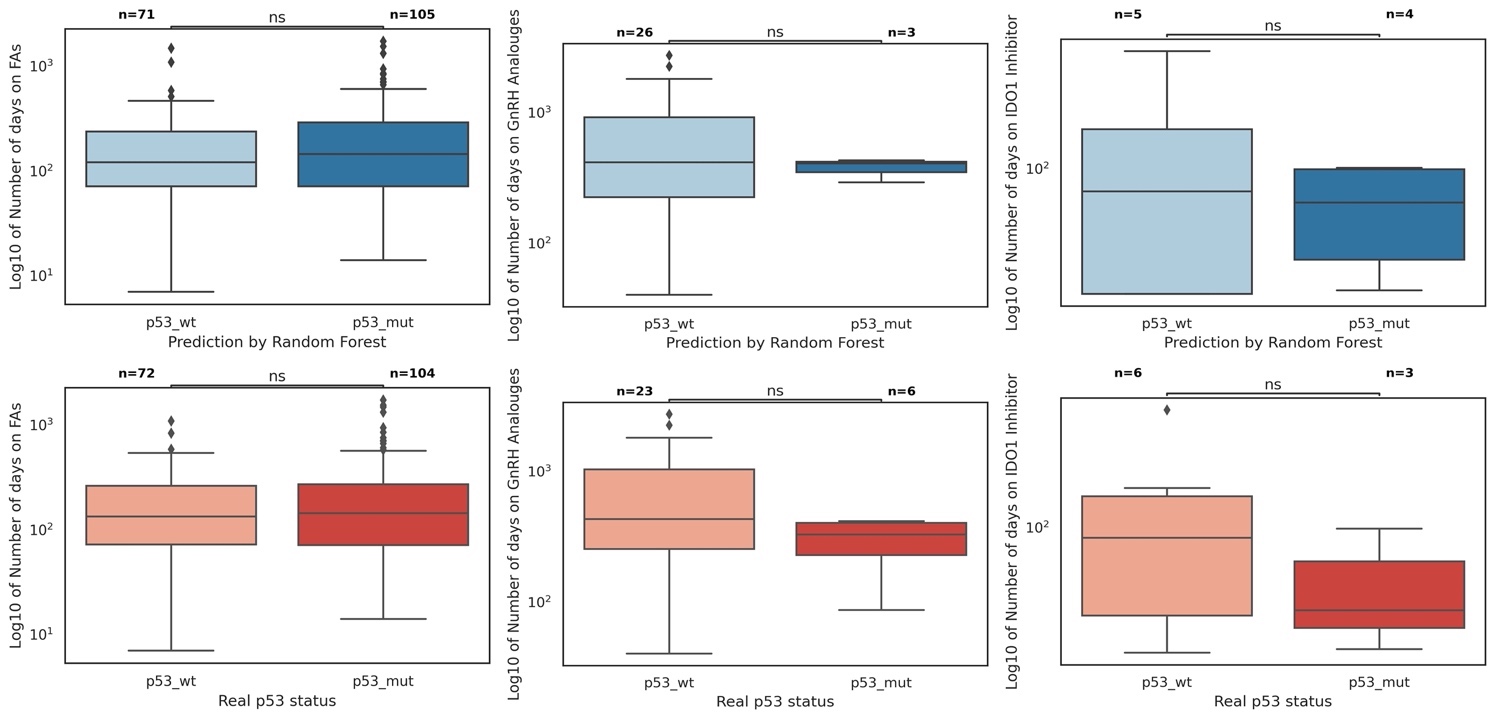


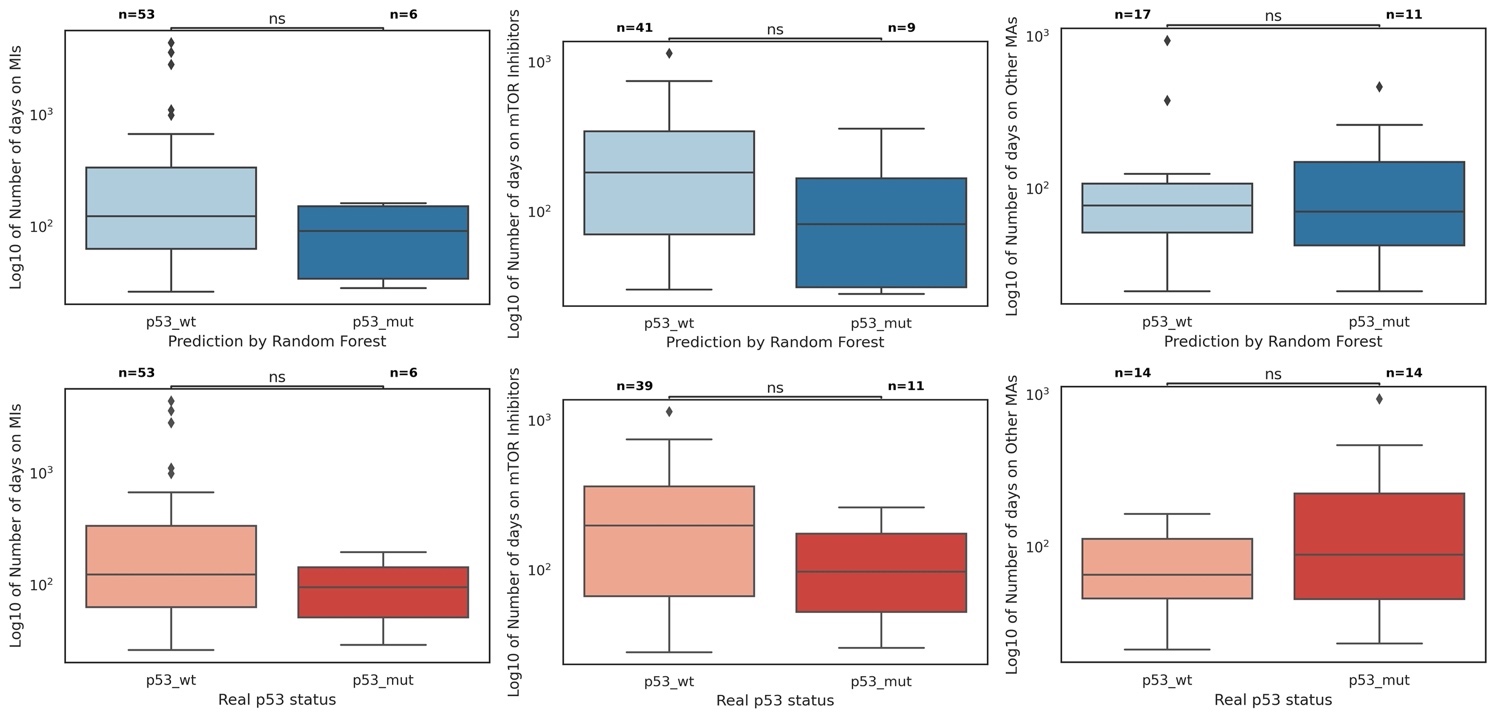


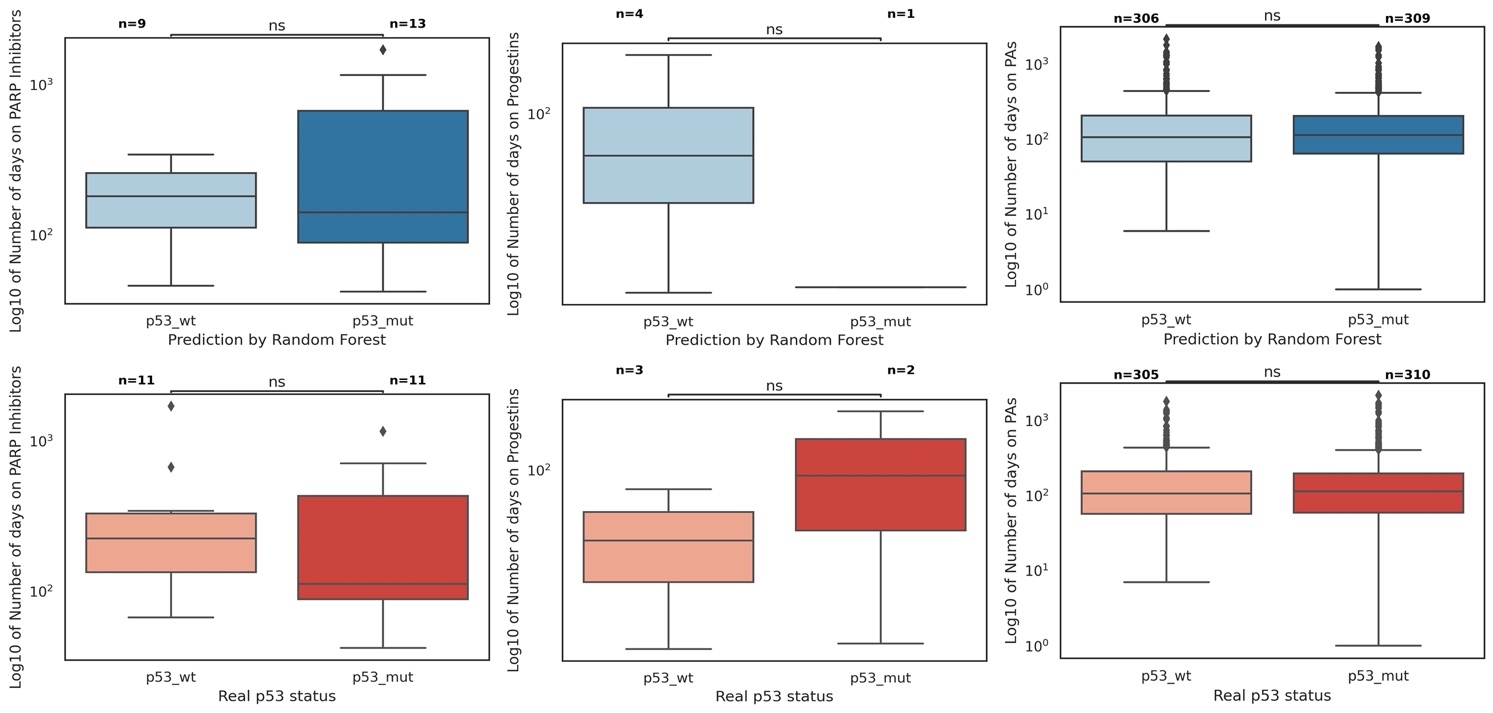


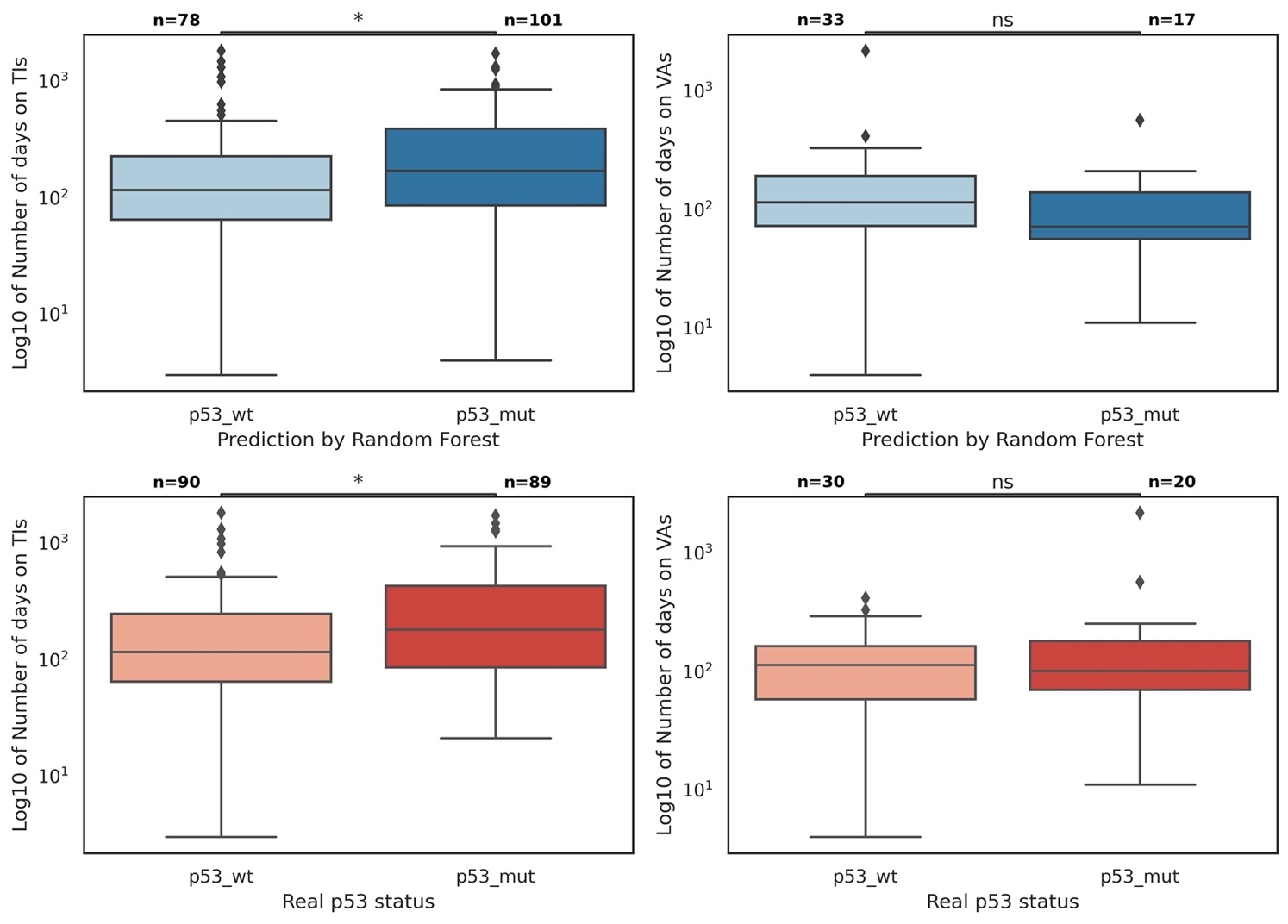


**Supplementary Figure 12.** The boxplots of log10 of the number of days on different treatment categories where the difference in data points divided by *TP53* mutation status and the predicted status by the random forest are statistically similar (AAs=Alkylating Agents, AntiHGs=Antihyperglycemic Agents, CAs=Cytotoxic Antibiotics, CDK4/6 Is=CDK4/6 Inhibitors, FAs=Folate Antagonists, MAs=Monoclonal Antibodies, MIs=Multikinase Inhibitors, PAs=Pyrimidine Analouges, TIs=Topoisomerase Inhibitors, VAs=Vinca Alkaloids) (the p-values are found in a Mann-Whitney-Wilcoxon two-sided test with Bonferroni correction; p-value annotation guide: ns: 5.00e-02 < p <= 1.00, *: 1.00e-02 < p <= 5.00e-2, **: 1.00e-03 < p <= 1.00e-02).

# References

1. Pedregosa F, Varoquaux G, Gramfort A, Michel V, Thirion B, Grisel O, et al. Scikit-learn: Machine Learning in Python. Journal ofMachine Learning Research. 2011;12:2825–30.

2. Waskom M. Seaborn: Statistical Data Visualization. Journal of Open Source Software. 2021;6(60):3021.

3. Hunter JD. Matplotlib: a 2D Graphics Environment. Computing in Science and Engineering. 2007;9(3):90–5.

4. Huang DW, Sherman BT, Lempicki RA. Bioinformatics enrichment tools: Paths toward the comprehensive functional analysis of large gene lists. Nucleic Acids Research. 2009;37(1):1–13.

5. Huang DW, Sherman BT, Lempicki RA. Systematic and integrative analysis of large gene lists using DAVID bioinformatics resources. Nature Protocols. 2009;4(1):44–57.

6. Robinson JT, Thorvaldsdóttir H, Winckler W, Guttman M, Lander ES, Getz G, et al. Integrative genomics viewer. Nature Biotechnology. 2011;29(1):24–6.

7. Vardanyan R, Hruby V. Antineoplastic Agents. In: Vardanyan R, Hruby V, editors. Synthesis of Best-Seller Drugs. Academic Press; 2016. p. 495–547.

8. Antineoplastic Agents [Internet]. LiverTox: Clinical and Research Information on Drug-Induced Liver Injury. Bethesda (MD): National Institute of Diabetes and Digestive and Kidney Diseases; 2012 [cited 2022 Jan 24]. Available from: https://www.ncbi.nlm.nih.gov/books/

9. Wishart DS, Feunang YD, Guo AC, Lo EJ, Marcu A, Grant JR, et al. DrugBank 5.0: A major update to the DrugBank database for 2018. Nucleic Acids Research. 2018;46(D1):D1074–82.

10. NCI Drug Dictionary [Internet]. National Cancer Institute. [cited 2022 Jan 24]. Available from: https://www.cancer.gov/publications/dictionaries

11. Tanikawa C, Ri C, Kumar V, Nakamura Y, Matsuda K. Crosstalk of EDA-A2/XEDAR in the p53 signaling pathway. Molecular Cancer Research. 2010;8(6):855–63.

12. Moyer SM, Wasylishen AR, Qi Y, Fowlkes N, Su X, Lozano G. P53 drives a transcriptional program that elicits a non-cell-autonomous response and alters cell state in vivo. Proc Natl Acad Sci U S A. 2020;117(38):23663–73.

13. Xiong X, Zhao Y, He H, Sun Y. Ribosomal protein S27-like and S27 interplay with p53-MDM2 axis as a target, a substrate and a regulator. Oncogene. 2011;30(15):1798–811.

14. Piette J, Neel H, Maréchal V. Mdm2: Keeping p53 under control. Oncogene. 1997;15(9):1001–10.

15. Nag S, Qin J, Srivenugopal KS, Wang M, Zhang R. The MDM2-p53 pathway revisited. Journal of Biomedical Research. 2013;27(4):254–71.

16. Musa J, Aynaud MM, Mirabeau O, Delattre O, Grünewald TG. MYBL2 (B-Myb): a central regulator of cell proliferation, cell survival and differentiation involved in tumorigenesis. Cell Death Dis [Internet]. 2017;8(6):e2895. Available from: http://dx.doi.org/10.1038/cddis.2017.244

17. Seong HA, Manoharan R, Ha H. B-MYB positively regulates serine-threonine kinase receptor-associated protein (STRAP) activity through direct interaction. Journal of Biological Chemistry [Internet]. 2011;286(9):7439–56. Available from: http://dx.doi.org/10.1074/jbc.M110.184382

18. Bajaj S, Alam SK, Roy KS, Datta A, Nath S, Roychoudhury S. E2 ubiquitin-conjugating enzyme, UBE2C gene, is reciprocally regulated by wild-type and gain-of-function mutant p53. Journal of Biological Chemistry [Internet]. 2016;291(27):14231–47. Available from: http://dx.doi.org/10.1074/jbc.M116.731398

19. Kidokoro T, Tanikawa C, Furukawa Y, Katagiri T, Nakamura Y, Matsuda K. CDC20, a potential cancer therapeutic target, is negatively regulated by p53. Oncogene. 2008;27(11):1562–71.

20. Banerjee T, Nath S, Roychoudhury S. DNA damage induced p53 downregulates Cdc20 by direct binding to its promoter causing chromatin remodeling. Nucleic Acids Research. 2009;37(8):2688–98.

21. Ashouri A, Sayin VI, van den Eynden J, Singh SX, Papagiannakopoulos T, Larsson E. Pan-cancer transcriptomic analysis associates long non-coding RNAs with key mutational driver events. Nat Commun [Internet]. 2016;7:13197. Available from: http://dx.doi.org/10.1038/ncomms13197

22. Walian PJ, Hang B, Mao JH. Prognostic significance of FAM83D gene expression across human cancer types. Oncotarget. 2016;7(3):3332–40.

23. Itoh T, O’Shea C, Linn S. Impaired Regulation of Tumor Suppressor p53 Caused by Mutations in the Xeroderma Pigmentosum DDB2 Gene: Mutual Regulatory Interactions between p48  DDB2  and p53 . Molecular and Cellular Biology. 2003;23(21):7540–53.

24. Zhou J, Guo H, Liu L, Hao S, Guo Z, Zhang F, et al. Construction of co-expression modules related to survival by WGCNA and identification of potential prognostic biomarkers in glioblastoma. Journal of Cellular and Molecular Medicine. 2021;25(3):1633–44.

25. Hou S, Chen X, Li M, Huang X, Liao H, Tian B. Higher expression of cell division cycle-associated protein 5 predicts poorer survival outcomes in hepatocellular carcinoma. Aging. 2020;12(14):14542–55.

26. Filipescu D, Naughtin M, Podsypanina K, Lejour V, Wilson L, Gurard-Levin ZA, et al. Essential role for centromeric factors following p53 loss and oncogenic transformation. Genes and Development. 2017;31(5):463–80.

27. Jeffery D, Gatto A, Podsypanina K, Renaud-Pageot C, Ponce Landete R, Bonneville L, et al. CENP-A overexpression promotes distinct fates in human cells, depending on p53 status. Communications Biology [Internet]. 2021;4(1):1–18. Available from: http://dx.doi.org/10.1038/s42003-021-01941-5

28. Mirza A, Wu Q, Wang L, McClanahan T, Bishop WR, Gheyas F, et al. Global transcriptional program of p53 target genes during the process of apoptosis and cell cycle progression. Oncogene. 2003;22(23):3645–54.

29. Ma J, Liu X, Liu P, Lu W, Shen X, Ma R, et al. Identification of a new p53 responsive element in the promoter region of anillin. International Journal of Molecular Medicine. 2020;45(5):1563–70.

30. Kawase T, Ichikawa H, Ohta T, Nozaki N, Tashiro F, Ohki R, et al. p53 target gene AEN is a nuclear exonuclease required for p53-dependent apoptosis. Oncogene. 2008;27(27):3797–810.

31. Date DA, Jacob CJ, Bekier ME, Stiff AC, Jackson MW, Taylor WR. Borealin is repressed in response to p53/Rb signaling. Cell Biology International. 2007;31(12):1470–81.

32. Gao X, Wen X, He H, Zheng L, Yang Y, Yang J, et al. Knockdown of CDCA8 inhibits the proliferation and enhances the apoptosis of bladder cancer cells. PeerJ. 2020;2020(4):1–26.

33. Ando K, Ozaki T, Yamamoto H, Furuya K, Hosoda M, Hayashi S, et al. Polo-like kinase 1 (Plk1) inhibits p53 function by physical interaction and phosphorylation. Journal of Biological Chemistry [Internet]. 2004;279(24):25549–61. Available from: http://dx.doi.org/10.1074/jbc.M314182200

34. Bornstein C, Brosh R, Molchadsky A, Madar S, Kogan-Sakin I, Goldstein I, et al. SPATA18, a Spermatogenesis-Associated Gene, Is a Novel Transcriptional Target of p53 and p63. Molecular and Cellular Biology. 2011;31(8):1679–89.

35. Yu Q, Pu SY, Wu H, Chen XQ, Jiang JJ, Gu KS, et al. TICRR contributes to tumorigenesis through accelerating DNA replication in cancers. Frontiers in Oncology. 2019;9(JUN):8–10.

36. Shimo A, Tanikawa C, Nishidate T, Lin ML, Matsuda K, Park JH, et al. Involvement of kinesin family member 2C/mitotic centromere-associated kinesin overexpression in mammary carcinogenesis. Cancer Science. 2008;99(1):62–70.

37. Müller GA, Wintsche A, Stangner K, Prohaska SJ, Stadler PF, Engeland K. The CHR site: Definition and genome-wide identification of a cell cycle transcriptional element. Nucleic Acids Research. 2014;42(16):10331-10350A.

38. Malilas W, Koh SS, Srisuttee R, Boonying W, Cho IR, Jeong CS, et al. Cancer upregulated gene 2, a novel oncogene, confers resistance to oncolytic vesicular stomatitis virus through STAT1-OASL2 signaling. Cancer Gene Therapy. 2013;20(2):125–32.

39. Townsend PA, Scarabelli TM, Davidson SM, Knight RA, Latchman DS, Stephanou A. STAT-1 Interacts with p53 to Enhance DNA Damage-induced Apoptosis. Journal of Biological Chemistry [Internet]. 2004;279(7):5811–20. Available from: http://dx.doi.org/10.1074/jbc.M302637200

40. Chang YC, Wu CH, Yen TC, Ouyang P. Centrosomal protein 55 (Cep55) stability is negatively regulated by p53 protein through polo-like kinase 1 (Plk1). Journal of Biological Chemistry [Internet]. 2012;287(6):4376–85. Available from: http://dx.doi.org/10.1074/jbc.M111.289108

41. Lee HN, Ahn SM, Jang HH. Cold-inducible RNA-binding protein, CIRP, inhibits DNA damage-induced apoptosis by regulating p53. Biochemical and Biophysical Research Communications [Internet]. 2015;464(3):916–21. Available from: http://dx.doi.org/10.1016/j.bbrc.2015.07.066

42. Chen M, Zhang H, Zhang G, Zhong A, Ma Q, Kai J, et al. Targeting TPX2 suppresses proliferation and promotes apoptosis via repression of the PI3k/AKT/P21 signaling pathway and activation of p53 pathway in breast cancer. Biochemical and Biophysical Research Communications [Internet]. 2018;507(1–4):74–82. Available from: https://doi.org/10.1016/j.bbrc.2018.10.164

43. Yang X, Liu G, Xiao H, Yu F, Xiang X, Lu Y, et al. TPX2 overexpression in medullary thyroid carcinoma mediates TT cell proliferation. Pathology and Oncology Research. 2014;20(3):641–8.

44. Liu G, Chen X. The ferredoxin reductase gene is regulated by the p53 family and sensitizes cells to oxidative stress-induced apoptosis. Oncogene. 2002;21(47):7195–204.

45. Zhang Y, Xiong Y, Yarbrough WG. ARF promotes MDM2 degradation and stabilizes p53: ARF-INK4a locus deletion impairs both the Rb and p53 tumor suppression pathways. Cell. 1998;92(6):725–34.

46. Møller MB, Ino Y, Gerdes AM, Skjødt K, Louis DN, Pedersen NT. Aberrations of the p53 pathway components p53, MDM2 and CDKN2A appear independent in diffuse large B cell lymphoma. Leukemia. 1999;13(3):453–9.

47. LIEU AS, CHENG TS, CHOU CH, WU CH, HSU CY, HUANG CYF, et al. Functional characterization of AIBp, a novel Aurora-A binding protein in centrosome structure and spindle formation. International Journal of Oncology. 2010;37(100):429–36.

48. Chou CH, Loh JK, Yang MC, Lin CC, Hong MC, Cho CL, et al. AIBp regulates mitotic entry and mitotic spindle assembly by controlling activation of both Aurora-A and Plk1. Cell Cycle. 2015;14(17):2764–76.

49. Lou J, Chen H, Han J, He H, Huen MSY, Feng XH, et al. AUNIP/C1orf135 directs DNA double-strand breaks towards the homologous recombination repair pathway. Nature Communications [Internet]. 2017;8(1). Available from: http://dx.doi.org/10.1038/s41467-017-01151-w

50. Huang YF, Chang MDT, Shieh SY. TTK/hMps1 Mediates the p53-Dependent Postmitotic Checkpoint by Phosphorylating p53 at Thr18. Molecular and Cellular Biology. 2009;29(11):2935–44.

51. Wang Y, Zhang C, Mai L, Niu Y, Wang Y, Bu Y. PRR11 and SKA2 gene pair is overexpressed and regulated by p53 in breast cancer. BMB Reports. 2019;52(2):157–62.

52. Wang Y, Weng H, Zhang Y, Long Y, Li Y, Niu Y, et al. The PRR11-SKA2 bidirectional transcription unit is negatively regulated by p53 through NF-Y in lung cancer cells. International Journal of Molecular Sciences. 2017;18(3).

53. Chen C, Tang Y, Qu WD, Han X, Zuo J bin, Cai QY, et al. Evaluation of clinical value and potential mechanism of MTFR2 in lung adenocarcinoma via bioinformatics. BMC Cancer. 2021;21(1):1–14.

54. Chung JH, Larsen AR, Chen E, Bunz F. A PTCH1 homolog transcriptionally activated by p53 suppresses hedgehog signaling. Journal of Biological Chemistry [Internet]. 2014;289(47):33020–31. Available from: http://dx.doi.org/10.1074/jbc.M114.597203

55. Sun X, He Z, Guo L, Wang C, Lin C, Ye L, et al. ALG3 contributes to stemness and radioresistance through regulating glycosylation of TGF-β receptor II in breast cancer. Journal of Experimental and Clinical Cancer Research. 2021;40(1):1–25.

56. Seong HA, Ha H. Murine protein serine-threonine kinase 38 activates p53 function through Ser 15 phosphorylation. Journal of Biological Chemistry [Internet]. 2012;287(25):20797–810. Available from: http://dx.doi.org/10.1074/jbc.M112.347757

57. Gu C, Banasavadi-Siddegowda YK, Joshi K, Nakamura Y, Kurt H, Gupta S, et al. Tumor-specific activation of the C-JUN/MELK pathway regulates glioma stem cell growth in a p53-dependent manner. Stem Cells. 2013;31(5):870–81.

58. Bollu LR, Shepherd J, Zhao D, Ma Y, Tahaney W, Speers C, et al. Mutant P53 induces MELK expression by release of wild-type P53-dependent suppression of FOXM1. npj Breast Cancer [Internet]. 2020;6(1):1–12. Available from: http://dx.doi.org/10.1038/s41523-019-0143-5

59. Ganguly R, Mohyeldin A, Thiel J, Kornblum HI, Beullens M, Nakano I. MELK—a conserved kinase: functions, signaling, cancer, and controversy. Clinical and Translational Medicine. 2015;4(1).

60. Adimoolam S, Ford JM. p53 and DNA damage-inducible expression of the xeroderma pigmentosum group C gene. Proc Natl Acad Sci U S A. 2002;99(20):12985–90.

61. Barckhausen C, Roos WP, Naumann SC, Kaina B. Malignant melanoma cells acquire resistance to DNA interstrand cross-linking chemotherapeutics by p53-triggered upregulation of DDB2/XPC-mediated DNA repair. Oncogene. 2014;33(15):1964–74.

62. Wang QE, Zhu Q, Wani MA, Wani G, Chen J, Wani AA. Tumor suppressor p53 dependent recruitment of nucleotide excision repair factors XPC and TFIIH to DNA damage. DNA Repair. 2003;2(5):483–99.

63. Krzeszinski JY, Choe V, Shao J, Bao X, Cheng H, Luo S, et al. XPC promotes MDM2-mediated degradation of the p53 tumor suppressor. Molecular Biology of the Cell. 2014;25(2):213–21.

64. Engeland K. Cell cycle arrest through indirect transcriptional repression by p53: I have a DREAM. Cell Death and Differentiation [Internet]. 2018;25(1):114–32. Available from: http://dx.doi.org/10.1038/cdd.2017.172

65. Mirza A, McGuirk M, Hockenberry TN, Wu Q, Ashar H, Black S, et al. Human survivin is negatively regulated by wild-type p53 and participates in p53-dependent apoptotic pathway. Oncogene. 2002;21(17):2613–22.

66. Yu L, Xiao Y, Zhou X, Wang J, Chen S, Peng T, et al. TRIP13 interference inhibits the proliferation and metastasis of thyroid cancer cells through regulating TTC5/p53 pathway and epithelial-mesenchymal transition related genes expression. Biomedicine and Pharmacotherapy [Internet]. 2019;120(August):109508. Available from: https://doi.org/10.1016/j.biopha.2019.109508

67. Li C, Xia J, Franqui-Machin R, Chen F, He Y, Ashby TC, et al. TRIP13 modulates protein deubiquitination and accelerates tumor development and progression of B cell malignancies. Journal of Clinical Investigation. 2021;131(14).

68. Okamoto K, Beach D. Cyclin G is a transcriptional target of the p53 tumor suppressor protein. EMBO Journal. 1994;13(20):4816–22.

69. Okamoto K, Li H, Jensen MR, Zhang T, Taya Y, Thorgeirsson SS, et al. Cyclin G recruits PP2A to dephosphorylate Mdm2. Molecular Cell. 2002;9(4):761–71.

70. Katkoori VR, Shanmugam C, Jia X, Vitta SP, Sthanam M, Callens T, et al. Prognostic significance and gene expression profiles of p53 mutations in microsatellite-stable Stage III colorectal adenocarcinomas. PLoS ONE. 2012;7(1).

71. Quaas M, Müller GA, Engeland K. p53 can repress transcription of cell cycle genes through a p21 WAF1/CIP1-dependent switch from MMB to DREAM protein complex binding at CHR promoter elements. Cell Cycle. 2012;11(24):4661–72.

72. Liu Q, Kaneko S, Yang L, Feldman RI, Nicosia S v., Chen J, et al. Aurora-A abrogation of p53 DNA binding and transactivation activity by phosphorylation of serine 215. Journal of Biological Chemistry [Internet]. 2004;279(50):52175–82. Available from: http://dx.doi.org/10.1074/jbc.M406802200

73. Katayama H, Sasai K, Kawai H, Yuan ZM, Bondaruk J, Suzuki F, et al. Phosphorylation by aurora kinase A induces Mdm2-mediated destabilization and inhibition of p53. Nature Genetics. 2004;36(1):55–62.

74. Mao J hua, Perez-losada J, Wu D, Delrosario R. Haploinsufficient Tumour Suppressor Gene. 2004;775–9.

75. Perez-Losada J, Mao JH, Balmain A. Control of genomic instability and epithelial tumor development by the p53-Fbxw7/Cdc4 pathway. Cancer Research. 2005;65(15):6488–92.

76. Sugimasa H, Taniue K, Kurimoto A, Takeda Y, Kawasaki Y, Akiyama T. Heterogeneous nuclear ribonucleoprotein K upregulates the kinetochore complex component NUF2 and promotes the tumorigenicity of colon cancer cells. Biochemical and Biophysical Research Communications [Internet]. 2015;459(1):29–35. Available from: http://dx.doi.org/10.1016/j.bbrc.2015.02.043

77. Moumen A, Masterson P, O’Connor MJ, Jackson SP. hnRNP K: An HDM2 target and transcriptional coactivator of p53 in response to DNA damage. Cell. 2005;123(6):1065–78.

78. Chaudhary R, Wang X, Cao B, de La Iglesia J, Masannat J, Song F, et al. Long noncoding RNA, LINC00460, as a prognostic biomarker in head and neck squamous cell carcinoma (HNSCC). American Journal of Translational Research. 2020;12(2):684–96.

79. Srsen V, Gnadt N, Dammermann A, Merdes A. Inhibition of centrosome protein assembly leads to p53-dependent exit from the cell cycle. Journal of Cell Biology. 2006;174(5):625–30.

80. Kuo TC, Chang PY, Huang SF, Chou CK, Chao CCK. Knockdown of HURP inhibits the proliferation of hepacellular carcinoma cells via downregulation of gankyrin and accumulation of p53. Biochemical Pharmacology [Internet]. 2012;83(6):758–68. Available from: http://dx.doi.org/10.1016/j.bcp.2011.12.034

81. Chao CCK. Inhibition of apoptosis by oncogenic hepatitis B virus X protein: Implications for the treatment of hepatocellular carcinoma. World Journal of Hepatology. 2016;8(25):1061–6.

82. Ke MJ, Ji LD, Li YX. Bioinformatics analysis combined with experiments to explore potential prognostic factors for pancreatic cancer. Cancer Cell International [Internet]. 2020;20(1):1–13. Available from: https://doi.org/10.1186/s12935-020-01474-7

83. Kawase T, Ohki R, Shibata T, Tsutsumi S, Kamimura N, Inazawa J, et al. PH Domain-Only Protein PHLDA3 Is a p53-Regulated Repressor of Akt. Cell [Internet]. 2009;136(3):535–50. Available from: http://dx.doi.org/10.1016/j.cell.2008.12.002

84. Wang C jing, Li X, Shi P, Ding H yan, Liu Y ping, Li T, et al. Holliday junction recognition protein promotes pancreatic cancer growth and metastasis via modulation of the MDM2/p53 signaling. Cell Death and Disease [Internet]. 2020;11(5). Available from: http://dx.doi.org/10.1038/s41419-020-2595-9

85. Itzel T, Scholz P, Maass T, Krupp M, Marquardt JU, Strand S, et al. Translating bioinformatics in oncology: Guilt-by-profiling analysis and identification of KIF18B and CDCA3 as novel driver genes in carcinogenesis. Bioinformatics. 2015;31(2):216–24.

86. Wu L, Ma CA, Zhao Y, Jain A. Aurora B interacts with NIR-p53, leading to p53 phosphorylation in its DNA-binding domain and subsequent functional suppression. Journal of Biological Chemistry [Internet]. 2011;286(3):2236–44. Available from: http://dx.doi.org/10.1074/jbc.M110.174755

87. Gully CP, Velazquez-Torres G, Shin JH, Fuentes-Mattei E, Wang E, Carlock C, et al. Aurora B kinase phosphorylates and instigates degradation of p53. Proc Natl Acad Sci U S A. 2012;109(24):1513–22.

88. Marxer M, Ma HT, Man WY, Poon RYC. P53 deficiency enhances mitotic arrest and slippage induced by pharmacological inhibition of Aurora kinases. Oncogene. 2014;33(27):3550–60.

89. Zawacka-Pankau J, Grinkevich V v., Hünten S, Nikulenkov F, Gluch A, Li H, et al. Inhibition of glycolytic enzymes mediated by pharmacologically activated p53: Targeting Warburg effect to fight cancer. Journal of Biological Chemistry. 2011;286(48):41600–15.

90. Li BS, Jin AL, Zhou Z, Seo JH, Choi BM. DRG2 Accelerates Senescence via Negative Regulation of SIRT1 in Human Diploid Fibroblasts. Oxidative Medicine and Cellular Longevity. 2021;2021:1–16.

91. Shinozaki S, Chang K, Sakai M, Shimizu N, Yamada M, Tanaka T, et al. Inflammatory stimuli induce inhibitory S-nitrosylation of the deacetylase SIRT1 to increase acetylation and activation of p53 and p65. Science Signaling. 2014;7(351):1–30.

92. Han JW, Flemington C, Houghton AB, Gu Z, Zambetti GP, Lutz RJ, et al. Expression of bbc3, a pro-apoptotic BH3-only gene, is regulated by diverse cell death and survival signals. Proc Natl Acad Sci U S A. 2001;98(20):11318–23.

93. Nakano K, Vousden KH. PUMA, a novel proapoptotic gene, is induced by p53. Molecular Cell. 2001;7(3):683–94.

94. Yu J, Zhang L, Hwang PM, Kinzler KW, Vogelstein B. PUMA Induces the Rapid Apoptosis of Colorectal Cancer Cells. Molecular Cell. 2001;7(3):673–82.

95. Hu WL, Jin L, Xu A, Wang YF, Thorne RF, Zhang XD, et al. GUARDIN is a p53-responsive long non-coding RNA that is essential for genomic stability. Nature Cell Biology. 2018;20(4):492–502.

96. Lin T, Hou PF, Meng S, Chen F, Jiang T, Li M le, et al. Emerging roles of p53 related lncRNAs in cancer progression: A systematic review. International Journal of Biological Sciences. 2019;15(6):1287–98.

97. El-Deiry WS, Tokino T, Velculescu VE, Levy DB, Parsons R, Trent JM, et al. WAF1, a potential mediator of p53 tumor suppression. Cell. 1993;75(4):817–25.

98. Mlynarczyk C, Fåhraeus R. Endoplasmic reticulum stress sensitizes cells to DNA damage-induced apoptosis through p53-dependent suppression of p21 CDKN1A. Nature Communications. 2014;5.

99. Miyashita T, Reed JC. Tumor suppressor p53 is a direct transcriptional activator of the human bax gene. Cell. 1995;80(2):293–9.
